# Identification of MAPK12 as a Prognostic Biomarker for Esophageal Carcinoma Using Bioinformatics and Machine Learning

**DOI:** 10.1155/bmri/2605071

**Published:** 2025-12-29

**Authors:** Shuyuan Gu, Xinyang Yan, Shihui Chen, Zepeng Dong, Xiaopeng Li, Changchun Ye, Chenye Zhao, Hang Yuan, Xuejun Sun, Wei Zhao, Peng Zhang

**Affiliations:** ^1^ Department of General Surgery, Xi′an No. 9 Hospital, Xi′an, Shaanxi Province, China; ^2^ Department of Neurosurgery, The First Affiliated Hospital of Xi′an Jiaotong University, Xi′an, Shaanxi Province, China, xjtu.edu.cn; ^3^ Center for Precision Cancer Medicine, MED-X Institute, The First Affiliated Hospital of Xi′an Jiaotong University, Xi′an, Shaanxi Province, China, xjtu.edu.cn; ^4^ Department of General Surgery, The First Affiliated Hospital of Xi′an Jiaotong University, Xi′an, Shaanxi Province, China, xjtu.edu.cn; ^5^ Department of Thoracic Surgery, Baoji People′s Hospital, Baoji, Shaanxi Province, China

**Keywords:** bioinformatics, esophageal carcinoma, prognosis, signature, telomere

## Abstract

To develop a telomere‐related prognostic signature for esophageal carcinoma (ESCA), we integrated bioinformatics and machine learning approaches. Hub genes were identified from overlapping differentially expressed genes (DEGs). A prognostic model was constructed using LASSO and multivariate Cox regression, validated in independent GEO datasets, and further verified through cytological experiments. We also elucidated the mechanism by which MAPK12 promotes ESCA migration. The model robustly predicted survival of patients with ESCA, supported by both high‐throughput data and experimental evidence. Our findings highlight MAPK12 as a promising biomarker and provide a theoretical basis for understanding ESCA pathogenesis and developing targeted therapies.

## 1. Introduction

As the leading cause of cancer‐related mortality among all malignant tumors, esophageal carcinoma still contributes to a heavy burden not only for patients but also for all of society [1, 2]. In China, esophageal carcinoma is the fifth and twelfth most commonly diagnosed cancer in men and women, separately [3]. Because of the lack of early diagnosis, most patients (~75%) are diagnosed at late stages, which has resulted in only about 20%–25% five‐year survival rate for decades without great improvement [4]. Telomeres, consisting of repetitive TTAGGG DNA sequences and associated shelterin complexes, play essential roles in maintaining chromosomal integrity [5]. Accumulating evidence from genome‐wide association studies indicates that telomere‐related variants contribute significantly to the development and progression of human diseases, including cancer [6]. During this process, abnormal nuclear morphologies induced by telomere dysfunction might be an important reason [7]. Telomere‐related genes (TRGs) play a vital role in protecting chromosomal structure [8]. Moreover, many studies have been conducted to investigate the role of telomeres in the development and progression of cancers. The prognostic significance of telomere genes has been revealed in breast carcinoma [9]. A meta‐analysis indicated that short telomere length was associated with increased mortality risk and poor prognosis in patients with cancer [10]. Based on that, the prognostic model established based on TRGs has more advantages in predicting the prognosis of patients with cancer compared with other models [11]. In kidney cancer, the telomere length in tumor cells was shorter than that in normal kidney cells, but the prognostic role of telomere length remains controversial [12]. The pathogenesis and progression of oral cavity squamous cell carcinoma is a vital process because of TRG mutations. This finding reinforces the significance of a prognostic model that utilizes TRGs in squamous cell carcinoma [13]. Despite these advances, it remains unclear whether TRGs can be utilized to construct a robust prognostic model for ESCA.

**Figure 1:**
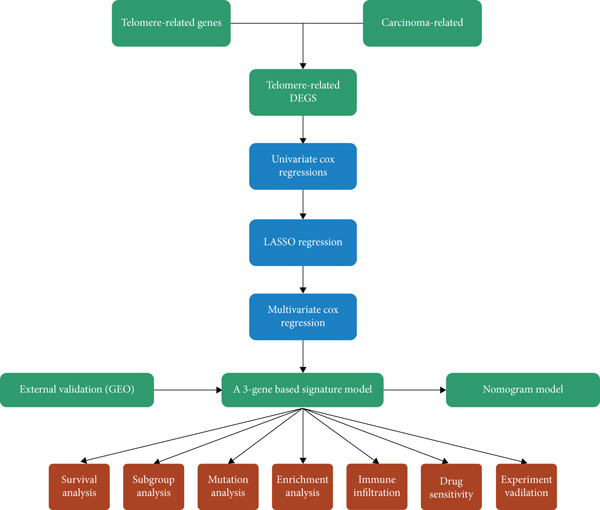
The flow diagram of the present study.

To address this gap, our study aims to develop and validate a TRG‐based signature for predicting outcomes in patients with ESCA. Using data from the TCGA cohort (https://www.cancer.gov/tcga/), we first identified differentially expressed TRGs between ESCA samples and normal controls. To establish the signature model, we applied LASSO regression and multivariate Cox regression to ESCA‐associated DE‐TRGs. To validate the results, we screened GEO datasets externally. To explore the potential mechanisms of survival differences in different risk populations, functional enrichment analysis and somatic mutation analysis were carried out. We analyzed the correlation between risk scores, immune infiltration levels, and drug sensitivity. This study offers novel insights into the prognostic predictability of TRGs in ESCA and may contribute to the development of personalized treatment strategies. The present study has a flow chart that summarizes it in Figure 1.

## 2. Materials and Methods

### 2.1. Data Acquisition and Preprocessing

The clinical information and RNA‐sequencing data of patients with ESCA were obtained from the TCGA data portal (https://www.cancer.gov/tcga/). All Fragments Per Kilobase Million (FPKM) transcriptome data were log‐transformed and converted to transcripts per million (TPM) before analysis.

For external validation, independent datasets [GSE53625 [14]] along with clinical information were downloaded from the GEO database (https://www.ncbi.nlm.nih.gov/geo/). The following inclusion criteria were applied for screening the qualified GEO datasets: (1) complete gene expression profiles and survival information limited to patients with ESCA; (2) the raw gene expression data could be downloaded in CEL files and included the corresponding probe information; and (3) the total number of samples was no less than 50. Following these guidelines, the raw matrix data were log2‐transformed, quantile normalized, and averaged over duplicate genes using the “limma” package (Version 3.52.4) in R software. This meticulous process ensured the reliability of our analysis, with the clinical characteristics of patients with ESCA in both the TCGA and GEO datasets detailed in Supplementary Table S1.

### 2.2. Identification of Differentially Expressed Genes (DEGs)

The “DESeq2” R package (Version 1.36.0) was applied to perform differential gene expression analysis between esophageal carcinoma and normal tissues in the TCGA dataset. The DEGs with absolute fold changes (|*l* *o* *g* *F* *C*|) > 1.5 and adjusted *p* values < 0.05 were selected. TRG data was downloaded from http://www.cancertelsys.org/telnet/ [15]. The detailed information on the TRGs is provided in Supplementary Table S2. By intersecting this list with the DEGs of ESCA, we identified telomere‐related DEGs for our subsequent analyses.

### 2.3. Model Construction and Validation

First, univariate Cox regression (“survival” R package, Version 3.5.7) was conducted based on the telomere‐related DEGs to screen the prognosis‐related genes in the TCGA training set. Then, we performed LASSO regression was applied with 10‐fold cross‐validation, using the “glmnet” R package (Version 4.1.8) to narrow down candidate genes. Last, multivariate Cox regression analysis was conducted to identify the most significant genes and establish a prognostic signature. Subsequently, the risk model was constructed based on the gene expression values and coefficients, which were acquired via multivariate Cox regression using the Akaike Information Criterion (AIC) method. The specific calculation formula for the risk score was as follows:

RiskScore=∑i=1n Coefficientβi∗xi

Patients with ESCA were stratified into low‐ and high‐risk groups based on their median risk score. Subsequently, Kaplan–Meier survival curves (“survminer” and “survival”) and time‐dependent receiver operating characteristic (ROC) curves (“pROC” R package, Version 1.18.4) were employed to assess the prognostic value of the clinical model. For external validation, independent datasets from GEO (GSE53625) were used to verify the robustness of the novel signature.

### 2.4. Construction and Validation of a Nomogram

We used multivariate Cox regression analysis to construct a predictive nomogram model (using the “rms”,Version 6.7.0, and “survival” R packages) for predicting the 3‐, 4‐, and 5‐year overall survival probability in patients with ESCA [16]. Calibration curves were generated to assess the accuracy of the nomogram. For an ideal predictive model, the predictive results are expected to fall on the 45‐degree diagonal line of the calibration plot and have a higher *C*‐index in the Harrell concordance test. Decision curve analysis (DCA) [17] was also performed to measure the net clinical benefits of the nomogram model.

### 2.5. Mutation Analysis of the Prognostic Model

The TCGA somatic mutation data of ESCA patients were downloaded from the UCSC Xena browser (https://xenabrowser.net/). The differences in somatic mutation data between the high‐ and low‐risk groups were analyzed and are presented in the form of waterfall charts (“maftools” R package, Version 2.12.0). Tumor mutation burden (TMB) is defined as the number of tumor mutations per megabase in each tumor sample. The corresponding TMB values were calculated by the “tmb” function in the “maftool” R package and log‐transformed for visualization.

### 2.6. Functional Annotation and Enrichment Analyses

Telomere‐related DEGs were subjected to Gene Ontology (GO) and Kyoto Encyclopedia of Genes and Genomes (KEGG) enrichment analyses (“clusterProfiler” R package, Version 4.8.3). Gene set enrichment analysis (GSEA) was performed to assess the potential differences in biological functions between different risk groups, as defined by the C2 (c2.cp.kegg.v7.4.symbols.gmt) subset retrieved from the Molecular Signature Database (MsigDB, https://www.gsea-msigdb.org/gsea/msigdb/) [18]. For GSEA, terms with an adjusted *p* value < 0.05 and a false discovery rate (FDR) < 0.25 were considered significant. Moreover, the gene set variation analysis (GSVA) algorithm was applied based on 50 hallmark pathways described in the Molecular Signature Database to identify enriched signaling pathways between the low‐risk and high‐risk groups (“GSVA” R package, Version 1.44.5) [19].

### 2.7. Tumor Immune Infiltration Analysis

We calculate the tumor immune scores, stroma scores, tumor purity score, and ESTIMATE score with the “Estimate” R package (Version 1.0.13) [20]. The TIDE, dysfunction, and exclusion scores were obtained from the Tumor Immune Dysfunction and Exclusion website (TIDE, http://tide.dfci.harvard.edu/) [21]. In addition, CIBERSORT (https://cibersort.stanford.edu/) [22] was performed to quantify the relative immune cell infiltration levels and immune function between the low‐ and high‐risk groups.

### 2.8. Drug Sensitivity Analysis

For the drug sensitivity analysis, we obtained an analyzed dataset of six commonly used chemotherapeutic drugs (cisplatin, docetaxel, paclitaxel, gemcitabine, vinorelbine, and bleomycin) for esophageal cancer from the Genomics of Drug Sensitivity in Cancer database (GDSC, https://www.cancerrxgene.org/) [23]. The “pRRophetic” R package (Version 0.5) was utilized to estimate the half inhibitory concentration (IC50) of each chemotherapeutic drug.

### 2.9. Cell Lines and Cell Culture

The ECA109 and KYSE160 cell lines were provided by the Cell Resource Center of Shanghai Life Sciences Institute. ECA109 and KYSE160 cells were maintained in 1640 medium supplemented with 80 U/L penicillin and 0.08 mg/mL streptomycin. Ten percent of fetal bovine serum was also added to the medium. The cells were cultured in a conventional incubator at 37°C in a 5% CO_2_ atmosphere.

### 2.10. Cell Transfection

Small interfering RNA (siRNA) targeting human *MAPK12* and negative control siRNA (siNC) were purchased from Tsingke Biological Technology. The transient transfection of siRNA was performed according to the manufacturer’s instructions. First, ECA109 and KYSE160 cells were seeded the day before transfection at 30%–50% confluency. siRNA duplexes were diluted into reduced serum media Opti‐MEM. Then, the transfection reagent Lipo 8000 (Invitrogen) was added to the siRNA solution, which was then vortex mixed and incubated for 30 min at room temperature. Finally, Lipo8000‐siRNA complexes were added to fresh medium, and the cells were incubated at 37°C. Twenty‐four hours later, the transfected cells were collected for further experiments. The knockdown efficiency of the siRNAs was tested by Western Blotting.

### 2.11. Cell Proliferation Assay

A Cell Counting Kit‐8 (CCK‐8, Dojindo, Japan) was used to assess cell proliferation ability as instructed by the manufacturer. Cells were plated into 24‐well plates (6 × 10^4^ cells/well) for the indicated durations. Ten microliters of CCK‐8 solution was added to each well, followed by incubation for an additional hour. Finally, the absorbance was measured at 450 nm with a microplate reader (Bio‐Gene, China).

### 2.12. Wound Healing Assay

A wound healing assay was carried out to assess cell migration. Briefly, monolayer cells were wounded by scratching the surface of each well as uniformly as possible with a sterile 200 *μ*L pipette tip. The wells were then rinsed with phosphate‐buffered saline three times and incubated at 37°C for 48 h. Images of the initial wound and the movement of cells into the scratched area were captured using an inverted microscope equipped with a digital imaging system (Leica Microsystems GmbH, Wetzlar, Germany).

### 2.13. Transwell Migration Assay

Twenty‐four well transwell chambers (BD Biosciences, San Jose, CA, United States) with 8‐*μ*m pores were used to assess cell migration. Cells (1 × 10^4^ cells/well) in serum‐free medium were seeded into the upper chamber. The complete growth medium was added to the lower chamber as a chemoattractant. After culturing for 48 h at 37°C, noninvasive cells in the upper chamber were carefully removed with cotton swabs, and invasive cells on the lower membrane surface were fixed in methanol and stained with 0.1% crystal violet (Sigma–Aldrich) for 15 min. Finally, the invasive cells were photographed and counted under a microscope (Nikon, Tokyo, Japan).

### 2.14. Colony Formation

Cells in the logarithmic growth phase were seeded in 6 cm petri dishes at 1000 cells/well. Multiple pores were set up, and the cell state was observed every 3 days. After 7 days of culture, the cell colonies were washed with PBS twice, fixed with 4% paraformaldehyde, stained with Giemsa (Sigma, United States), and then washed twice with ddH_2_O. The number of cell colonies was observed under a microscope.

### 2.15. Western Blot

Total protein was extracted from ECA109 and KYSE160 cells, and the protein content was determined by BCA kits (Beyotime, Shanghai, China). Samples with the same protein content were boiled for 10 min, transferred to SDS–PAGE, sealed overnight, washed with primary antibody, incubated with primary antibody, washed with secondary antibody, and visualized by enhanced chemiluminescence (ECL) reagents. The results were analyzed by a Tanon chemiluminescence imaging analysis system. The MAPK12 antibody was purchased from Shenzhen Youpin Biotechnology Co. Ltd. (Shenzhen, China). *β*‐Tubulin antibody and *β*‐Actin was purchased from Beyotime Biotechnology Co. Ltd. (Shanghai, China). E‐cadherin antibody, Vimentin antibody, and N‐cadherin antibody were purchased from Proteintech Group, Inc. (Whhan, China).

### 2.16. Statistical Analysis

R software 4.1.3 was used for data analysis and visualization. Comparisons between the two groups were evaluated by the Wilcoxon rank‐sum test, while the Kruskal–Wallis test was used to compare more than two groups. Categorical variables were compared using Fisher’s exact test or Chi‐square test. The log‐rank test was used to determine the difference between survival curves. Correlations between two variables were tested with the Spearman correlation test. All *p* values < 0.05 were considered to indicate statistical significance.

## 3. Results

### 3.1. Identification and Exploration of Differentially Expressed TRGs

To explore the impact of TRGs on the outcome of patients with ESCA, our first step in identifying DEGs was to compare ESCA with normal esophageal tissues utilizing the TCGA database (Supplementary Table S3). Through the intersection of ESCA DEGs and genes associated with the telomere, 265 common DEGs were identified as telomere‐related DEGs (Supplementary Table S4). According to the GO annotation, telomere‐related DEGs were found to be significantly enriched in biological processes and molecular functions, such as positive regulation of DNA transcription, negative regulation of transcription from the RNA polymerase II promoter, DNA replication, DNA repair, and the cell cycle. These processes were predominantly located in the nucleus and nucleoplasm, areas rich in telomere content (Figure 2a). Furthermore, the pathways involved in the cell cycle, DNA replication, homologous recombination, mismatch repair, p53 signaling, and nucleotide excision repair were predicted to be associated with a greater concentration of telomere‐related DEGs according to KEGG pathway analysis (Figure 2b).

Figure 2Exploration of telomere‐related DEGs. Gene ontology (GO) functional annotation (a) and KEGG pathway enrichment analysis (b) of telomere‐related DEGs.

**Figure:**
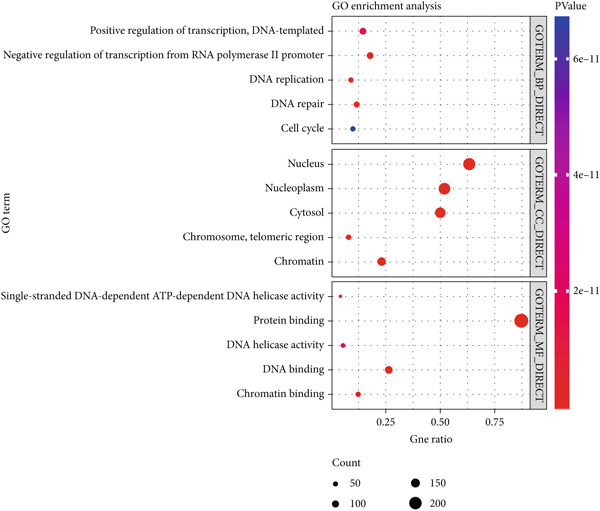
(a)

**Figure:**
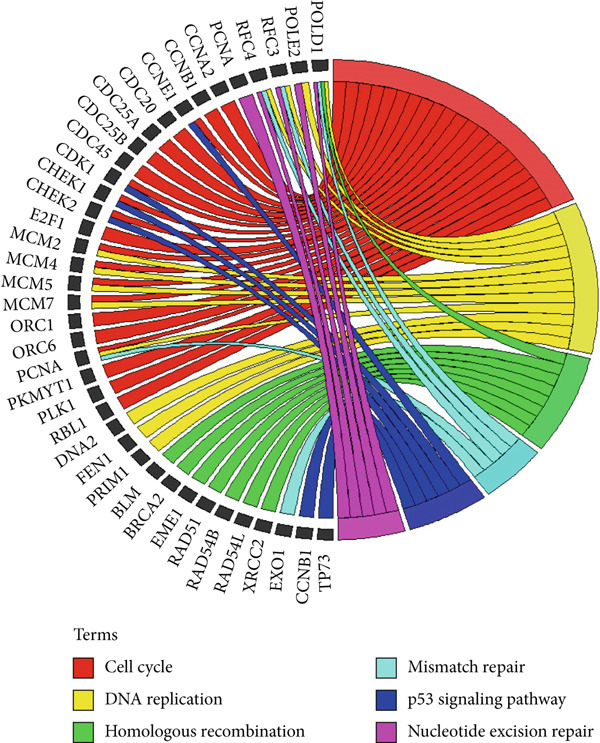
(b)

### 3.2. The TRG Risk Model Could Predict the Prognosis of Patients With ESCA

First, 256 TRGs were detected by Venn diagram intersection (Figure 3a). Among the 265 DEGs, 33 genes were found to be correlated with the overall survival of patients with kidney cancer in the TCGA cohort (Supplementary Table S5). Subsequently, four of these 33 genes were ultimately screened by LASSO regression (Figure 3b,c). Finally, three of these four TRGs (MAPK12, GABRB3, CHAF1B) were identified as independent risk factors through multivariate Cox regression and were used to construct a TRG risk model (Figure 3d). The TRG risk score formula was as follows: risk score = 0.03203866∗CHAF1B + 0.07831127∗MAPK12 − 0.09731058∗GABRB3. According to the median value of the signature, patients with ESCA were stratified into high‐risk (*n* = 83) and low‐risk (*n* = 83) groups. The baseline characteristics of patients with ESCA according to the predictive model are displayed in Table 1.

Figure 3Construction of a prognostic model for patients with ESCA based on telomere ‐related DEGs. (a) The Venn diagram displays the intersection of common genes among ESCA‐related DEGs and telomere‐related genes. (b) The LASSO regression algorithm was used to select the optimal variable (*λ*) with a 10‐fold cross‐validation method. (c) The solution path was plotted according to coefficients against the L1 norm. (d) The forest plot shows the results of hazard ratios and 95% confidence intervals of signature genes from the multivariate Cox regression analysis. (e) The distribution of risk score, survival status, and the expression levels of coefficients in the prognostic signature. (f) The time‐dependent ROC curves for the prognostic signature in the TCGA cohort. (g) The overall survival curves of patients with ESCA with high‐ and low‐risk scores were plotted based on the prognostic signature. (h) Univariate Cox regression forest plot identifies MAPK12 and CHAF1B as risk factors (HR > 1) and GABRB3 as a protective factor (HR < 1). (i) mRNA expression levels of the three signature genes in tumor versus normal tissues from the TCGA cohort.

**Figure:**
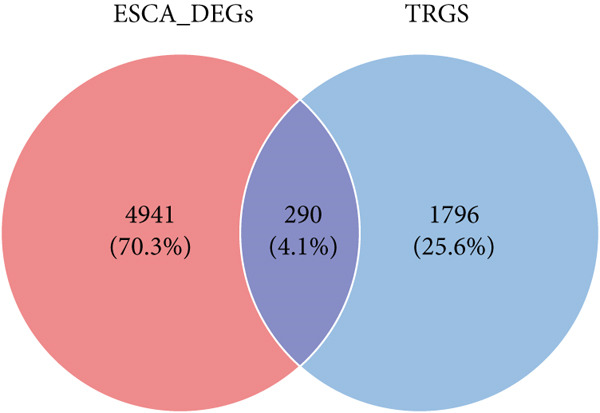
(a)

**Figure:**
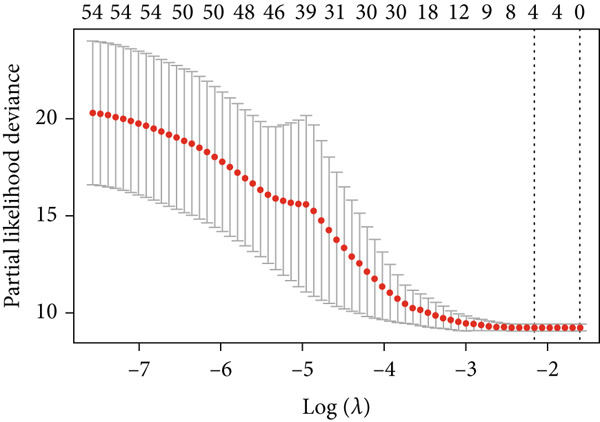
(b)

**Figure:**
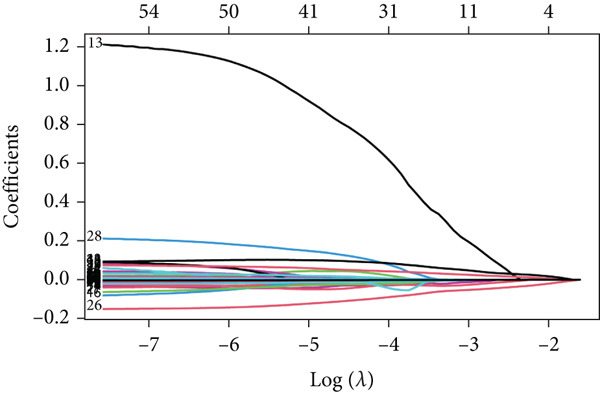
(c)

**Figure:**
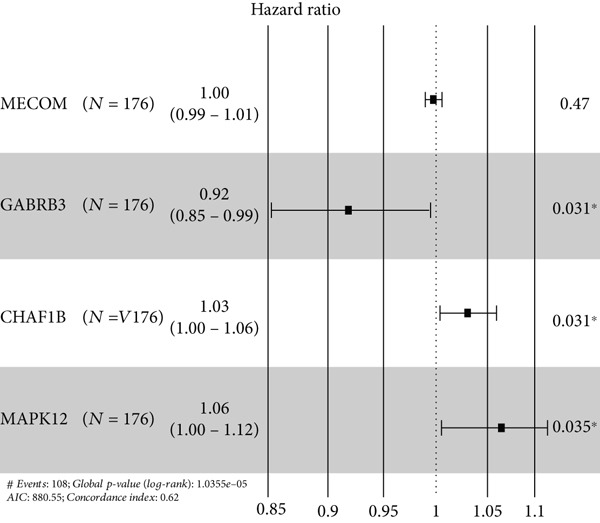
(d)

**Figure:**
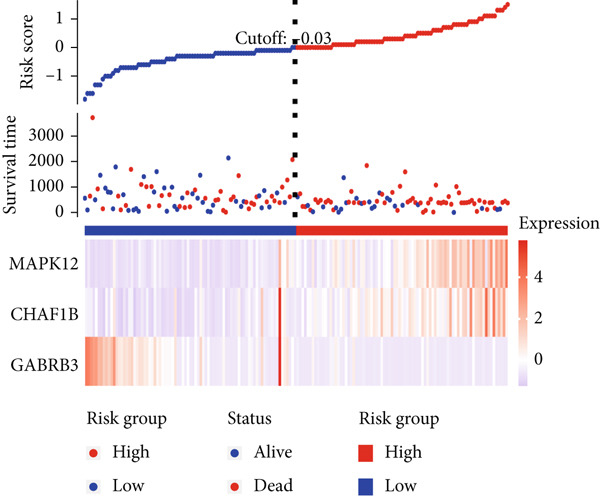
(e)

**Figure:**
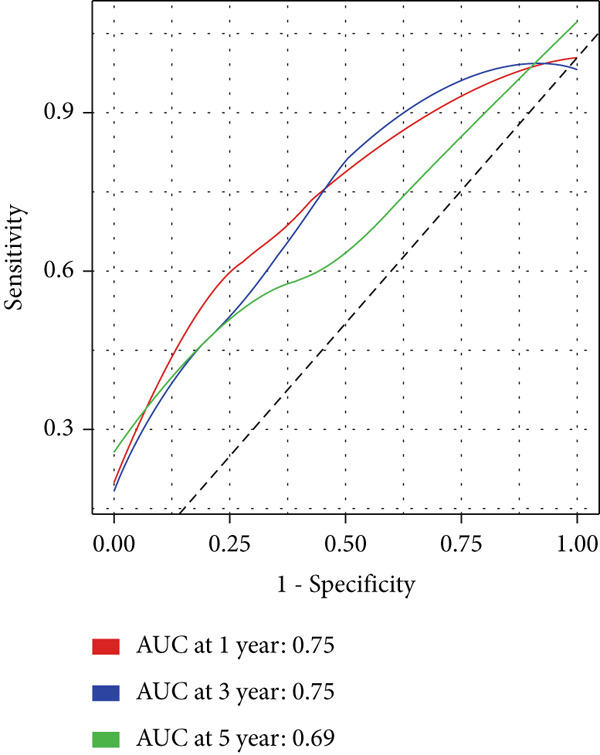
(f)

**Figure:**
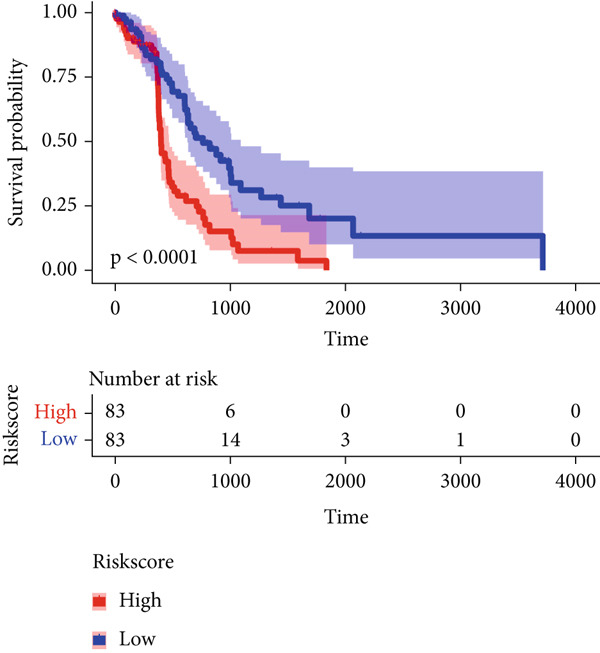
(g)

**Figure:**
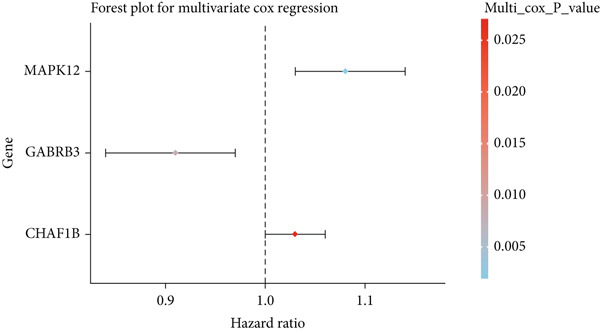
(h)

**Figure:**
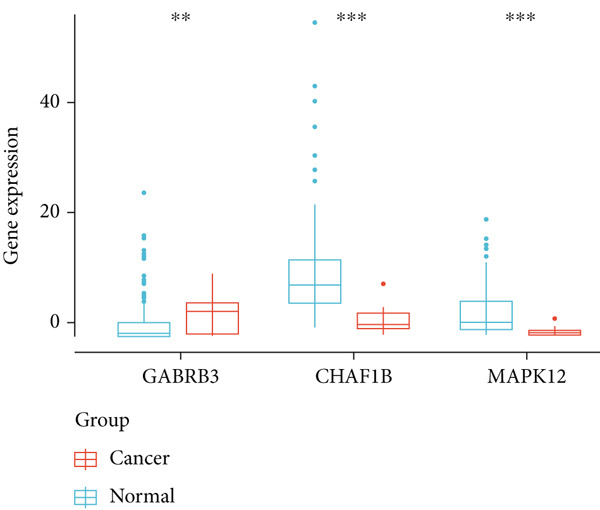
(i)

**Table 1:** Baseline characteristics and comparison of patients with ESCA divided by the prognostic model.

**Characteristic**	**Levels**	**Low-risk**	**High-risk**	**p**	**Method**
*n*		83	83		
Event, *n* (%)	Alive	39	25	0.03817	Chi‐square
	Dead	44	58		
Age, *n* (%)	< 65	38	56	0.007759	Chi‐square
	≥ 65	45	27		
T stage, *n* (%)	T1	30	8	< 0.001	Fisher’s test
	T2	14	27		
	T3	37	45		
	T4	2	3		
N stage, *n* (%)	N0	35	42	0.4695	Fisher’s test
	N1	35	33		
	N2	9	4		
	N3	4	4		
M stage, *n* (%)	M0	78	80	0.7197	Fisher’s test
	M1	5	3		
Stage, *n* (%)	Stage I	17	7	< 0.001	Fisher’s test
	Stage II	31	44		
	Stage III	30	29		
	Stage IV	5	3		
Gender, *n* (%)	Female	18	12	0.3132	Chi‐square
	Male	65	71		

Compared with that in the low‐risk group, the proportion of patients with ESCA who died was significantly greater in the high‐risk group (Figure 3e). To evaluate the predictive accuracy of the prognostic signature, time‐dependent ROC and Kaplan–Meier curves were plotted and compared. The areas under the ROC curves (AUCs) for 1‐, 3‐, and 5‐year OS were 0.75, 0.75, and 0.69, respectively, in the TCGA cohort (Figure 3f). K–M analysis confirmed that patients with ESCA in the low‐risk group had significantly longer OS than did those in the high‐risk group (*p* < 0.0001) (Figure 3g).

By analyzing the forest plot of univariate Cox regression analysis (Figure 3h), we found that two genes (MAPK12 and CHAF1B) involved in the prognostic signature were risk factors (HR > 1), while GABRB3 was a protective factor (HR < 1) for patients with ESCA. To further validate the expression patterns of the signature genes in patients with ESCA, we compared the mRNA expression profiles between tumor tissue and normal tissue in TCGA (Figure 3i). To further confirm the robustness of the prognostic signature, the GEO dataset was screened and used as an external validation cohort. The risk scores of the GEO dataset were calculated, and patients with ESCA were divided into high‐risk and low‐risk groups according to the optimal cutoff value of risk scores. Survival analysis of the validation dataset demonstrated that patients with ESCA in the high‐risk group had significantly poorer overall survival than those in the low‐risk group. The time‐dependent ROC curves also indicated similar results to those of the TCGA training dataset (Supplementary Figure S1).

### 3.3. Correlation Analysis Between the Prognostic Signature and Clinical Characteristics of Patients With ESCA

**Figure 4:**
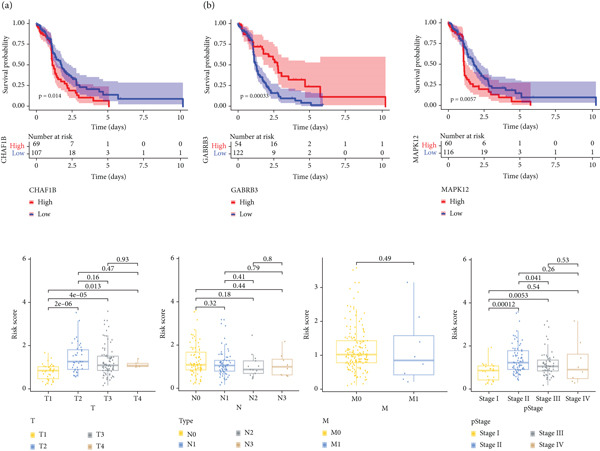
(a) Survival analysis of genes involved in the prognostic signature. (b) Subgroup analysis based on the clinical characteristics of patients with ESCA.

To determine the clinical significance and *p* value of the signature, we first determined the prognostic value of every gene involved in the signature by plotting survival curves. According to the log‐rank test, patients in the high *CHAF1B* and *MAPK12* expression groups had worse overall survival outcomes than did those in the low‐expression group (*p* < 0.05). In contrast, patients with ESCA with high expression of GABRB3 had significantly longer overall survival than those with low expression (*p* < 0.05) (Figure 4a). In addition, clinical feature‐based subgroup analysis indicated that the risk score varied significantly among pathological stages (Stage II and Stage III vs. Stage I, Stage II vs. Stage III, *p* < 0.05) and T stages (T2, T3, and T4 vs. T1, *p* < 0.05), but M stage and N stage did not significantly differ (Figure 4b).

### 3.4. Construction and Evaluation of a Nomogram for Survival Prediction in Patients With ESCA

To establish a prognostic nomogram for predicting the survival of patients with ESCA, clinical features and risk scores were used to carry out univariate and multivariate Cox analyses. Univariate Cox regression indicated that pathological stage and risk score were closely correlated with overall survival in patients with ESCA. Further multivariate analysis confirmed that the risk score was an independent factor affecting the prognosis of patients with ESCA (Table 2).

**Table 2:** The univariate and multivariate Cox regression analyses of clinical characteristics for overall survival in patients with ESCA.

**Characteristic**	**Total(*N*)**	**Univariate analysis hazard ratio (95% CI)**	**p** **value**	**Multivariate analysis hazard ratio (95% CI)**	**p** **value**
Age	166				
< 65	94	Reference			
≥ 65	72	0.98 (0.66–1.46)	0.918	0.8 (0.52–1.22)	0.296
T stage	166				
TI	38	Reference			
TII	41	1.18 (0.66–2.09)	0.577	0.69 (0.3–1.59)	0.382
TIII	82	1.52 (0.92–2.52)	0.102	0.75 (0.28–1.95)	0.549
TIV	5	1.14 (0.26–4.87)	0.864	0.45 (0.06–3.31)	0.437
N stage	166				
N0	77	Reference			
N1	68	0.87 (0.57–1.34)	0.535	0.79 (0.36–1.7)	0.544
N2	13	1.01 (0.4–2.55)	0.975	0.66 (0.18–2.42)	0.533
N3	8	1.47 (0.59–3.71)	0.41	1.22 (0.34–4.37)	0.758
M stage	166				
MO	158	Reference			
M1	8	0.3 (0.04–2.15)	0.23	0.53 (0.06–4.8)	0.576
P stage	166				
I	24	Reference			
II	75	1.22 (0.7–2.11)	0.489	1.6 (0.61–4.18)	0.34
III	59	1.4 (0.77–2.57)	0.272	2.55 (0.51–12.67)	0.252
IV	8	0.37 (0.05–2.79)	0.335	0.53 (0.53–0.53)	< 0.001
Risk score	166				
Low	83	Reference			
High	83	0.43 (0.29–0.65)	< 0.001	0.4 (0.26–0.62)	< 0.001
Gender	166				
Female		Reference			
Male		0.83 (0.52–1.31)	0.418	0.71 (0.43–1.19)	0.192

Subsequently, a nomogram incorporating independent factors was established for predicting 1‐year, 3‐year, and 5‐year OS (Figure 5a). The calibration curves fit well with the ideal diagonal line. These findings indicate good discrimination of the model (Figure 5b). DCA also demonstrated that compared with the TNM stage or prognostic signature, the nomogram model better predicted the 1‐year, 3‐year, and 5‐year OS of patients with ESCA (Figure 5c).

Figure 5(a) A nomogram model was constructed to predict the 1‐year, 3‐year, and 5‐year overall survival of patients with ESCA. (b) Calibration curves of the nomogram model for 1‐year, 2‐year, 3‐year, 4‐year, and 5‐year overall survival. (c) Decision curve analysis for 1‐year, 3‐year, and 5‐year overall survival of the nomogram model.

**Figure:**
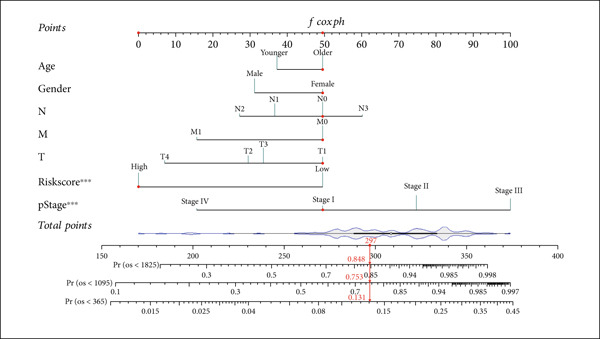
(a)

**Figure:**
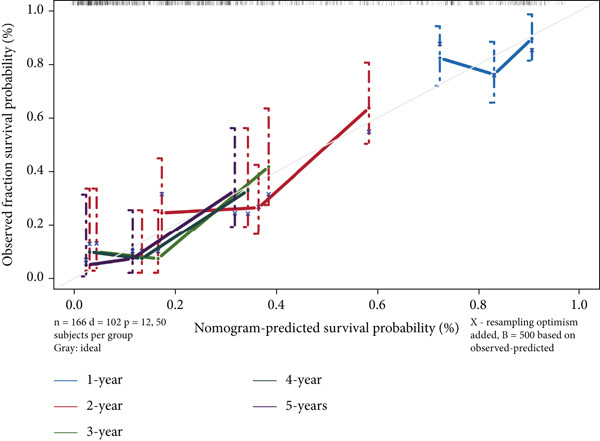
(b)

**Figure:**
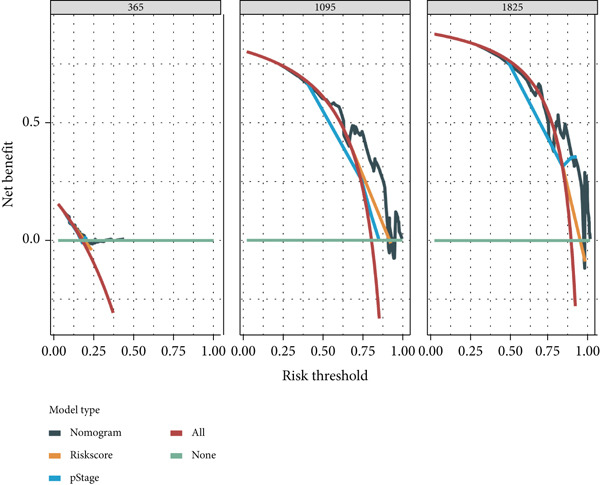
(c)

### 3.5. Mutation Analysis Based on the Prognostic Signature

For mutation analysis of the prognostic signature, waterfall plots were constructed to depict the types and frequencies of somatic mutations within the high‐risk and low‐risk groups. According to these data, the high‐risk group had a significantly greater frequency of mutations, which are the most frequently mutated genes, than did the low‐risk group. The other nine ESCA‐mutated genes (*TTN*, *PIK3CA*, *KMT2D*, *CSMD3*, *MUC16*, *FLG*, *DNAH5*, *NOTCH1*, and *NFE2L2*) also showed various degrees of increasing trends (Figure 6).

Figure 6Comparison of somatic mutation rates between the low‐risk (a) and high‐risk (b) groups in the TCGA cohort.

**Figure:**
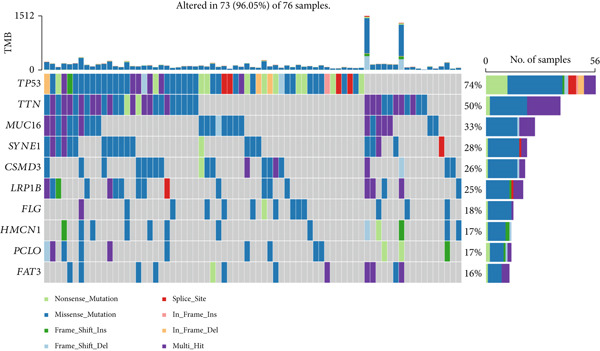
(a)

**Figure:**
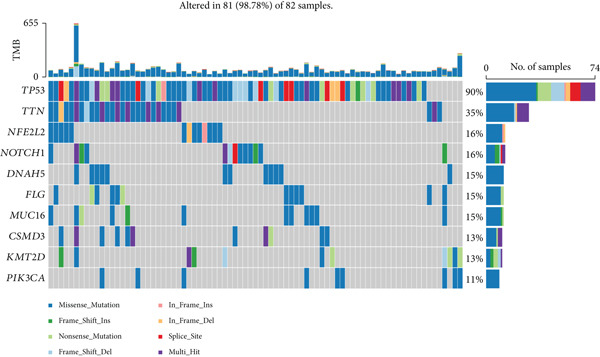
(b)

### 3.6. Functional Enrichment Analysis

To gain a better understanding of the underlying mechanisms behind the survival difference, GSEA was conducted between the high‐risk and low‐risk groups. KEGG analysis revealed that the genes in the high‐risk group were enriched mainly in cancer‐related pathways and metastasis‐related pathways (Figures 7a, 7b, 7c, 7d, 7e, and 7f). GO results revealed that the genes in the high‐risk group were closely associated with the cell cycle. Moreover, the linkage between the GO results and each gene and the linkage between the GO results were investigated (Supplementary Figure S2).

Figure 7The KEGG signaling pathways enriched by GSEA. (a–f) The signaling pathways of cancer and metastasis were mainly enriched in the high‐risk group.

**Figure:**
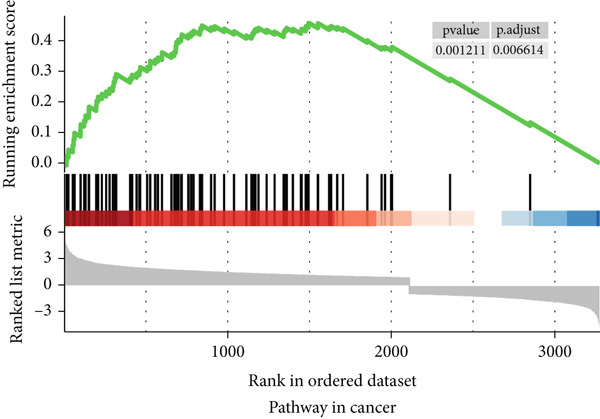
(a)

**Figure:**
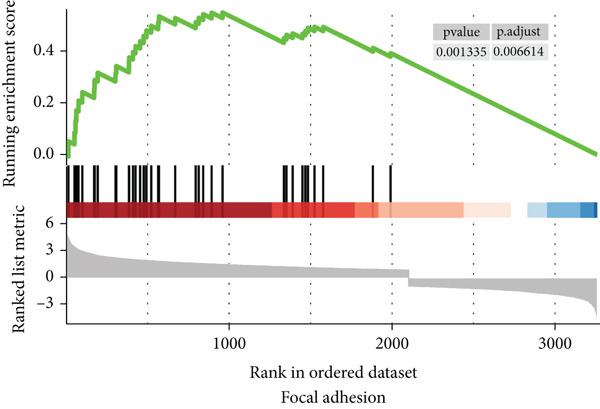
(b)

**Figure:**
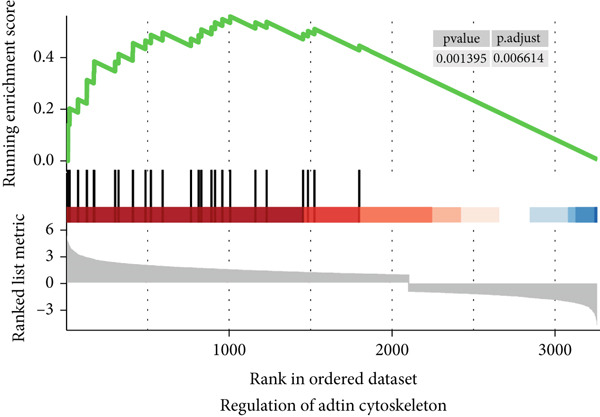
(c)

**Figure:**
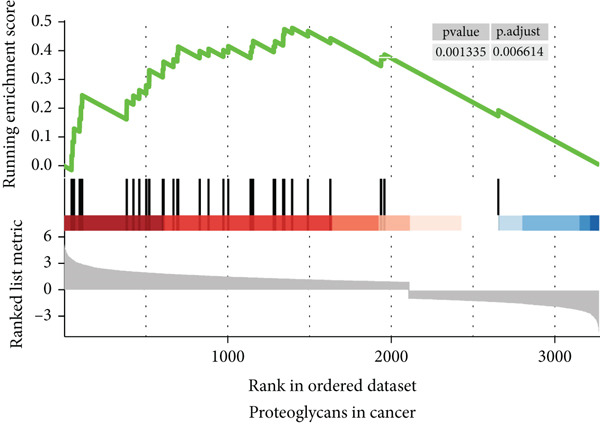
(d)

**Figure:**
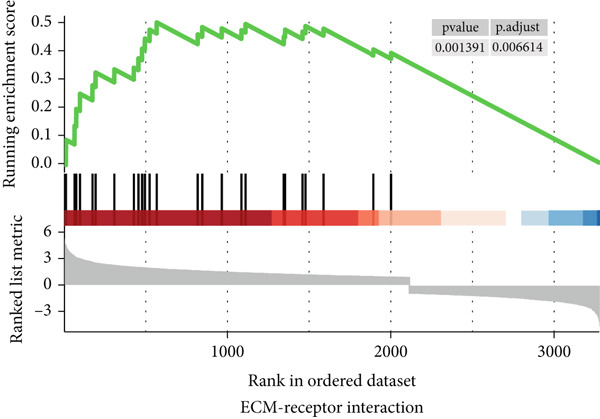
(e)

**Figure:**
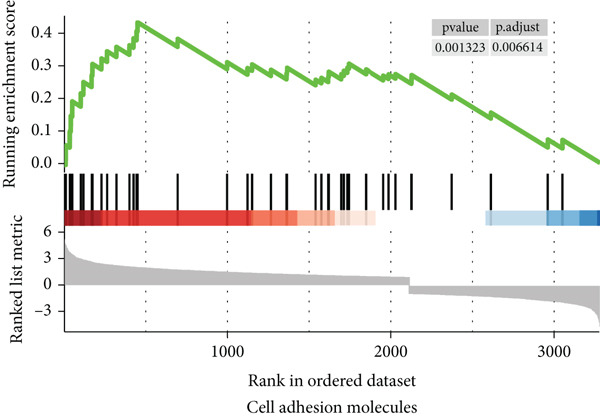
(f)

To distinguish between the biological behaviors of high‐risk and low‐risk patients with ESCA, we performed GSVA. The results demonstrated that pathways associated with tumor progression, such as glycolysis, Myc targets, E2F targets, angiogenesis, the epithelial–mesenchymal transition (EMT), the G2M checkpoint, and the hedgehog signaling pathway, were mainly enriched in the high‐risk group of patients with ESCA (Supplementary Figure S3).

### 3.7. Immune Infiltration Analysis Based on the Prognostic Signature

According to functional enrichment analysis, there was a strong connection between the prognostic signature and immune response gene set variation, which was correlated with the risk score and infiltration of immune cells. Compared with those in the low‐risk group, the TIDE scores and exclusion scores in the high‐risk group were significantly greater, which suggests that immune checkpoint blockade therapy is less effective (Figures 8a, 8b, and 8c). Furthermore, the immune score was greater in the low‐risk group than in the high‐risk group (*p* < 0.001), which indicated that a decreased percentage of immune infiltration was more likely to indicate tumorigenesis (Supplementary Figure S4).

Figure 8Tumor tissues in the high TRGs risk group present the high TIDE and exclusion score (a, b), and the MSI (c) score has no significant difference in these two groups (∗∗∗∗ represents *p* < 0.0001, ns represents no significant difference).

**Figure:**
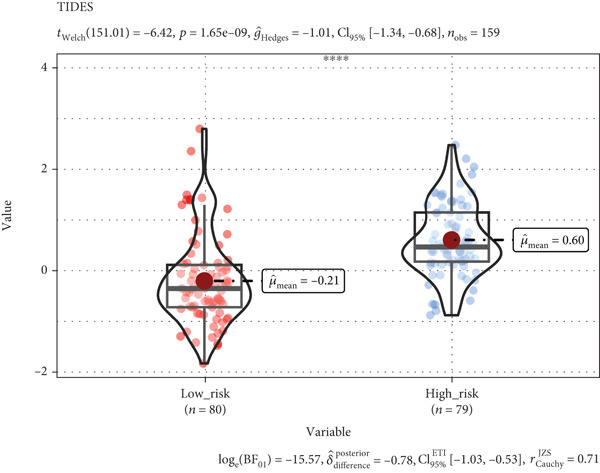
(a)

**Figure:**
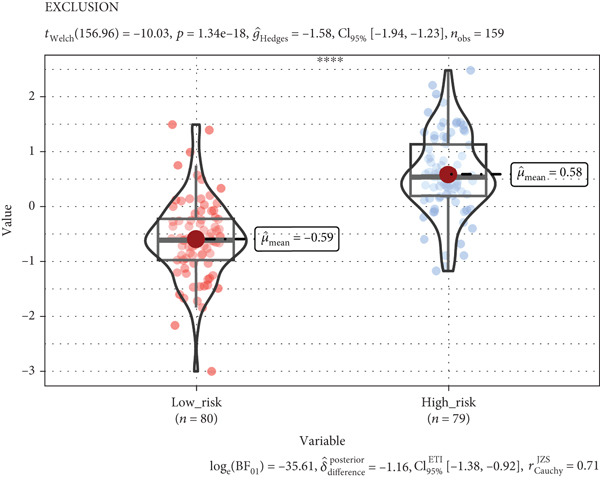
(b)

**Figure:**
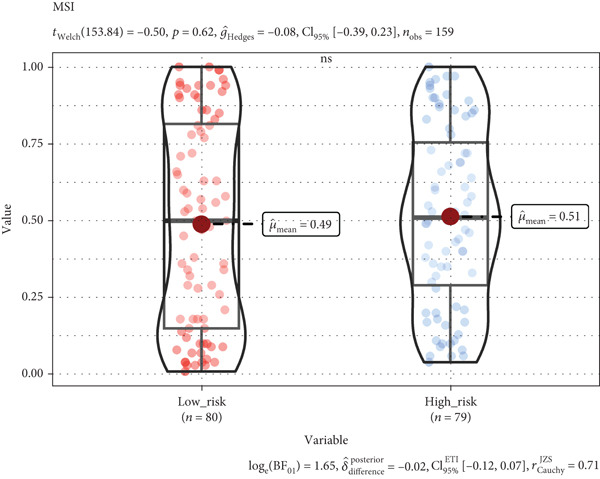
(c)

The tumor microenvironment demonstrated a varying rate of immune cell infiltration among the different tumor types compared with that in the high‐ and low‐risk groups. Activated memory CD4 T cells, M0 macrophages, M2 macrophages, resting dendritic cells, and activated dendritic cells had greater infiltration in the high‐risk group than in the low‐risk group. CD4+ T cells, plasma cells, and Tregs exhibited less infiltration in the high‐risk group than in the low‐risk group (Figure 9a). Previous studies have clustered tumors in the TCGA database into six subtypes according to immune status: C1 (wound healing), C2 (IFN‐g dominant), C3 (inflammatory), C4 (lymphocyte depleted), C5 (immunologically quiet), and C6 (TGF‐b dominant) (21). According to the immune subtype analysis results, patients in the high‐risk group were more likely to have the C1 (2%) and C2 (7%) subtypes than were those in the low‐risk group, and the low‐risk group had fewer patients with the C3 (1%), C4 (5%), and C6 (2%) subtypes than did the high‐risk group (Figure 9b). We utilized the CIBERSORT algorithm to establish correlations between the three prognostic genes and specific immune cells. The results showed that naive B cells, plasma cells, and T‐cell cell regulators (Tregs) were negatively correlated with risk genes (MAPK12 and CHAF1B) and positively correlated with the protective gene GABRB3, while the opposite was true for M0 macrophages (Figure 9c).

Figure 9The immune cells and subtypes of the TRGs risk group. (a) The immune cells distribution portion in the cancer tissues with different TRGs risk group patients. (∗represents *p* < 0.01, ∗∗represents *p* < 0.01, ∗∗∗represents *p* < 0.001). (b) One hundred and forty‐two patients in the total TCGA cohort has the immune subtype results, no C5 subtype in these patients, and C2 is the most common subtype in the two TRGs risk group. (c) The correlations between the three prognostic genes and specific immune cells.

**Figure:**
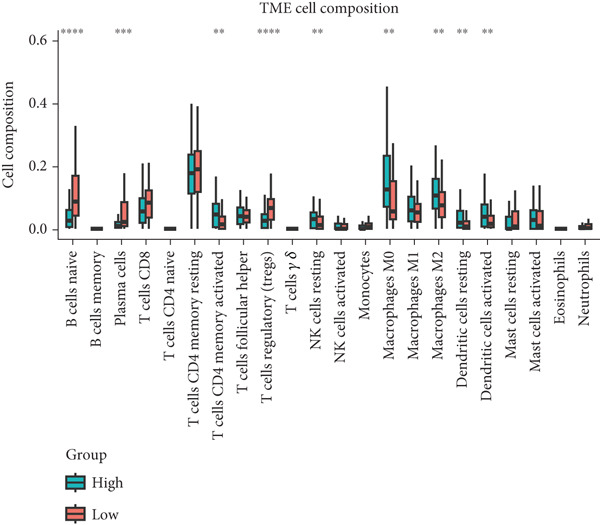
(a)

**Figure:**
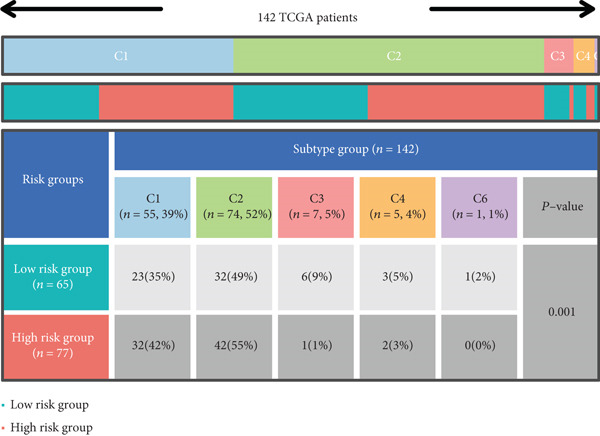
(b)

**Figure:**
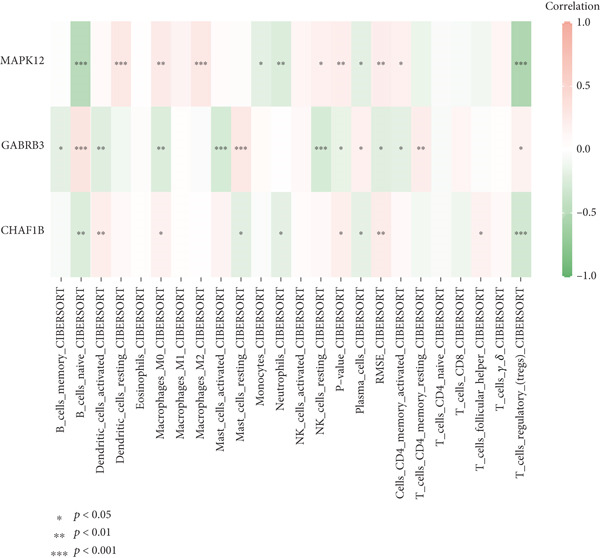
(c)

### 3.8. A TRG Risk Model Could Be Used for Choosing a Treatment Strategy

To elucidate the connection between drug sensitivity and risk scores, we meticulously analyzed the sensitivity of patient with ESCA risk groups to frequently used chemotherapeutics via the GDSC2 database. Our analysis indicated that the estimated IC50 values for six drugs (5‐fluorouracil, erlotinib, gefitinib, docetaxel, paclitaxel, and cisplatin) were significantly lower in the high‐risk group (*p* < 0.05), suggesting that patients in this group might be more responsive to chemotherapy and targeted therapies (Figures 10a, 10b, 10c, 10d, 10e, and 10f).

Figure 10The treatment response of targeted agents, 5‐fluorouracil (a), erlotinib (b), gefitinib (c), docetaxel (d), paclitaxel (e), and cisplatin (f).

**Figure:**
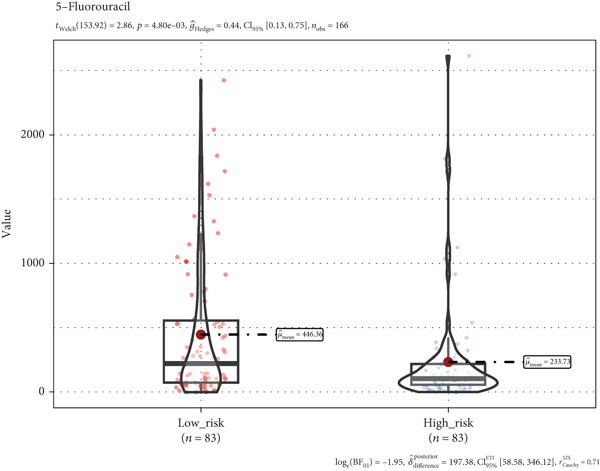
(a)

**Figure:**
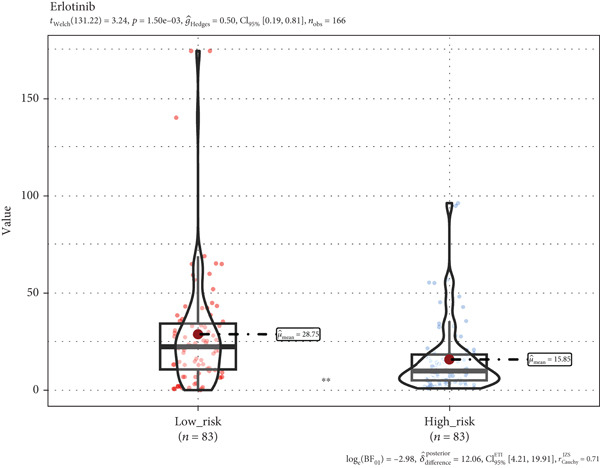
(b)

**Figure:**
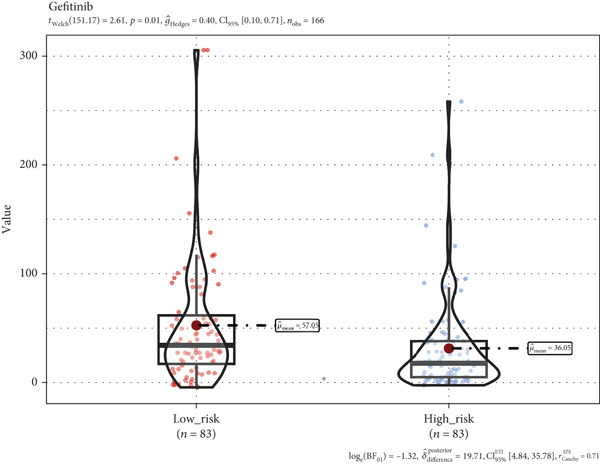
(c)

**Figure:**
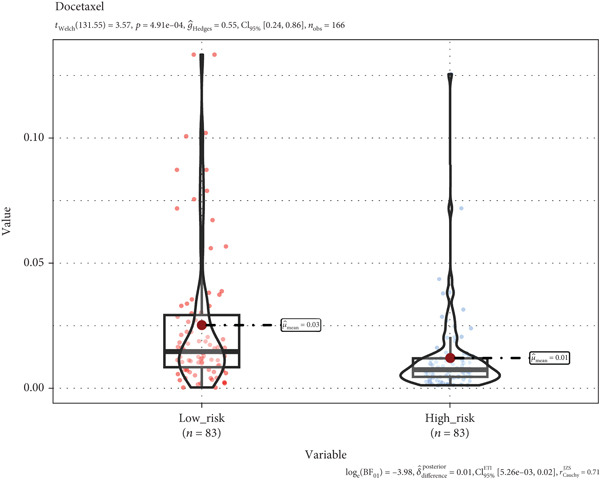
(d)

**Figure:**
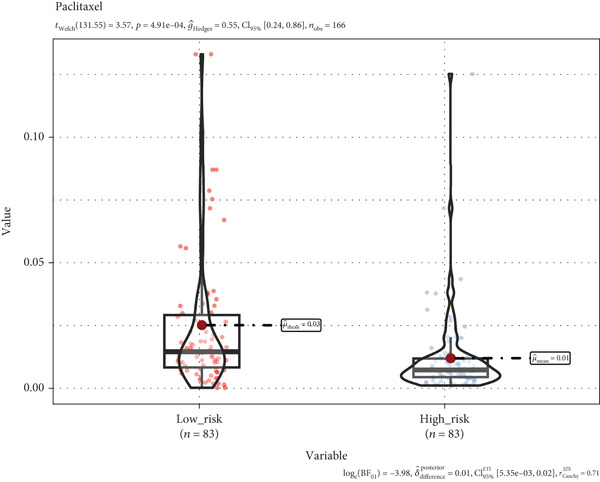
(e)

**Figure:**
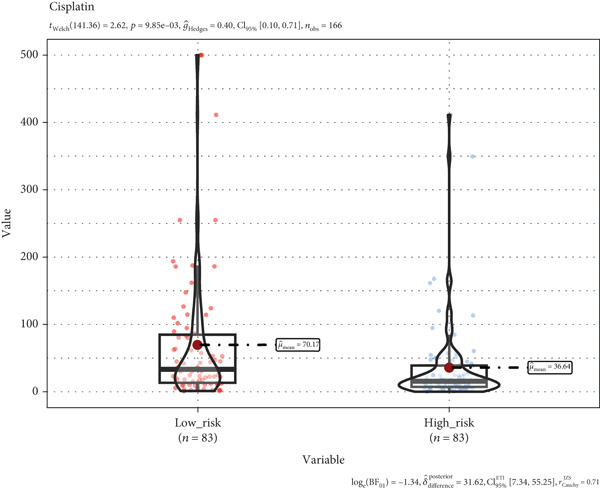
(f)

### 3.9. Construction of the ceRNA Network

Using Version 3 of miRWalk, we conducted an analysis to predict the interactions between MAPK12, GABRB3, and CHAF1B involving miRNAs and found that nine potential regulatory factors existed for these target genes (Figure 11a). To explore their potential interactions with lncRNAs, we utilized ENCORI and discovered that only hsa‐miR‐4756‐5p corresponded to an lncRNA. To gain a comprehensive understanding of their interactions, we constructed an integrated mRNA‐miRNA‐lncRNA regulatory network based on these findings (Figure 11b).

Figure 11Regulatory network of mRNA‐miRNA‐lncRNA. (a) miRNA‐mRNA interactions of MAPK12, GABRB3, and CHAF1B. (b) ceRNA regulatory network of MAPK12, GABRB3, and CHAF1B.

**Figure:**
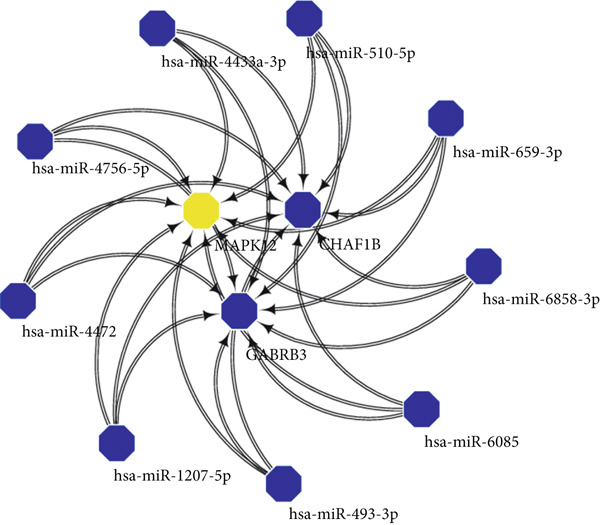
(a)

**Figure:**
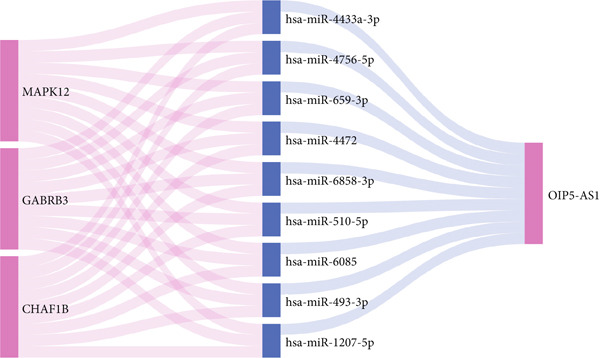
(b)

### 3.10. Downregulated *MAPK12* Inhibits the Proliferation and Migration of Esophageal Carcinoma Cells

To further validate the effectiveness of our prognostic model, we selected the potential oncogene MAPK12, which has not been previously reported in patients with ESCA, and verified its biological function through in vitro experiments. For the loss‐of‐function assay, siRNAs targeting *MAPK12* were transfected into ECA109 and KYSE160 cells to explore whether *MAPK12* exerts effects on ESCA cell function. First, the knockdown efficiency of the siRNAs was verified by Western blotting. Since the knockdown of siRNA‐1 was more effective, we chose siRNA‐1 for subsequent experiments (Figure 12a). In the subsequent wound healing assay, after 48 h of culture, we found that the migration of ECA109 and KYSE160 cells with MAPK12 knockdown to the center was greater than that of cells in the si‐NC group, indicating that siMPK12 had an inhibitory effect on cell migration. Moreover, the results of the transwell assay showed that MAPK12 knockdown significantly inhibited the invasive ability of ECA109 and KYSE160 cells (Figures 12b, 12c, and 12d). Similarly, the proliferative activity of cells in the si‐MAPK12 group was markedly attenuated (Figure 12e,f). In addition, the CCK‐8 assay revealed that knockdown of MAPK12 inhibited cell proliferation (Figure 12g). Therefore, these findings suggested that *MAPK12* could promote the proliferation and migration of ESCA cells, which may contribute to the poor prognosis of patients with ESCA to a certain extent.

Figure 12Downregulation of MAPK12 inhibits the proliferation and migration of ESCA cells. (a) The Western Blotting was performed to validate the transfected efficiency. (b–d) ECA109 and KYSE160 migration ability was analyzed using the wound‐healing assay and transwell migration assay. (e–f) CCK‐8 assay was used to determine the proliferation ability of ECA109 and KYSE160. (g) si‐MAPK12 significantly inhibited colony formation of ECA109 and KYSE160. Data represent mean ± SD from three replicates of each sample. ns, no significance; ∗∗*p* < 0.01; ∗∗∗*p* < 0.001; ∗∗∗∗*p* < 0.0001.

**Figure:**
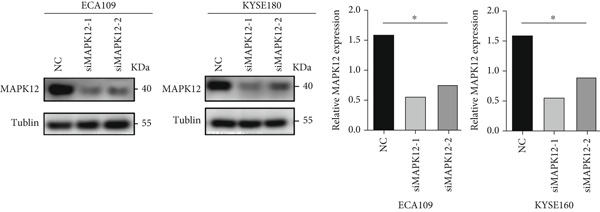
(a)

**Figure:**
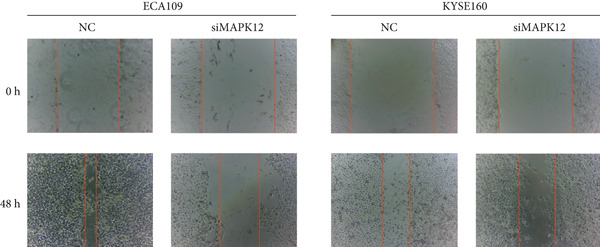
(b)

**Figure:**
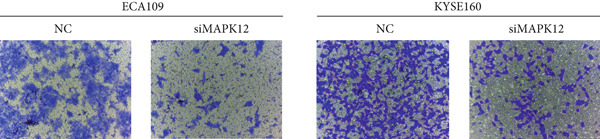
(c)

**Figure:**
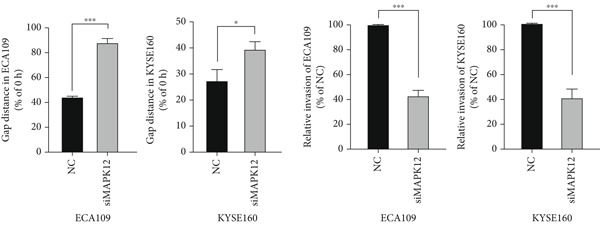
(d)

**Figure:**
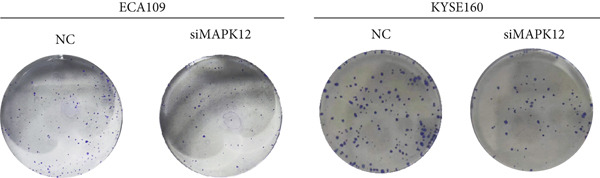
(e)

**Figure:**
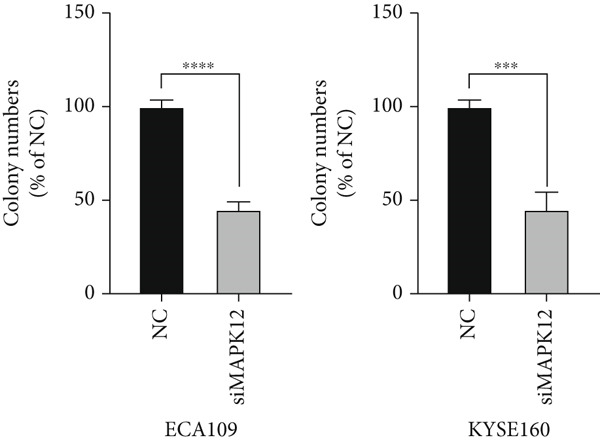
(f)

**Figure:**
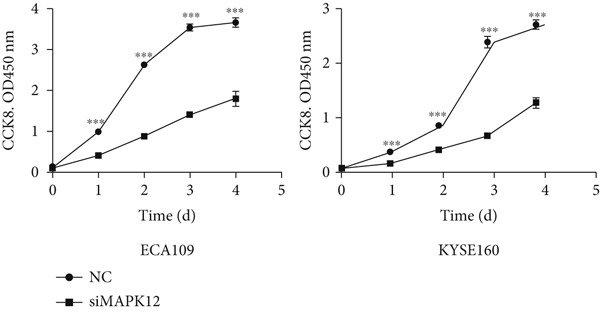
(g)

### 3.11. Inhibition of MAPK12 Reduced EMT in ESCA Cells

As mentioned previously, GSEA indicated that metastasis‐related pathways, such as focal adhesion and cell adhesion molecules, were significantly enriched in the high‐risk group, and thus we hypothesized that TRGS might influence the growth and metastasis of esophageal cancer through EMT. WB results showed that the EMT‐related molecule E‐cadherin was significantly upregulated in the siMAPK12 group compared with the NC group, whereas the opposite was true for Vimentin and N‐cadherin. Therefore, this also suggests that inhibition of MAPK12 may suppress EMT and thus affect the invasion and metastasis of esophageal cancer (Figure 13).

Figure 13MAPK12 regulates EMT in vitro. (a) The effects of si‐MAPK12 on E‐cadherin, Vimentin, and N‐cadherin in ECA109 cells and KYSE160 cells were analyzed by Western blot (*n* = 3). (b) Protein level of E‐cadherin, Vimentin, and N‐cadherin was quantified.

**Figure:**
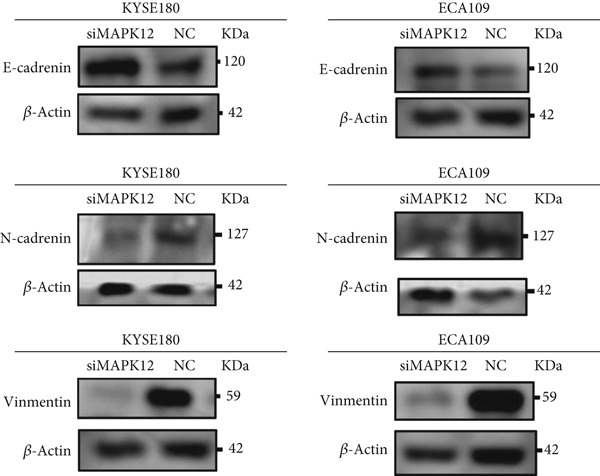
(a)

**Figure:**
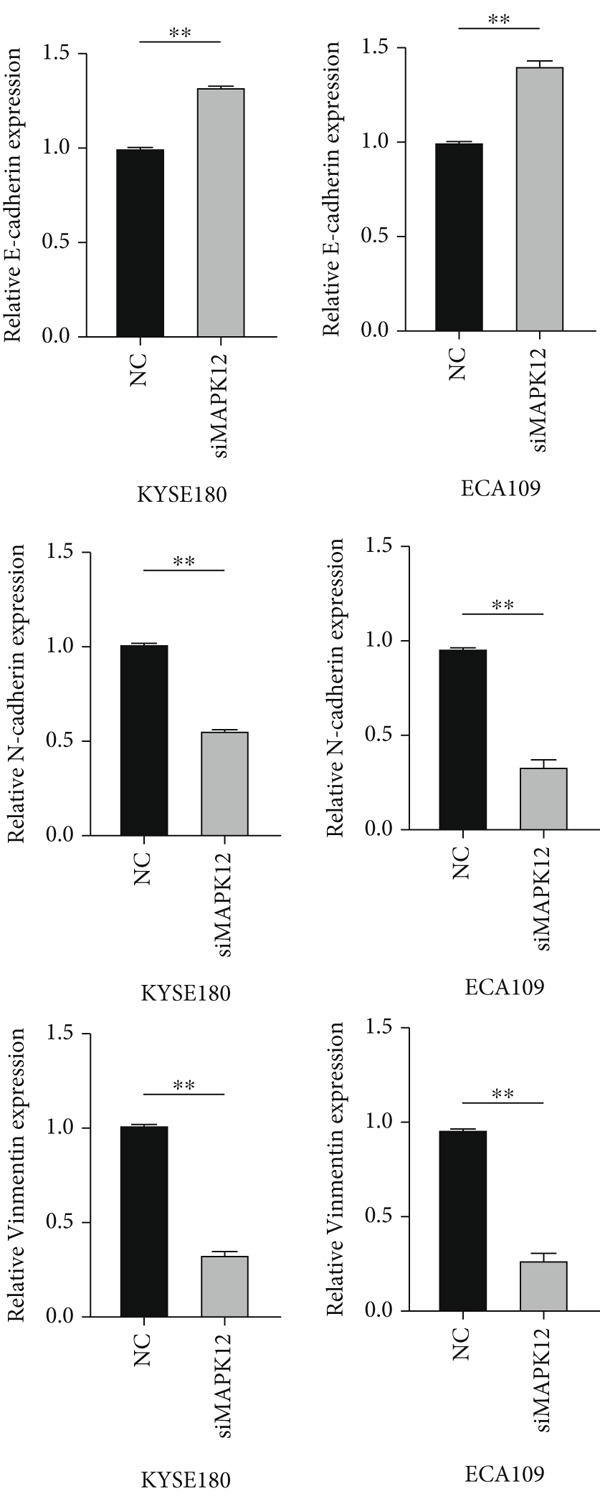
(b)

## 4. Discussion

In 2022, ESCA impacted more than 510,716 people and caused the loss of 445,129 lives worldwide. These numbers are a stark reminder of the urgent need for continued research and improved treatment options to combat this devastating disease [1]. The majority of patients with ESCA presents no significant early symptoms and are often diagnosed at an advanced stage [24, 25]. Telomere plays a key role in the evolution of ESCA, and its characteristics provide a potential intervention target for cancer treatment. Cancer cells mainly achieve unlimited proliferation by activating telomere‐maintenance mechanisms (TMM), such as telomerase or the alternative telomere elongation (ALT) pathway [26]. However, telomere shortening has a dual role: on the one hand, it suppresses tumorigenesis by hindering cell growth and inducing widespread genomic instability. On the other hand, this genomic instability may also promote cancer progression [27, 28]. Thus, investigating telomere‐related molecular markers is crucial for the diagnosis and treatment of advanced ESCA.

First, by intersecting TRGs and DEGs associated with ESCA, we were able to obtain DEGs related to telomeres. Through GO annotation and KEGG enrichment analyses, these DEGs were found to be predominantly involved in DNA‐related biological processes, including cell cycle, DNA replication, homologous recombination, mismatch repair, p53 signaling, and nucleotide excision repair. As the master regulator of its signaling pathway, the tumor suppressor p53 is a DNA‐binding transcription factor central to DNA damage response and cell cycle control [29]. It reported that the activation of the tumor suppressor p53 pathway by DNA damage signals originating from telomere dysfunction serves as a crucial anticancer barrier by inhibiting the proliferation of potential cancer precursor cells [30]. Then, three variables were identified and included in the final prognostic signature by univariate, LASSO regression, and multivariate Cox analyses. Notably, MAPK12 and CHAF1B were positively related to the risk score and overall survival, while GABRB3 was not correlated with these variables. K–M survival curves further demonstrated that patients with ESCA in the high‐risk subgroup experienced significantly shorter OS than did those in the low‐risk subgroup (*p* < 0.001). The time‐dependent ROC curve analysis revealed that the prognostic signature was highly accurate in predicting the survival of patients with ESCA. Additionally, the robustness of this prognostic signature was validated by high‐accuracy GEO datasets.

Clinical feature‐based subgroup analyses revealed a strong correlation between risk score and disease stage. Additionally, univariate and multivariate analyses validated the prognostic value of the signature for patients with ESCA. Then, the C‐index and calibration curves were used to evaluate a nomogram model that was built based on this signature and to determine individualized prognostic scores. The clinical application value of the nomogram model was revealed through the use of a decision nomogram, providing further proof of its reliability.

To conduct a more in‐depth examination of the potential mechanisms that influence survival differences, our initial investigation involved comparing genes that exhibit high rates of somatic mutations in esophageal cancers, such as TP53, TTN, and MUC16. The results showed that the risk scores predicted by the prognostic signature were strongly correlated with the mutation rate of TP53, which could be a contributing factor to the poorer prognosis of patients with ESCA in the high‐risk group. GSEA and GSVA revealed that the high‐risk group was more likely to have pathways related to cancer and metastasis. These findings indicated that the survival differences may be driven by the metastasis of patients with ESCA.

The progression of ESCA is governed by a complex immune landscape, marked by inherent antigenicity countered by adaptive immunosuppression, which underpins both disease pathogenesis and therapeutic opportunities [31].To further elucidate the underlying immune‐related mechanisms of the signature for predicting the prognosis of patients with ESCA, the CIBRESORT algorithm was applied to calculate immune cell infiltration and immune function. We further confirmed that activated memory CD4 T cells, M0 macrophages, M2 macrophages, resting dendritic cells, and activated dendritic cells had greater infiltration in the high‐risk group than in the low‐risk group. Of particular interest is the enrichment of M0 and M2 macrophages, which resonates with a seminal study demonstrating that radiotherapy induces a distinct, PD‐L1+ tumor‐associated myeloid subset that co‐upregulates protumorigenic factors (IL‐6, CXCL8) and immune checkpoints. This convergence of evidence strongly implies that the macrophages identified in our high‐risk cohort may be key drivers of immunosuppression and therapy resistance, highlighting them as a prime target for strategies combining radiotherapy with PD‐L1 blockade or other myeloid‐targeting agents [32]. CD4+ T cells, plasma cells, and Tregs showed less infiltration in the high‐risk group than in the low‐risk group. Recent studies have shown that higher CD4+ + CD8+ T cell combinatorial density in the tertiary lymphoid structure of ESCC is a better prognostic indicator [33]. This convergence of evidence highlights the critical role of CD4+ T cells in particular in the adaptive immune response against esophageal cancer, which is consistent with our work. Previous studies have shown the varied prognoses of tumors with different immune subtypes. The C3 subtype had the most favorable outcome, while the C6 and C4 subtypes had the worst prognosis. Our results suggested that the TRG risk model group was significantly correlated with the immune subtypes. The high‐risk group had a greater proportion of C1 and C2 subtypes and a lower proportion of C3, C4, and C6 subtypes than did the low‐risk group. The immune subtype analysis indicated that the patients in the high‐risk group might have had a poor prognosis because of the greater proportion of subtypes (C1 + C2, 97%) with a worse outcome than in the low‐risk group (84%). In addition, the high‐risk group had a high TIDE score, indicating that the high‐risk group of patients may be more likely to experience immune escape. Overall, the results suggest that the differences in prognosis between the high‐ and low‐risk groups might be partly due to the different immune statuses of the patients. In addition, the high‐risk group of patients with ESCA demonstrated an improved response to chemotherapy, which strongly suggested a correlation with the heightened proliferative and invasive capabilities of the tumors. This remarkable prognostic signature not only exhibits exceptional accuracy and robustness in predicting clinical outcomes but also has enormous potential for estimating the efficacy of chemotherapy and immunotherapy. This signature could be a game changer in the field of medicine, providing healthcare providers with invaluable insights and helping them make informed decisions.

P38 mitogen‐activated protein kinase (MAPK12) (also known as P38 *γ*) [34] has been reported to be expressed in multiple tissues and to promote tumorigenesis and tumor progression [35]. A recent study revealed that high MAPK12 expression promoted EMT in breast cancer cells, and the downregulation of MAPK12 inhibited EMT [36, 37]. CHAF1B, which is located in the chromosome, is the p60 subunit of CAF‐1 and the central factor of chromatin assembly after DNA repair and synthesis [38]. Previous studies have shown that elevated CHAF1B levels are closely associated with poor prognosis in patients with many different types of cancer, such as melanoma [39], prostate cancer [40], salivary gland tumors [41], nasopharyngeal carcinoma [42], and high‐grade glioma [43, 44]. Moreover, researchers have recently reported that CHAF1B is associated with cell proliferation, cell apoptosis, and cell cycle arrest. Many studies have linked this phenomenon to cisplatin resistance [45, 46]. However, the exact mechanisms by which MAPK12, CHAF1B, and GABRB3 are involved in the progression of esophageal cancer have not yet been reported in the literature and warrant further exploration. For further validation, we explored the role of MAPK12 in the progression of esophageal cancer through in vitro experiments. The results demonstrated that the downregulation of MAPK12 could attenuate the proliferation and migration of ESCA cells, which is in agreement with previous bioinformatic predictions. More and more evidence suggest that EMT plays a crucial role in the development of ESCA [41, 42]. We found that inhibiting MAPK12 reduces EMT. Our study identifies MAPK12 as a novel and independent prognostic biomarker in ESCA, adding to the existing landscape of molecular predictors for this disease. While several biomarkers, such as TP53 mutations and high VEGF expression, have been established as indicators of aggressive ESCA, they often relate to general tumor proliferation or angiogenesis [47, 48]. In contrast, the association of MAPK12—a gene encoding a stress‐responsive kinase—with poor prognosis suggests a potential role in tumor migration, invasion, and perhaps treatment resistance, mechanisms that are distinct from those of canonical biomarkers [49]. This distinction is also supported by our functional experiments, which demonstrated that MAPK12 knockdown significantly impaired ESCA cell migration. Furthermore, although MAPK12’s role in ESCA is largely unexplored, our findings align with its reported oncogenic functions in other malignancies, such as liver cancer and diffuse large B‐cell lymphoma, where it promotes cell motility and survival [50, 51]. The identification of MAPK12, in particular, advances our understanding of ESCA pathogenesis by linking telomere biology with MAPK signaling—a pathway implicated in cell survival and migration but not yet fully explored in ESCA prognostication [52]. This finding not only provides a potential mechanistic explanation for the aggressive phenotype observed in high‐risk patients but also opens avenues for targeted intervention, as MAPK12 may represent a druggable node.

Ultimately, our study contributes to the broader goal of personalized oncology in ESCA. The prognostic model offers a tangible step towards refining risk assessment, potentially guiding more individualized surveillance schedules and adjuvant therapy decisions. Future validation in prospective clinical cohorts and functional investigations into the role of MAPK12 will be essential to translate these findings into improved patient outcomes.

While our study presents a novel prognostic signature, several limitations should be acknowledged. First, the model was developed and validated exclusively using retrospective data from public databases (TCGA, GEO). Although we employed rigorous bioinformatics methods, these datasets are subject to potential biases inherent in their original designs, such as variations in patient selection, treatment protocols, and sequencing platforms. The recognition and clinical applicability of our findings therefore require confirmation through large‐scale, multicenter, prospective cohort studies. Secondly, although we preliminarily validated the role of MAPK12 in vitro, the exact mechanism of MAPK12 and other signature genes (such as CHAF1B, GABRB3) in the pathogenesis of ESCA has still not been fully elucidated. Future work should include detailed mechanistic studies such as identifying key downstream effectors of MAPK12 in ESCA, its interaction with telomere maintenance pathways, and its role in mediating chemoresistance, ideally using patient‐derived organoids or genetically engineered mouse models to better mimic the tumor microenvironment. Finally, the clinical applicability of the signature is worth exploring. Future efforts should focus on developing standardized clinical tests based on signature genes (e.g., RT‐PCR or immunohistochemistry) and designing prospective trials to evaluate their utility in guiding adjuvant treatment decisions. These studies may provide a rationale for using this biomarker as a personalized diagnostic and therapeutic option for patients with ESCA.

## 5. Conclusion

In brief, we first developed and validated a new signature that incorporates genes related to telomeres in patients with ESCA. Our study explored the potential clinical significance of this biomarker. Our prognostic model is capable of independently predicting the survival of patients with ESCA, as demonstrated by high‐throughput data mining. The theoretical basis provided by these results can be used to further explore the molecular pathogenesis of ESCA and identify therapeutic approaches. Lastly, the underlying mechanisms need to be further explored and further in vivo experiments are required in order to refine our research.

## Conflicts of Interest

The authors declare no conflicts of interest.

## Funding

No funding was received for this manuscript.

## Supporting information


**Supporting Information** Additional supporting information can be found online in the Supporting Information section.. Supplementary Figure S1: Validation of the prognostic signature model based on the GEO database. Supplementary Figure S2: The linkage between the GO results and each gene and the linkage between the GO results. Supplementary Figure S3: Bar chart (a) and heat map (b) displaying the difference in pathway activities enriched by GSVA between the high‐risk and low‐risk groups. Supplementary Figure S4: The estimate score between the high‐risk and low‐risk groups. Supplementary Figure S5: Full uncropped Gels and Blots images of MAPK12. Supplementary Figure S6: Full uncropped Gels and Blots images of E‐cadrenin. Supplementary Figure S7: Full uncropped Gels and Blots images of N‐cadrenin. Supplementary Table S1: Clinical characteristics of patients with ESCA in the training datasets (TCGA) and the validation datasets (GEO). Supplementary Table S2: List of Tolement‐related genes included in the present study. Supplementary Table S3: List of differentially expressed genes (DEGs) between ESCA and normal lung tissues based on the TCGA database. Supplementary Table S4: List of 265 Tolement‐related DEGs by taking the intersection of DEGs of ESCA and Tolement‐related genes. Supplementary Table S5: 33 candidate genes with prognostic values were screened out by the Kaplan–Meier survival analysis. Supplementary Appendix 1. The detailed protocols for cell culture, transfection, and functional assays. Supplementary Appendix 2: The detailed statistical methods.

## Data Availability

The data that support the findings of this study are available on request from the corresponding author.
