# Supplementary material for: Identification of MAPK12 as a Prognostic Biomarker for Esophageal Carcinoma Using Bioinformatics and Machine Learning
Source: Biomed Res Int. 2025 Dec 29;2025:2605071. doi: 10.1155/bmri/2605071 (PMC12746012; doi:10.1155/bmri/2605071)
Supplement: Supplementary file 1 — Supporting Information Additional supporting information can be found online in the Supporting Information section.. Supplementary Figure S1: Validation of the prognostic signature model based on the GEO database. Supplementary Figure S2: The linkage between the GO results and each gene and the linkage between the GO results. Supplementary Figure S3: Bar chart (a) and heat map (b) displaying the difference in pathway activities enriched by GSVA between the high‐risk and low‐risk groups. Supplementary Figure S4: The estimate score between the high‐risk and low‐risk groups. Supplementary Figure S5: Full uncropped Gels and Blots images of MAPK12. Supplementary Figure S6: Full uncropped Gels and Blots images of E‐cadrenin. Supplementary Figure S7: Full uncropped Gels and Blots images of N‐cadrenin. Supplementary Table S1: Clinical characteristics of patients with ESCA in the training datasets (TCGA) and the validation datasets (GEO). Supplementary Table S2: List of Tolement‐related genes included in the present study. Supplementary Table S3: List of differentially expressed genes (DEGs) between ESCA and normal lung tissues based on the TCGA database. Supplementary Table S4: List of 265 Tolement‐related DEGs by taking the intersection of DEGs of ESCA and Tolement‐related genes. Supplementary Table S5: 33 candidate genes with prognostic values were screened out by the Kaplan–Meier survival analysis. Supplementary Appendix 1. The detailed protocols for cell culture, transfection, and functional assays. Supplementary Appendix 2: The detailed statistical methods. [file BMRI-2025-2605071-s001.zip › Supplementary Appendix 1.docx]

1. Cell Culture

1.1. Thawing and Maintenance of ESCA Cell Lines

Cell Lines: Human esophageal carcinoma cell lines The ECA109 and KYSE160.

Culture Medium: RPMI-1640 , supplemented with 10% fetal bovine serum (FBS) and 1% penicillin-streptomycin. All cells are maintained in a humidified incubator at 37°C with 5% CO₂.

Thawing: Rapidly thaw frozen vials in a 37°C water bath. Transfer the cell suspension to a centrifuge tube containing pre-warmed complete medium. Centrifuge at 1000 rpm for 5 minutes. Aspirate the supernatant, resuspend the cell pellet in fresh complete medium, and seed into a 10cm dish.

Subculturing: When cells reach 80-90% confluence, aspirate the old medium. Rinse the cell monolayer with 1× PBS. Add an appropriate volume of 0.25% trypsin-EDTA solution and incubate at 37°C for 1-2 minutes. Neutralize trypsinization with complete medium. Centrifuge the cell suspension, aspirate the supernatant, and reseed the cells at a desired ratio (typically 1:3 to 1:5) into new dishs.

1.2. Cell Authentication and Mycoplasma Testing

All cell lines were authenticated by short tandem repeat (STR) profiling within the last 6 months.

Cells are routinely tested for mycoplasma contamination using a PCR-based detection kit and are confirmed to be negative.

2. siRNA Transfection for MAPK12 Knockdown

2.1. Transfection Protocol

siRNA: A pool of three target-specific Small interfering RNAs (siRNA) against human MAPK12 and a non-targeting scrambled negative control siRNA (siNC) are used. Small interfering RNAs (siRNA) targeting human MAPK12 and negative control siRNA (siNC) were purchased from Tsingke Biological Technology

Day 0: Seeding. Trypsinize and count cells. Seed cells in antibiotic-free complete medium into 6-well at a density that will reach 30-50% confluence at the time of transfection.

Day 1: Transfection.

Dilution: For each well of a 6-well plate, dilute 5 μL of Lipo8000 reagent in 125 μL of Opti-MEM® Reduced Serum Medium. In a separate tube, dilute 100 pmol of siRNA in 125 μL of Opti-MEM®. Incubate for 30 minutes at room temperature.

Complexation: Combine the diluted Lipo8000 reagent with the diluted siRNA mixture. Mix gently and incubate for 15-20 minutes at room temperature to allow Lipo8000-siRNA complex formation.

Addition: Add the 250 μL complex dropwise onto the cells in the well. Gently rock the plate to ensure even distribution.

Day 2: Medium Change. 6-8 hours post-transfection, replace the transfection mixture with fresh complete medium to reduce cytotoxicity.

Day 3: Harvesting. Cells are harvested 48-72 hours post-transfection for subsequent functional assays or RNA/protein extraction to verify knockdown efficiency via Western blot.

3. Functional Assays

3.1. Cell proliferation assay

1. Seeding: 24 hours post-transfection, seed transfected cells into a 96-well plate at a density of 3×10³ cells per well in 100 μL of medium. Set up 3 replicate wells per group and include blank control wells (medium only).

2. Incubation and Measurement: Incubate the plate for 0, 24, 48, 72 and 96 hours. At each time point, add 10 μL of CCK-8 solution to each well. Incubate the plate at 37°C for 2 hours.

3. Absorbance Reading: Measure the absorbance at 450 nm using a microplate reader. The cell viability is calculated as: (OD_experiment - OD_blank) / (OD_NC -OD_blank) × 100%. The experiment is repeated three times independently.

3.2. Wound Healing (Migration) Assay

1. Scratching: 48 hours post-transfection, seed cells in a 6-well plate to create a confluent monolayer. Carefully scratch the monolayer with a sterile 200 μL pipette tip in a straight line.

2. Washing and Imaging: Gently wash the wells with PBS to remove detached cells. Add serum-free medium. Capture images (0-hour time point) under an inverted microscope at designated locations.

3. Incubation and Re-imaging: Incubate the cells for 24 hours. Capture images at the same locations. The migration rate is quantified by measuring the change in the scratch width using ImageJ software.

3.3. Transwell Migration Assay

1. Preparation: 48 hours post-transfection, harvest the cells (e.g., KYSE-150) by trypsinization. Centrifuge and resuspend the cell pellet in serum-free medium. Count the cells and adjust the density to 5×10⁵ cells/mL.

2. Seeding: Load 200 μL of the cell suspension (containing ~1×10⁵ cells) into the upper chamber of a 24-well Transwell insert (8 μm pore size, Corning). Add 600 μL of complete medium (containing 10% FBS) to the lower chamber as a chemoattractant.

3. Incubation and Staining: Incubate the plate for 24 hours at 37°C in a 5% CO₂ incubator. Carefully remove the insert and use a cotton swab to gently wipe off the non-migrated cells from the upper surface of the membrane. Fix the cells on the lower surface by immersing the insert in 4% paraformaldehyde for 20 minutes. Stain the cells with 0.1% crystal violet solution for 15 minutes. Rinse gently with PBS.

4. Imaging and Quantification: Capture images of five random fields per membrane under an inverted microscope. Count the number of stained cells per field using ImageJ software. The experiment is performed in triplicate and repeated three times independently.

3.4. Colony Formation Assay

1. Seeding: 48 hours post-transfection, harvest the cells. Seed them into 6-well plates at a very low density (500 cells per well) in complete medium to ensure well-isolated colonies. Gently rock the plates to ensure even distribution.

2. Incubation and Maintenance. Incubate the plates for 10 days, allowing colonies to form. Refresh the culture medium every 3 days.

3. Fixing and Staining: Once visible colonies (typically >50 cells) have formed, aspirate the medium. Gently rinse the wells with 1× PBS. Fix the colonies with 4% paraformaldehyde for 20 minutes. Stain the colonies with 0.1% crystal violet solution for 15 minutes. Rinse gently with tap water and air-dry.

4. Imaging and Quantification: Capture images of the entire well. Count the number of colonies manually or using ImageJ software. The colony formation rate is calculated as: (Number of colonies / Number of cells seeded) × 100%. The experiment is repeated three times independently.

4. Western Blot Analysis

4.1. Protein Extraction and Quantification: 72 hours post-transfection, harvest cells using RIPA lysis buffer supplemented with 1% PMSF and a phosphatase inhibitor cocktail. Incubate on ice for 30 minutes with vortexing every 10 minutes. Centrifuge at 12,000 rpm for 15 minutes at 4°C. Collect the supernatant and determine the protein concentration using a BCA protein assay kit.

4.2. Gel Electrophoresis and Transfer: Total protein (20 μg per sample) was mixed with 5× SDS-PAGE loading buffer, denatured at 95°C for 10 minutes, and then loaded onto a 10% SDS-polyacrylamide gel. Electrophoresis was performed initially at 80 V for approximately 30 minutes until the samples condensed and passed the stacking gel, followed by 120 V for 60-90 minutes in running buffer (25 mM Tris, 192 mM glycine, 0.1% SDS, pH 8.3) until the dye front reached the bottom of the gel. Subsequently, the separated proteins were transferred onto a PVDF membrane activated with methanol. The transfer was conducted using a wet transfer system in ice-cold transfer buffer (25 mM Tris, 192 mM glycine, 20% methanol) at a constant current of 300 mA for 90-120 minutes, depending on the molecular weight of the target proteins.

4.3. Blocking and Antibody Incubation: Block the membrane with 5% non-fat milk in TBST for 1 hour at room temperature. Incubate the membrane with specific primary antibodies diluted in 5% TBST overnight at 4°C with gentle shaking.

Primary Antibodies and Dilutions:

Anti-MAPK12 (1:1000)

Anti-E-cadherin (1:1000)

Anti-N-cadherin (1:1000)

Anti-Vimentin (1:1000)

Anti-β-Actin (1:5000)

Anti-β-Tubulin (1:5000)

4.4. Detection: After washing with TBST, incubate the membrane with goat anti-rabbit IgG with dilution ratio 1:500 for 1 hour at room temperature. Wash the membrane thoroughly. Detect the protein bands using an enhanced chemiluminescence (ECL) substrate kit and visualize with a chemiluminescence imaging system.

4.5. Analysis: Quantify the band intensity using ImageJ software. The expression level of each target protein is normalized to the intensity of the loading control (β-Actin and β-Tubulin).
