# Supplementary material for: Identification of MAPK12 as a Prognostic Biomarker for Esophageal Carcinoma Using Bioinformatics and Machine Learning
Source: Biomed Res Int. 2025 Dec 29;2025:2605071. doi: 10.1155/bmri/2605071 (PMC12746012; doi:10.1155/bmri/2605071)
Supplement: Supplementary file 1 — Supporting Information Additional supporting information can be found online in the Supporting Information section.. Supplementary Figure S1: Validation of the prognostic signature model based on the GEO database. Supplementary Figure S2: The linkage between the GO results and each gene and the linkage between the GO results. Supplementary Figure S3: Bar chart (a) and heat map (b) displaying the difference in pathway activities enriched by GSVA between the high‐risk and low‐risk groups. Supplementary Figure S4: The estimate score between the high‐risk and low‐risk groups. Supplementary Figure S5: Full uncropped Gels and Blots images of MAPK12. Supplementary Figure S6: Full uncropped Gels and Blots images of E‐cadrenin. Supplementary Figure S7: Full uncropped Gels and Blots images of N‐cadrenin. Supplementary Table S1: Clinical characteristics of patients with ESCA in the training datasets (TCGA) and the validation datasets (GEO). Supplementary Table S2: List of Tolement‐related genes included in the present study. Supplementary Table S3: List of differentially expressed genes (DEGs) between ESCA and normal lung tissues based on the TCGA database. Supplementary Table S4: List of 265 Tolement‐related DEGs by taking the intersection of DEGs of ESCA and Tolement‐related genes. Supplementary Table S5: 33 candidate genes with prognostic values were screened out by the Kaplan–Meier survival analysis. Supplementary Appendix 1. The detailed protocols for cell culture, transfection, and functional assays. Supplementary Appendix 2: The detailed statistical methods. [file BMRI-2025-2605071-s001.zip › Supplementary Appendix 2.docx]

1. Statistical Software and Significance Level

All statistical analyses were performed using R software 4.1.3.

A two-sided p-value < 0.05 was considered statistically significant for all tests unless otherwise specified in the figure legends or main text.

2. Overview of Statistical Tests Applied

The following table summarizes the specific statistical tests used for each type of data analysis in this study.

Table: Summary of Statistical Analyses

| Figure / Tables | Data Type and Purpose | Statistical Test(s) Applied | Post-hoc Test (if applicable) | Assumptions / Notes |
| --- | --- | --- | --- | --- |
| Supplementary Table 3 | RNA-seq TPM data; identifying genes differentially expressed between Tumor and Normal | DESeq2 (based on a negative binomial generalized linear model with Wald test for hypothesis testing) | Not Applicable | The model accounts for over-dispersed count data and library size differences. P-values were adjusted for multiple testing using the Benjamini-Hochberg (FDR) procedure. Genes with an FDR < 0.05 and \|log2FoldChange\| > 1.5 were considered significant |
| Supplementary Table 5 | Time-to-event (survival) data; assessing the association between the expression level of genes and overall survival (OS) | Univariate Cox proportional hazards regression model | Not Applicable | The results are presented as Hazard Ratios (HR) with 95% Confidence Intervals (CI). The proportional hazards assumption was verified using Schoenfeld residuals |
| Figure 3B, C | Time-to-event (survival) data with high-dimensional gene expression predictors; to identify the most prognostic genes and avoid overfitting | Least Absolute Shrinkage and Selection Operator (LASSO) Cox regression with 10-fold cross-validation | Not Applicable | The optimal penalty parameter (lambda) was selected based on the **minimum criterion (lambda.min)**or the**one-standard-error rule (lambda.1se)** from the cross-validation, which shrinks the coefficients of non-informative genes to zero |
| Figure 3D | Time-to-event (survival) data; to identify genes from the LASSO analysis that serve as independent prognostic factors when adjusted for each other | Multivariate Cox proportional hazards regression model | Not Applicable | The genes selected by the LASSO regression were included as covariates in the model. The results are presented as Hazard Ratios (HR) with 95% Confidence Intervals (CI). The proportional hazards assumption was verified |
| Figure 3F, Supplementary Figure 1 | Time-to-event (survival) data; to evaluate the predictive accuracy of the prognostic signature for 1-, 3-, and 5-year overall survival (OS) | Time-dependent Receiver Operating Characteristic (ROC) curve analysis. The Area Under the Curve (AUC) was calculated to quantify the discrimination ability at each time point | Not Applicable | This method assesses how well the risk score separates patients who die before a specific time point from those who survive beyond it. An AUC > 0.7 is generally considered to indicate good predictive ability. The analysis was performed using the R package survivalROC or timeROC |
| Figure 3G, Supplementary Figure 1, Figure 4A | Time-to-event (survival) data; to compare the Overall Survival (OS) between two independent groups (Low-risk vs. High-risk) | Kaplan-Meier estimator for survival curve visualization, with the Log-rank (Mantel-Cox) test for hypothesis testing | Not Applicable | The Kaplan-Meier method is non-parametric. The Log-rank test is used to compare the entire survival experience between groups. A p-value < 0.05 indicates a statistically significant difference in survival distributions |
| Figure 3I  Figure 8A-C  Figure 10A–F | Comparison of a continuous variable between two independent groups | Unpaired two-tailed Student's t-test (for data meeting normality and homogeneity of variance) | Not Applicable | Data are presented as mean ± SD. Normality was assessed using the Shapiro-Wilk test; if violated, the non-parametric Mann-Whitney U test was used instead |
| Figure 4B,  Figure 9A-C | Comparison of a continuous variable across multiple independent groups | One-way ANOVA | Tukey's HSD test | Used to determine which specific stage pairs differed significantly after a significant overall test (p < 0.05). The p-values for significant pairwise comparisons are reported in the results (p < 0.05) |
| Figure 12  Figure 13 | Comparison of a continuous but not-normally distributed or ordinal variable | Mann-Whitney U test (also known as Wilcoxon rank-sum test) | Not Applicable | This non-parametric test is used when the data do not meet the normality assumption of the t-test. Data are typically presented as **median with interquartile range (IQR)** |
