# Supplementary material for: Identification of MAPK12 as a Prognostic Biomarker for Esophageal Carcinoma Using Bioinformatics and Machine Learning
Source: Biomed Res Int. 2025 Dec 29;2025:2605071. doi: 10.1155/bmri/2605071 (PMC12746012; doi:10.1155/bmri/2605071)
Supplement: Supplementary file 1 — Supporting Information Additional supporting information can be found online in the Supporting Information section.. Supplementary Figure S1: Validation of the prognostic signature model based on the GEO database. Supplementary Figure S2: The linkage between the GO results and each gene and the linkage between the GO results. Supplementary Figure S3: Bar chart (a) and heat map (b) displaying the difference in pathway activities enriched by GSVA between the high‐risk and low‐risk groups. Supplementary Figure S4: The estimate score between the high‐risk and low‐risk groups. Supplementary Figure S5: Full uncropped Gels and Blots images of MAPK12. Supplementary Figure S6: Full uncropped Gels and Blots images of E‐cadrenin. Supplementary Figure S7: Full uncropped Gels and Blots images of N‐cadrenin. Supplementary Table S1: Clinical characteristics of patients with ESCA in the training datasets (TCGA) and the validation datasets (GEO). Supplementary Table S2: List of Tolement‐related genes included in the present study. Supplementary Table S3: List of differentially expressed genes (DEGs) between ESCA and normal lung tissues based on the TCGA database. Supplementary Table S4: List of 265 Tolement‐related DEGs by taking the intersection of DEGs of ESCA and Tolement‐related genes. Supplementary Table S5: 33 candidate genes with prognostic values were screened out by the Kaplan–Meier survival analysis. Supplementary Appendix 1. The detailed protocols for cell culture, transfection, and functional assays. Supplementary Appendix 2: The detailed statistical methods. [file BMRI-2025-2605071-s001.zip › Supplementary Table 1.docx]

**Supplementary Table1** Clinical characteristics of lung adenocarcinoma patients in the training datasets (TCGA) and the validation datasets (GEO)

| **Characteristics** | **TCGA**  **(*N* = 166)** | **GSE53625**  **(*N* = 179)** |
| --- | --- | --- |
| **Age** |  |  |
| <65 | 94 | 52 |
| >=65 | 72 | 127 |
| **Gender** |  |  |
| Male | 136 | 146 |
| Female | 30 | 33 |
| **Stage** |  |  |
| I | 24 | 10 |
| II | 75 | 77 |
| III | 59 | 92 |
| IV | 8 | / |
| **Status** |  |  |
| Alive | 64 | 102 |
| Dead | 102 | 77 |
