# Supplementary material for: Identification of MAPK12 as a Prognostic Biomarker for Esophageal Carcinoma Using Bioinformatics and Machine Learning
Source: Biomed Res Int. 2025 Dec 29;2025:2605071. doi: 10.1155/bmri/2605071 (PMC12746012; doi:10.1155/bmri/2605071)
Supplement: Supplementary file 1 — Supporting Information Additional supporting information can be found online in the Supporting Information section.. Supplementary Figure S1: Validation of the prognostic signature model based on the GEO database. Supplementary Figure S2: The linkage between the GO results and each gene and the linkage between the GO results. Supplementary Figure S3: Bar chart (a) and heat map (b) displaying the difference in pathway activities enriched by GSVA between the high‐risk and low‐risk groups. Supplementary Figure S4: The estimate score between the high‐risk and low‐risk groups. Supplementary Figure S5: Full uncropped Gels and Blots images of MAPK12. Supplementary Figure S6: Full uncropped Gels and Blots images of E‐cadrenin. Supplementary Figure S7: Full uncropped Gels and Blots images of N‐cadrenin. Supplementary Table S1: Clinical characteristics of patients with ESCA in the training datasets (TCGA) and the validation datasets (GEO). Supplementary Table S2: List of Tolement‐related genes included in the present study. Supplementary Table S3: List of differentially expressed genes (DEGs) between ESCA and normal lung tissues based on the TCGA database. Supplementary Table S4: List of 265 Tolement‐related DEGs by taking the intersection of DEGs of ESCA and Tolement‐related genes. Supplementary Table S5: 33 candidate genes with prognostic values were screened out by the Kaplan–Meier survival analysis. Supplementary Appendix 1. The detailed protocols for cell culture, transfection, and functional assays. Supplementary Appendix 2: The detailed statistical methods. [file BMRI-2025-2605071-s001.zip › Supplymentary Table 5.pdf]

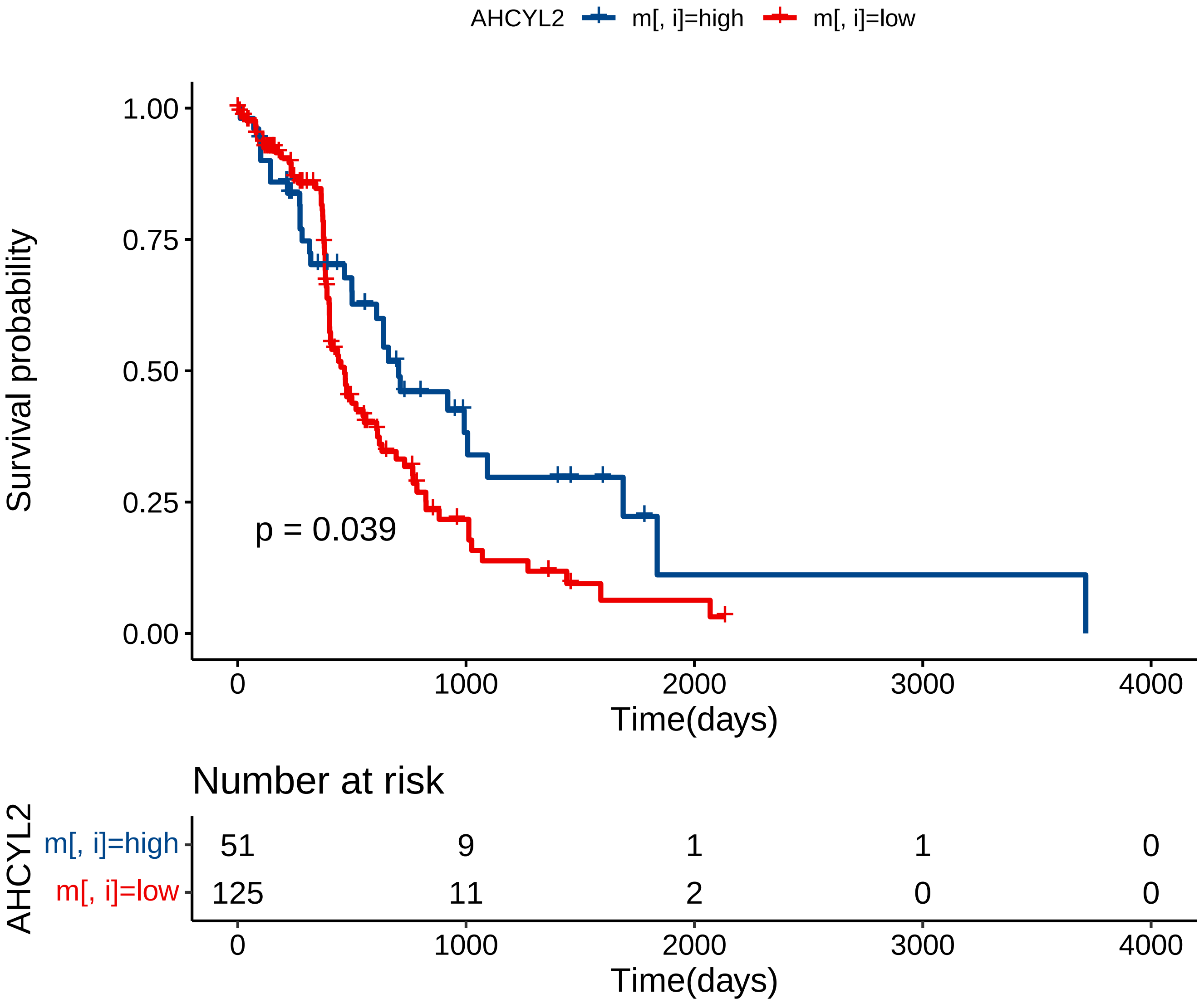

AHCYL2
m[, i]=high
m[, i]=low
Survival probability
1.00
0.75
0.50
0.25
0.00
p = 0.039
0
1000
2000
3000
4000
Time(days)
Number at risk
AHCYL2
m[, i]=high
m[, i]=low
51 9 1 1 0
125 11 2 0 0
0
1000
2000
3000
4000
Time(days)

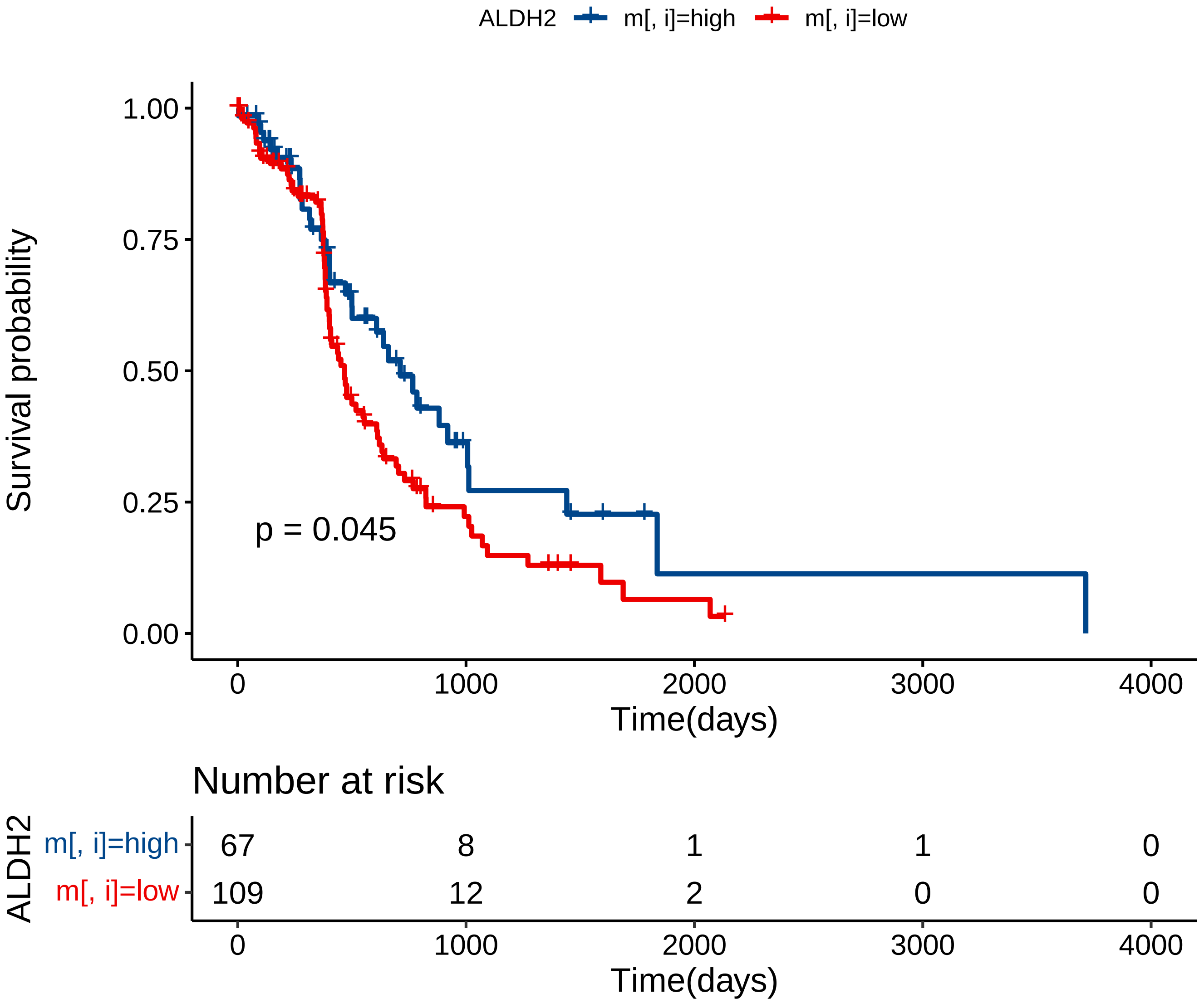

ALDH2
m[, i]=high
m[, i]=low
Survival probability
1.00
0.75
0.50
0.25
0.00
p = 0.045
0
1000
2000
3000
4000
Time(days)
Number at risk
ALDH2
m[, i]=high
m[, i]=low
67
8
1
1
0
109
12
2
0
0
0
1000
2000
3000
4000
Time(days)

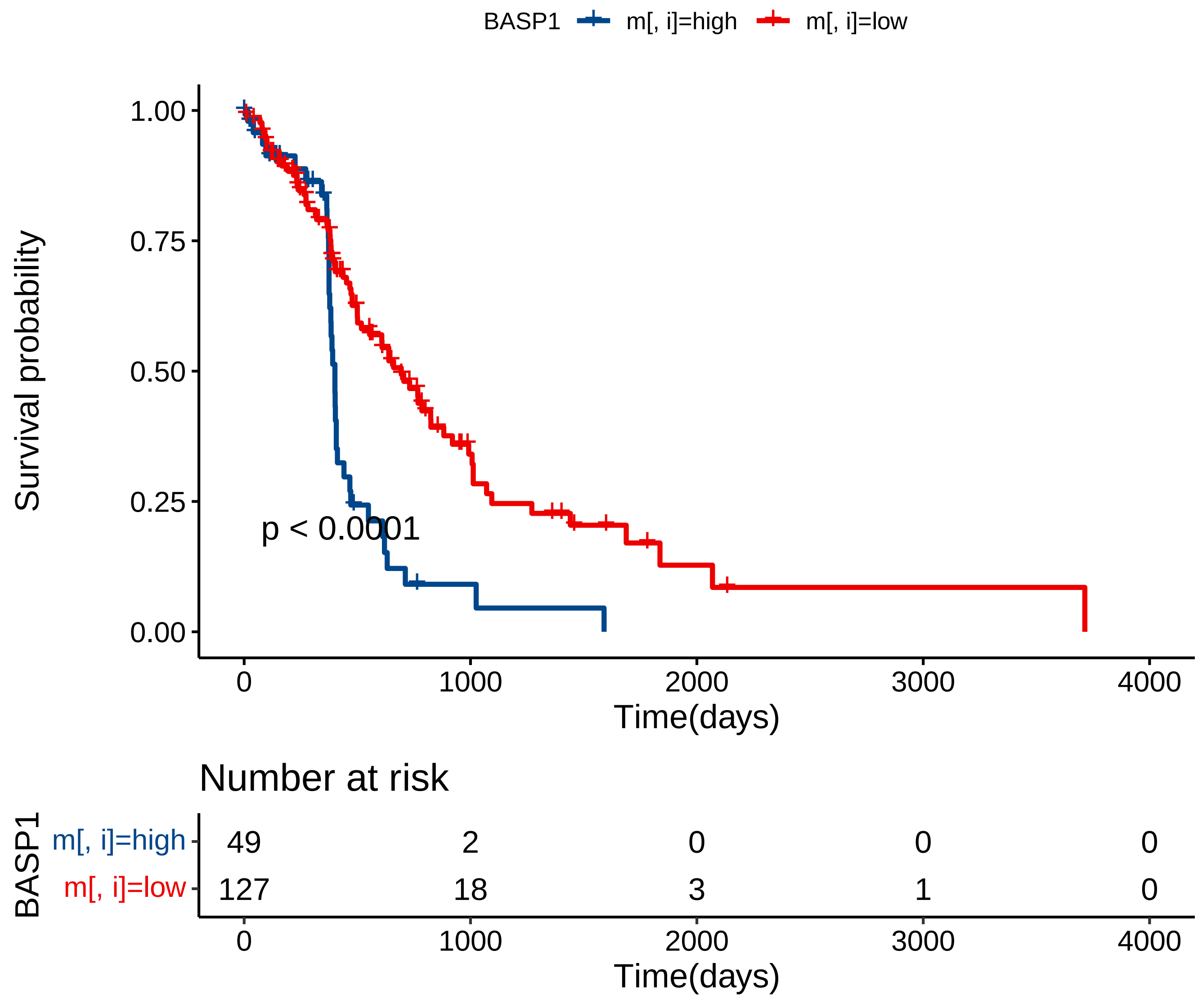
BASP1
m[, i]=high
m[, i]=low
Survival probability
1.00
0.75
0.50
0.25
0.00
p < 0.0001
0
1000
2000
3000
4000
Time(days)
Number at risk
BASP1
m[, i]=high 49 2 0 0 0
m[, i]=low 127 18 3 1 0
0
1000
2000
3000
4000
Time(days)

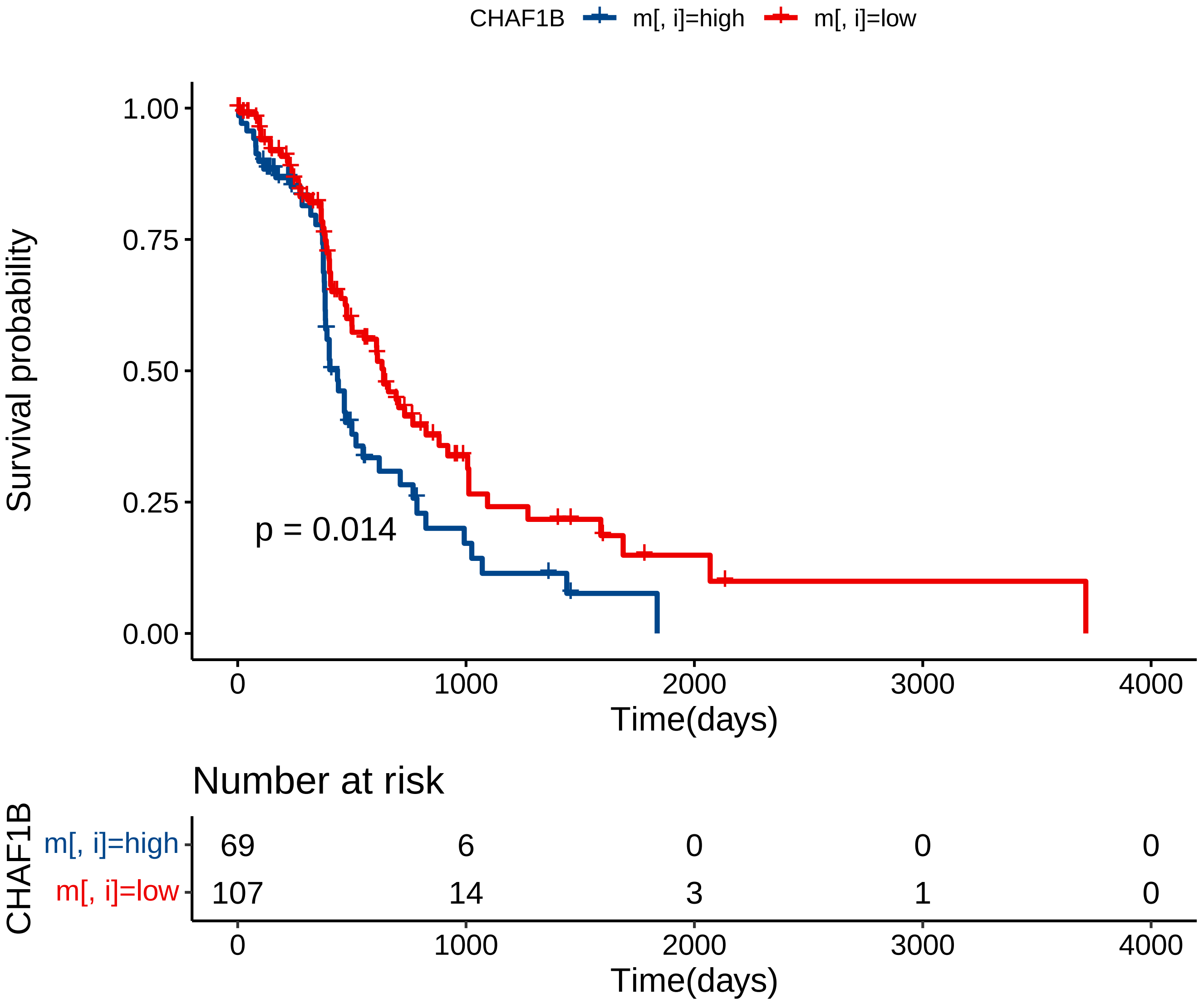
CHAF1B
m[, i]=high
m[, i]=low
Survival probability
1.00
0.75
0.50
0.25
0.00
p = 0.014
0
1000
2000
3000
4000
Time(days)
Number at risk
CHAF1B
m[, i]=high
m[, i]=low
69
6
0
0
0
107
14
3
1
0
0
1000
2000
3000
4000
Time(days)

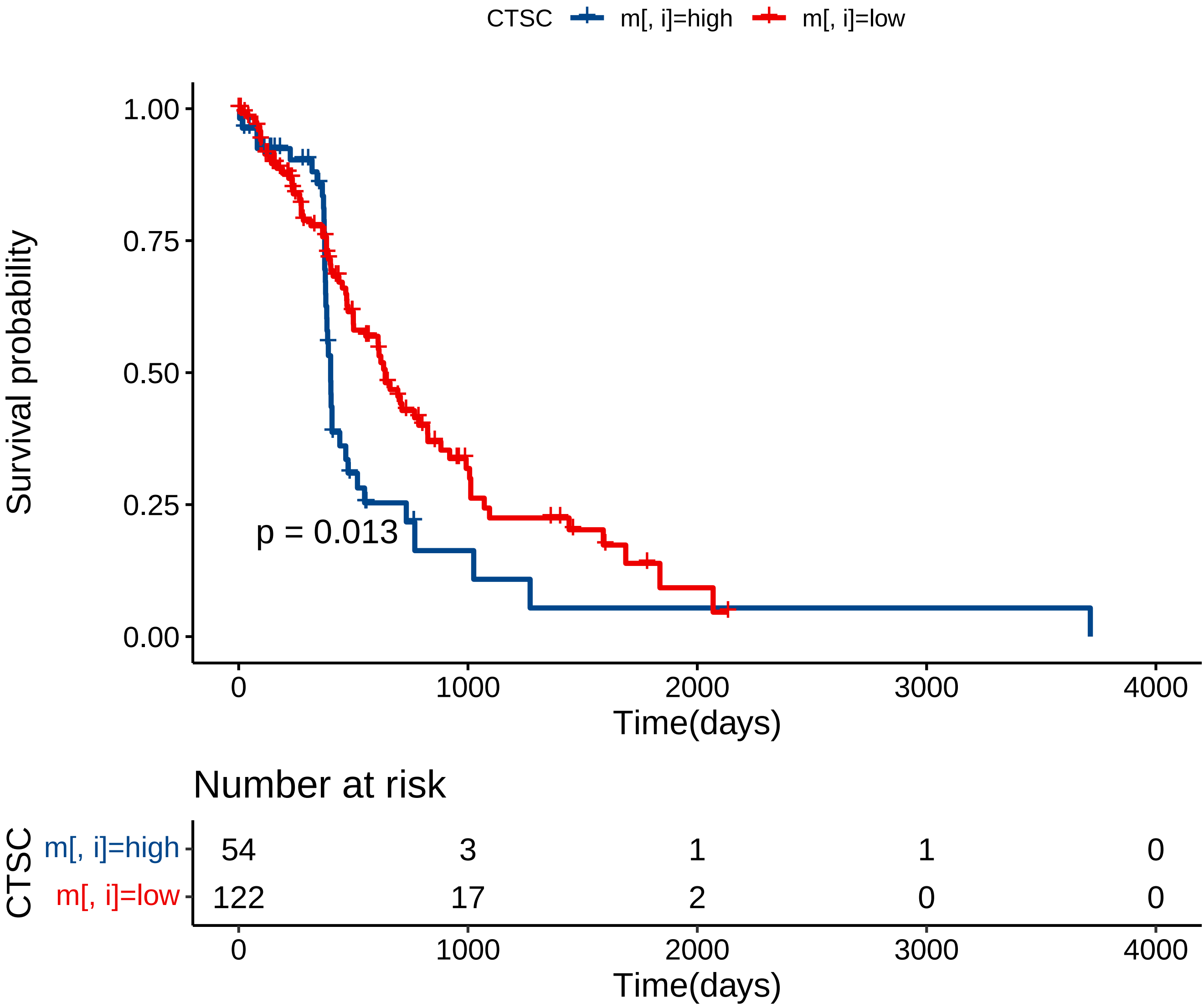
CTSC
m[, i]=high
m[, i]=low
Survival probability
1.00
0.75
0.50
0.25
0.00
p = 0.013
0
1000
2000
3000
4000
Time(days)
Number at risk
CTSC
m[, i]=high
m[, i]=low
54
3
1
1
0
122
17
2
0
0
0
1000
2000
3000
4000
Time(days)

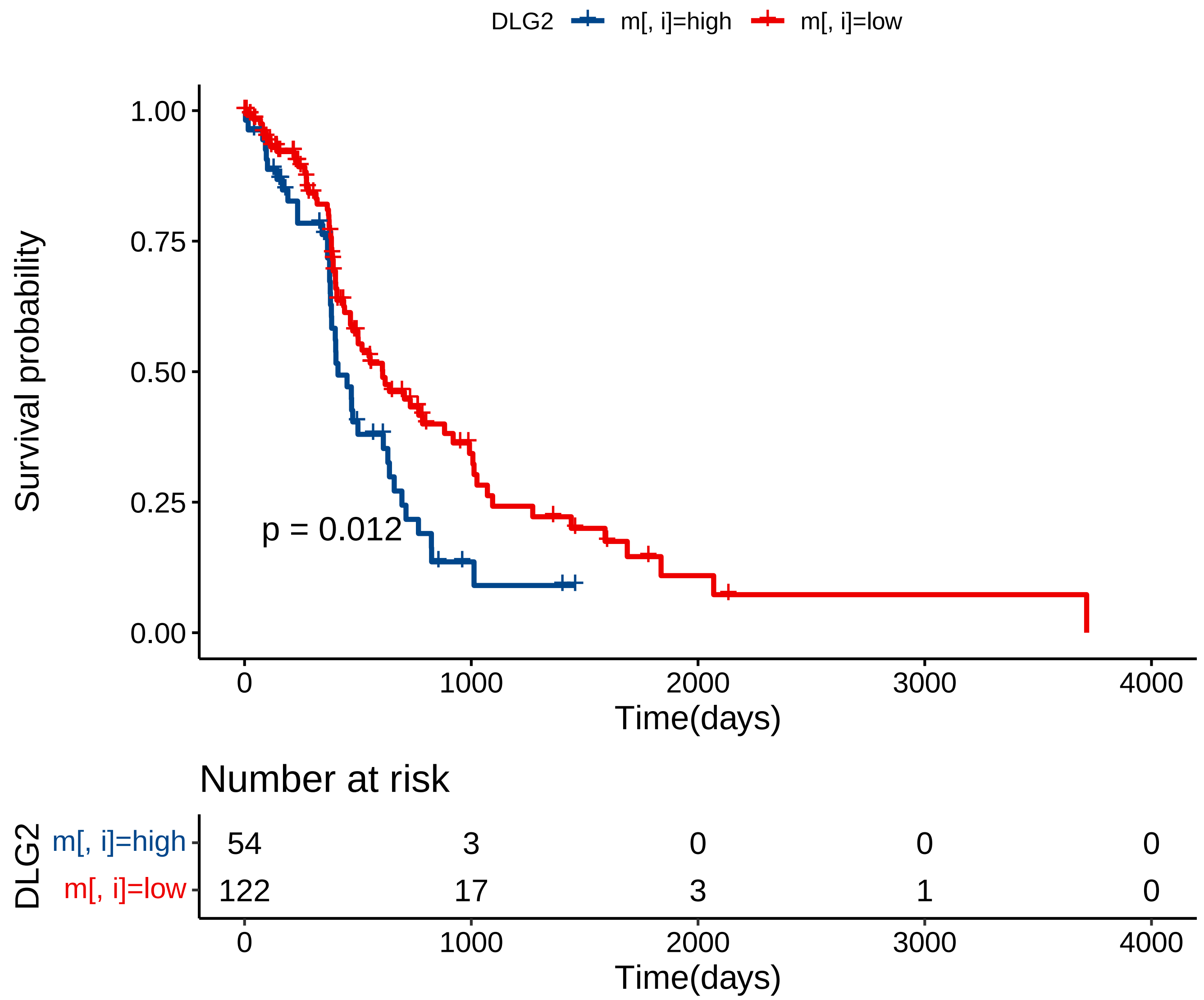
DLG2
m[, i]=high
m[, i]=low
Survival probability
1.00
0.75
0.50
0.25
0.00
p = 0.012
0
1000
2000
3000
4000
Time(days)
Number at risk
DLG2
m[, i]=high
m[, i]=low
54
3
0
0
0
122
17
3
1
0
0
1000
2000
3000
4000
Time(days)

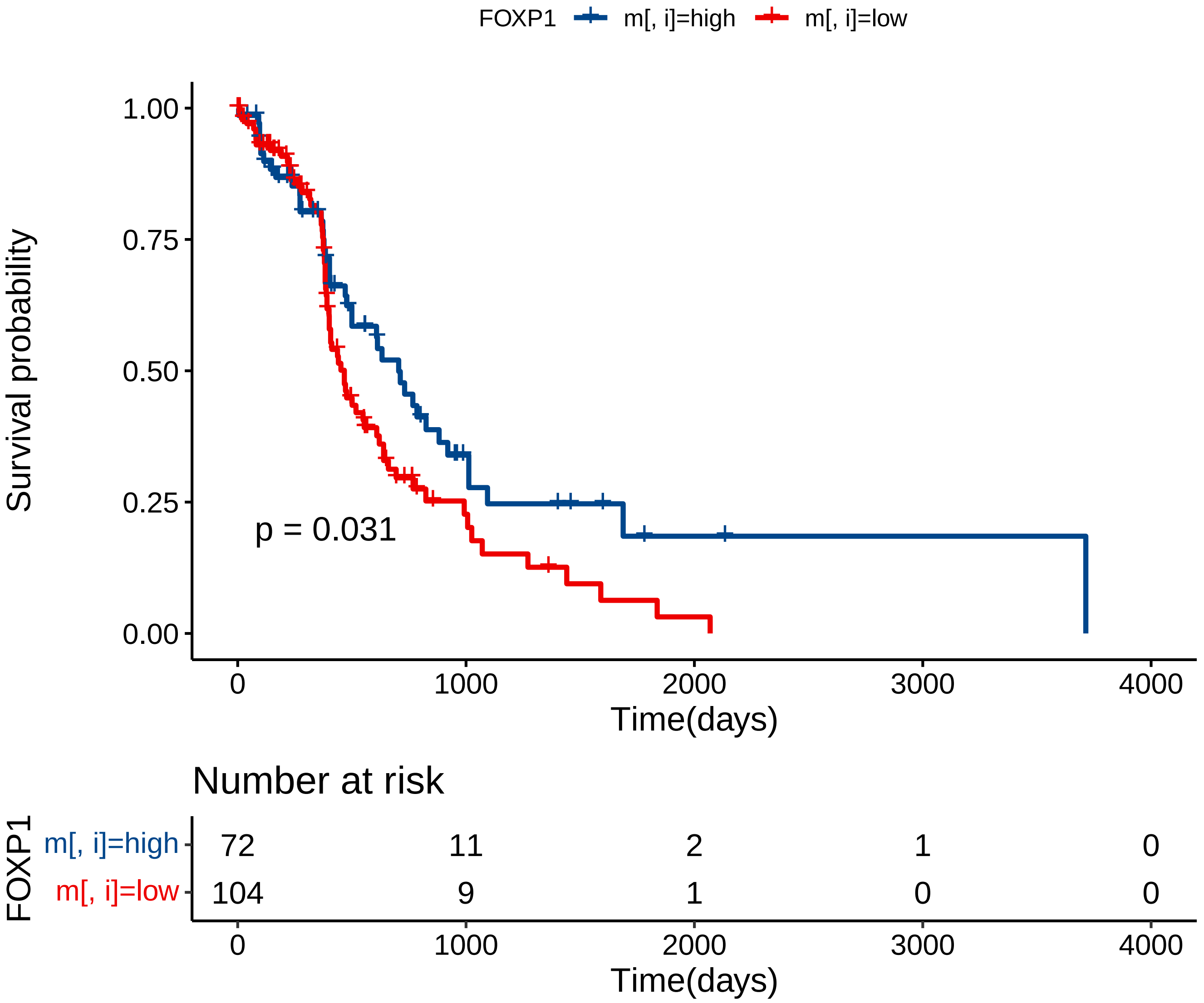

FOXP1
m[, i]=high
m[, i]=low
Survival probability
1.00
0.75
0.50
0.25
0.00
p = 0.031
0
1000
2000
3000
4000
Time(days)
Number at risk
FOXP1
m[, i]=high 72 11 2 1 0
m[, i]=low 104 9 1 0 0
0
1000
2000
3000
4000
Time(days)

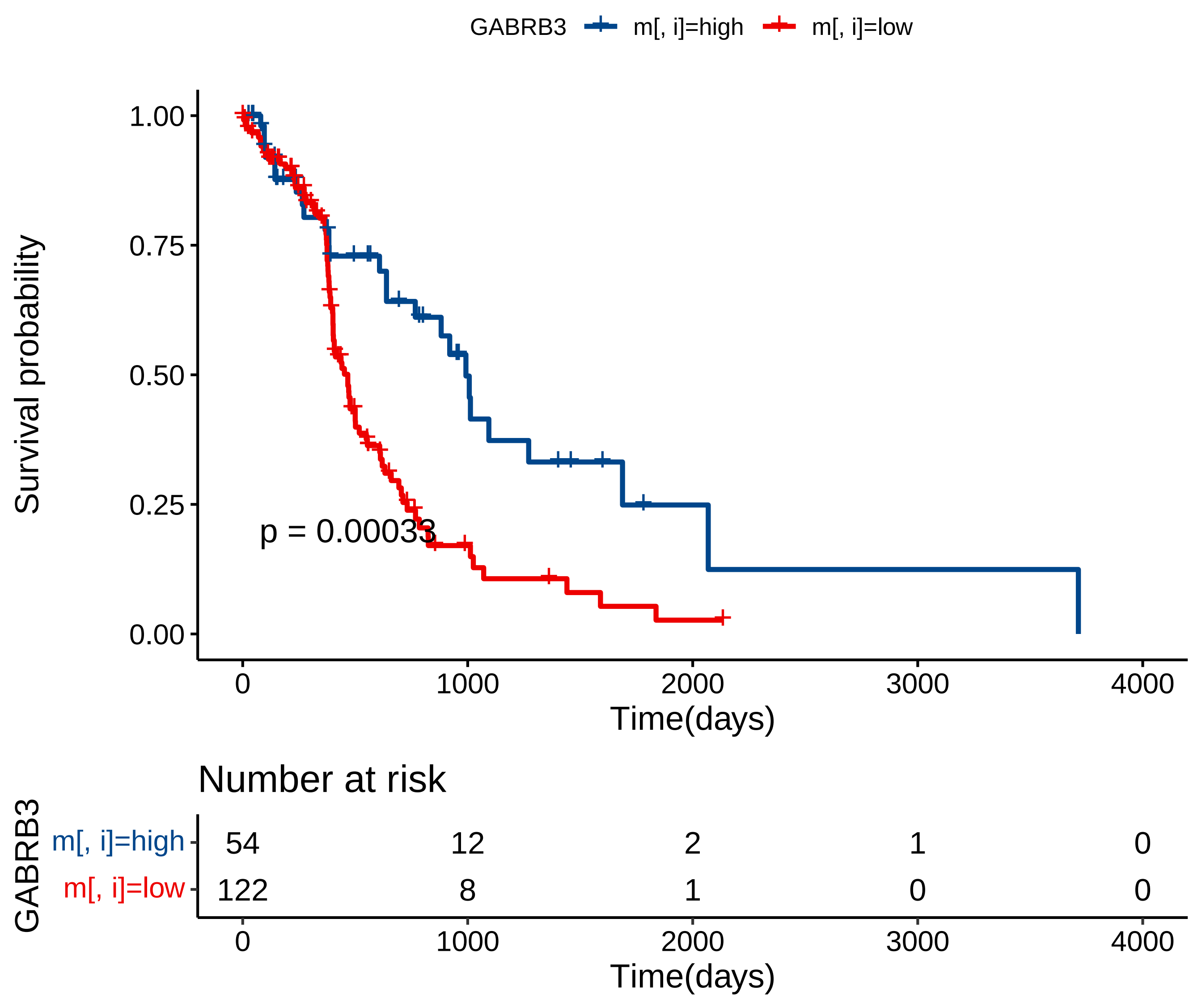

GABRB3
m[, i]=high
m[, i]=low
Survival probability
1.00
0.75
0.50
0.25
0.00
p = 0.00033
0
1000
2000
3000
4000
Time(days)
Number at risk
GABRB3
m[, i]=high 54 12 2 1 0
m[, i]=low 122 8 1 0 0
0
1000
2000
3000
4000
Time(days)

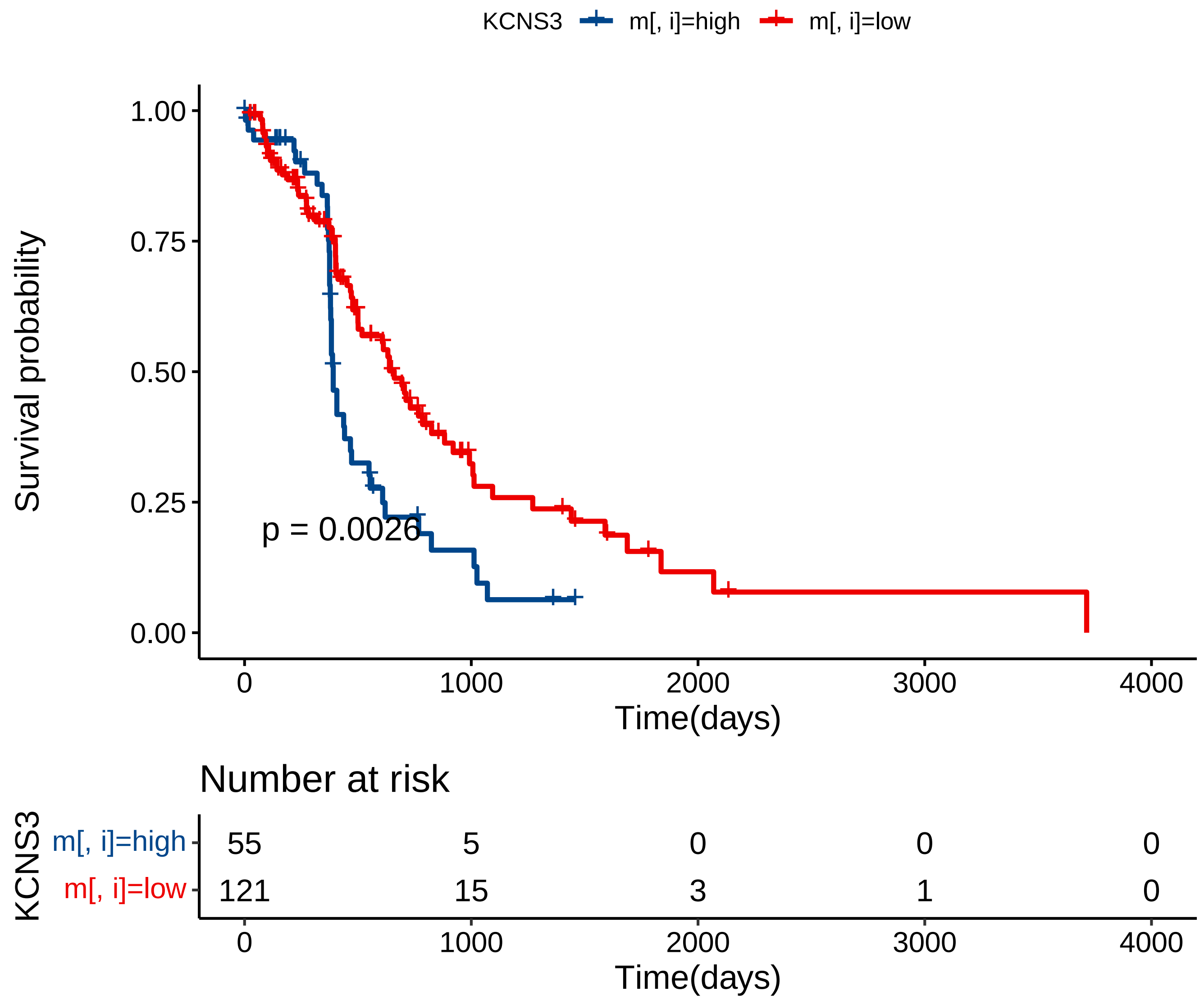
KCNS3
m[, i]=high
m[, i]=low
1.00
0.75
0.50
0.25
0.00
Survival probability
p = 0.0026
0
1000
2000
3000
4000
Time(days)
Number at risk
KCNS3
m[, i]=high
m[, i]=low
55
5
0
0
0
121
15
3
1
0
0
1000
2000
3000
4000
Time(days)

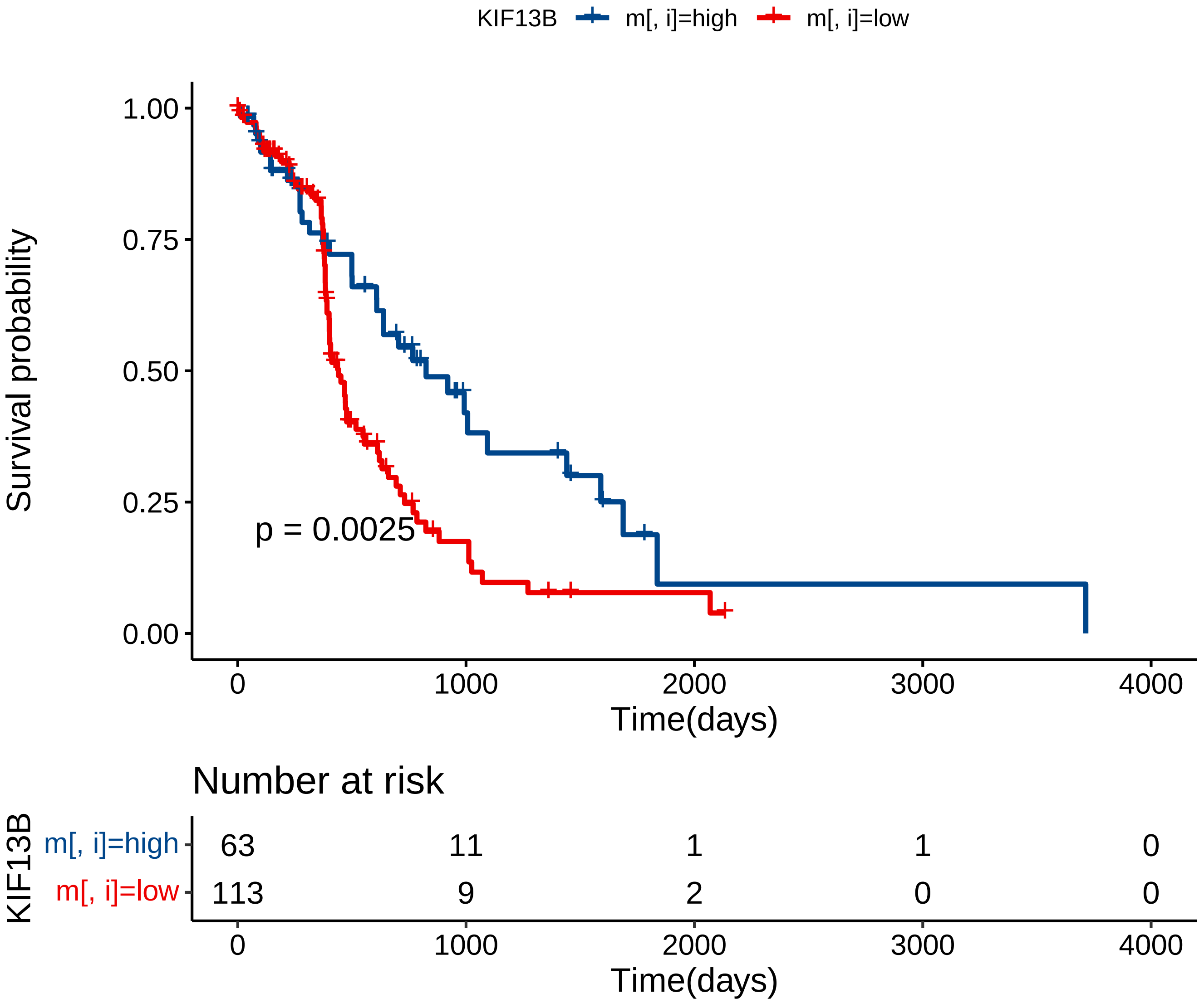

KIF13B
m[, i]=high
m[, i]=low
Survival probability
1.00
0.75
0.50
0.25
0.00
p = 0.0025
0
1000
2000
3000
4000
Time(days)
Number at risk
KIF13B
m[, i]=high
m[, i]=low
63
11
1
1
0
113
9
2
0
0
0
1000
2000
3000
4000
Time(days)

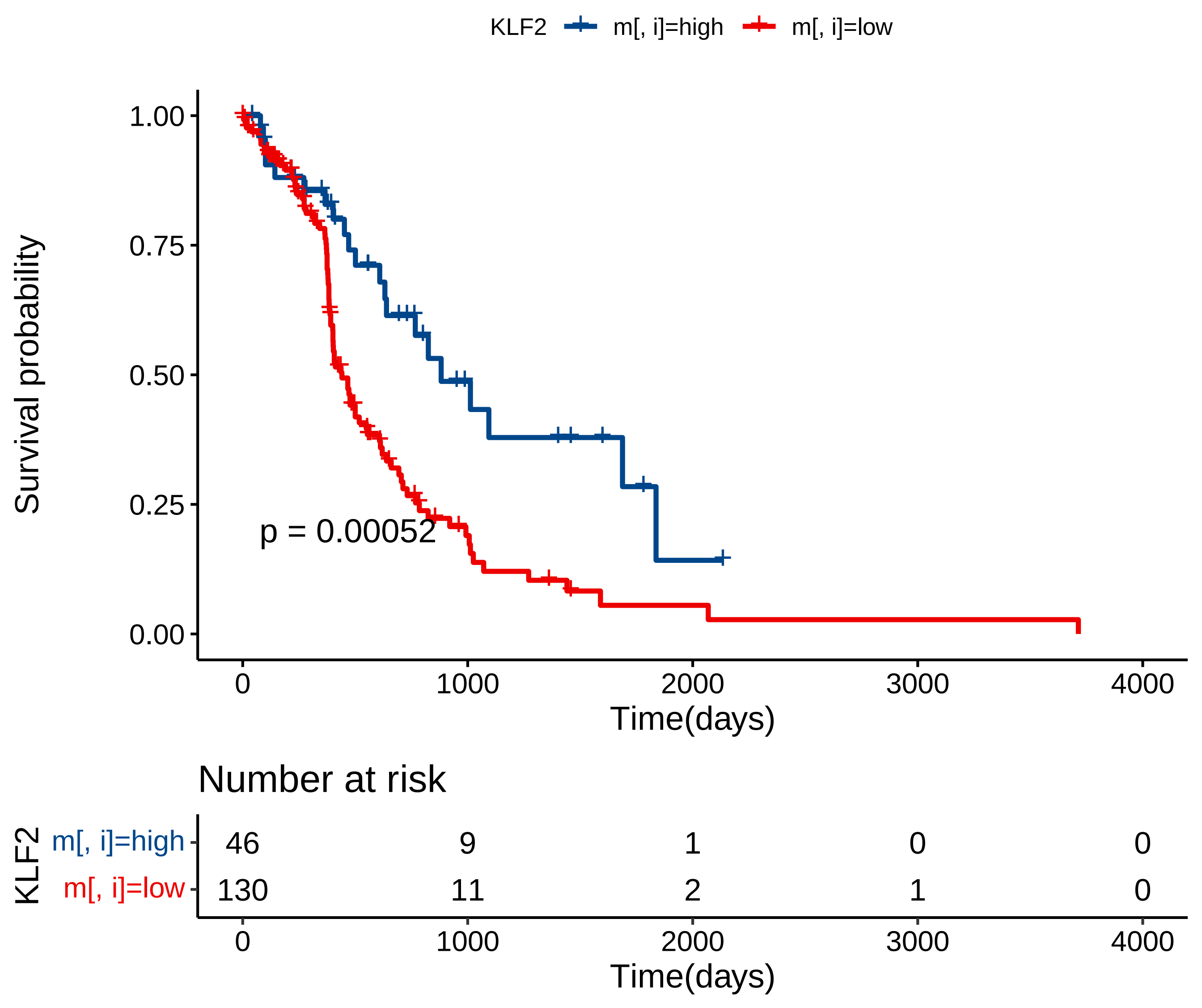
KLF2
m[, i]=high
m[, i]=low
Survival probability
1.00
0.75
0.50
0.25
0.00
p = 0.00052
0
1000
2000
3000
4000
Time(days)
Number at risk
KLF2
m[, i]=high
m[, i]=low
46
9
1
0
0
130
11
2
1
0
0
1000
2000
3000
4000
Time(days)

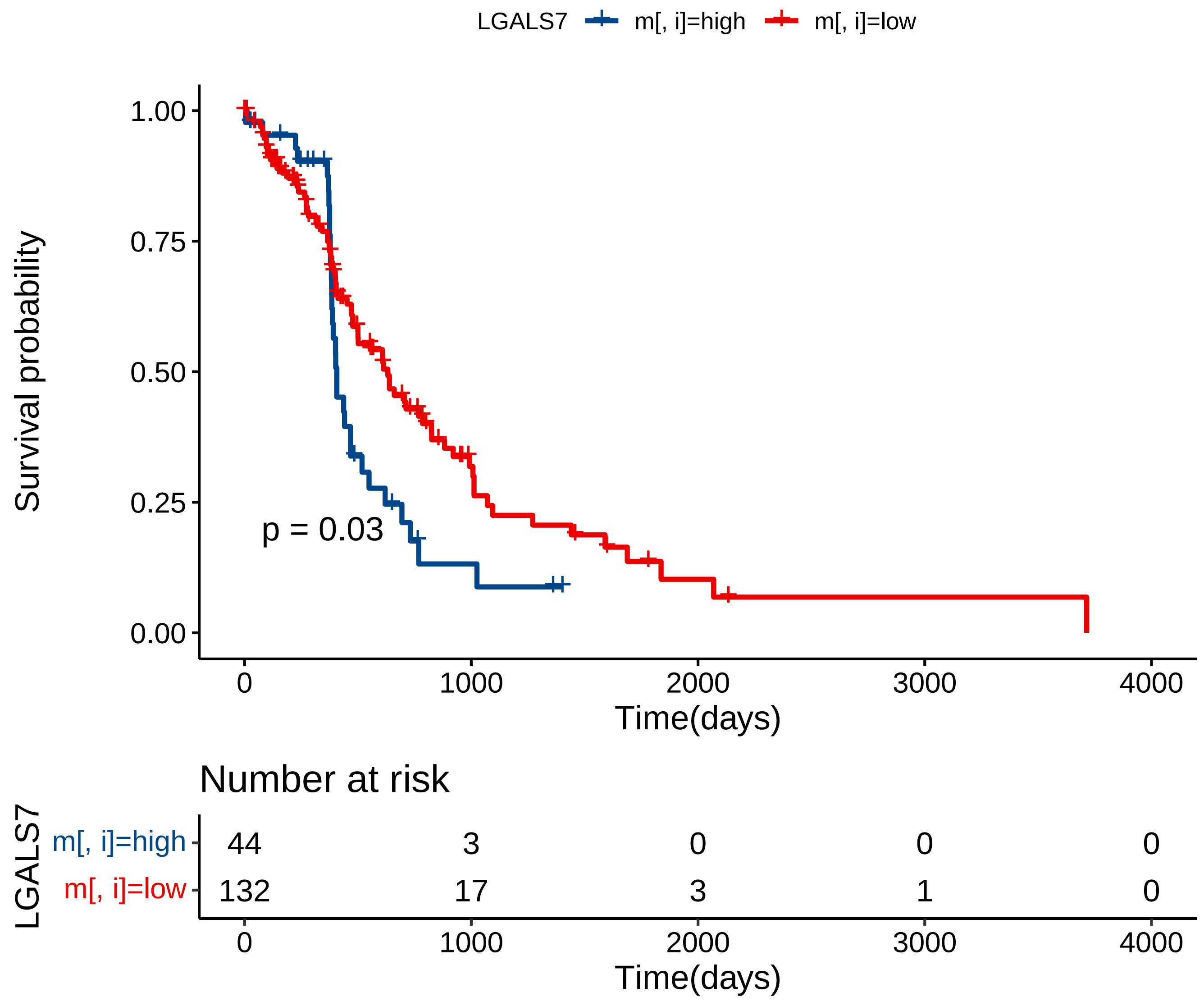

LGALS7
m[, i]=high
m[, i]=low
Survival probability
1.00
0.75
0.50
0.25
0.00
p = 0.03
0
1000
2000
3000
4000
Time(days)
Number at risk
LGALS7
m[, i]=high
m[, i]=low
44
3
0
0
0
132
17
3
1
0
0
1000
2000
3000
4000
Time(days)

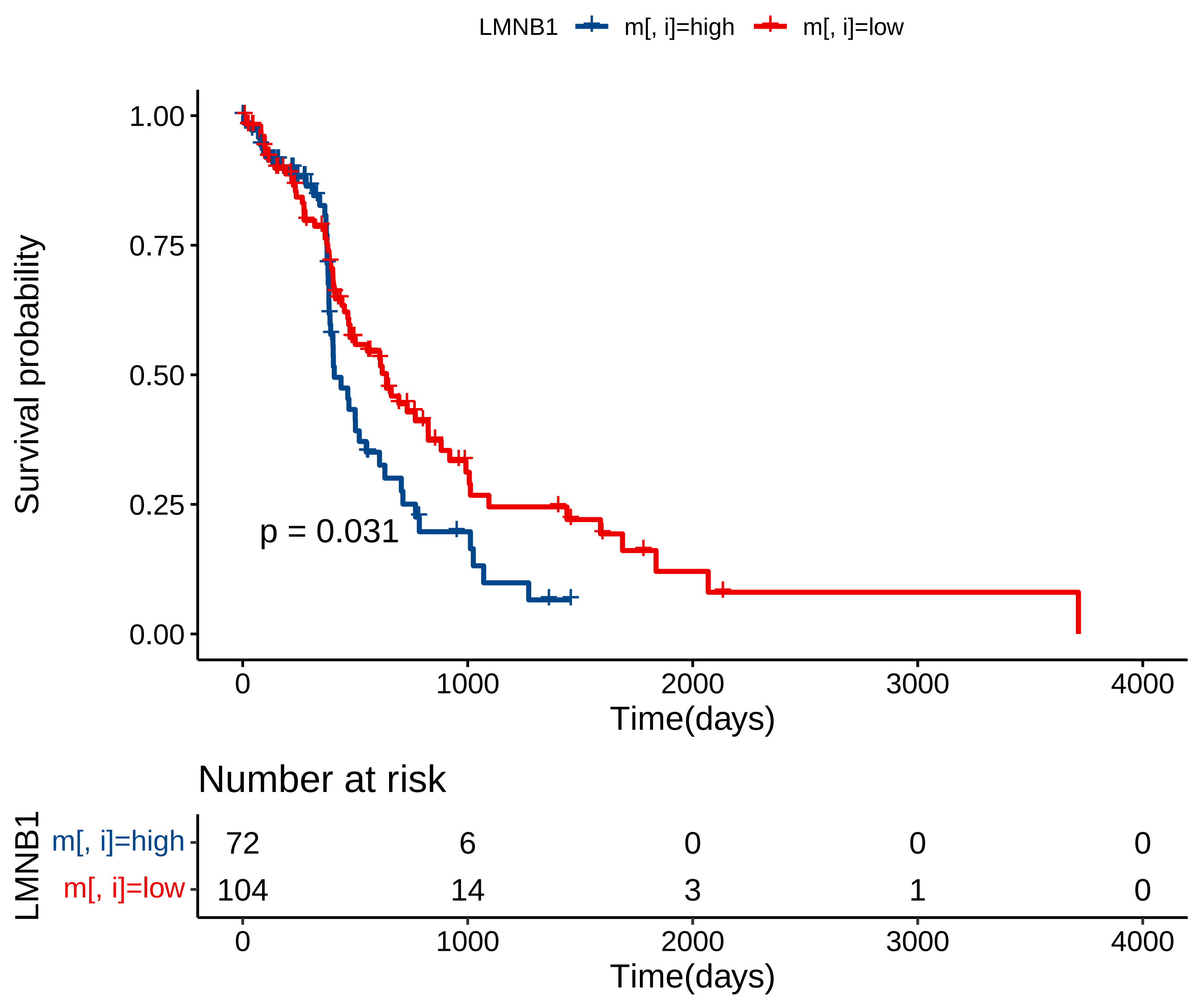
LMNB1
m[, i]=high
m[, i]=low
1.00
0.75
0.50
0.25
0.00
Survival probability
p = 0.031
0
1000
2000
3000
4000
Time(days)
Number at risk
LMNB1
m[, i]=high
m[, i]=low
72
6
0
0
0
104
14
3
1
0
0
1000
2000
3000
4000
Time(days)

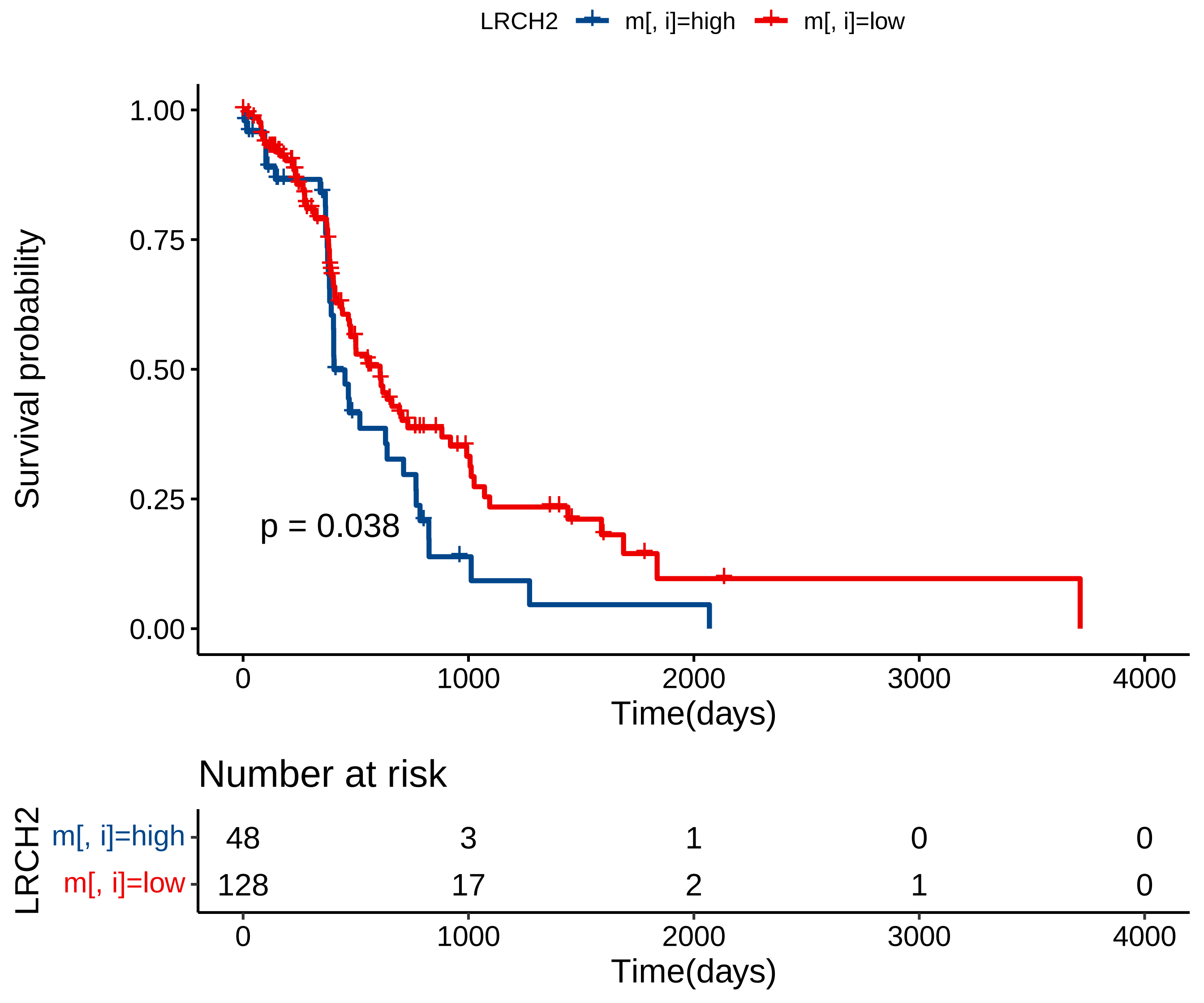

LRCH2
m[, i]=high
m[, i]=low
Survival probability
1.00
0.75
0.50
0.25
0.00
p = 0.038
0
1000
2000
3000
4000
Time(days)
Number at risk
LRCH2
m[, i]=high 48 3 1 0 0
m[, i]=low 128 17 2 1 0
0
1000
2000
3000
4000
Time(days)

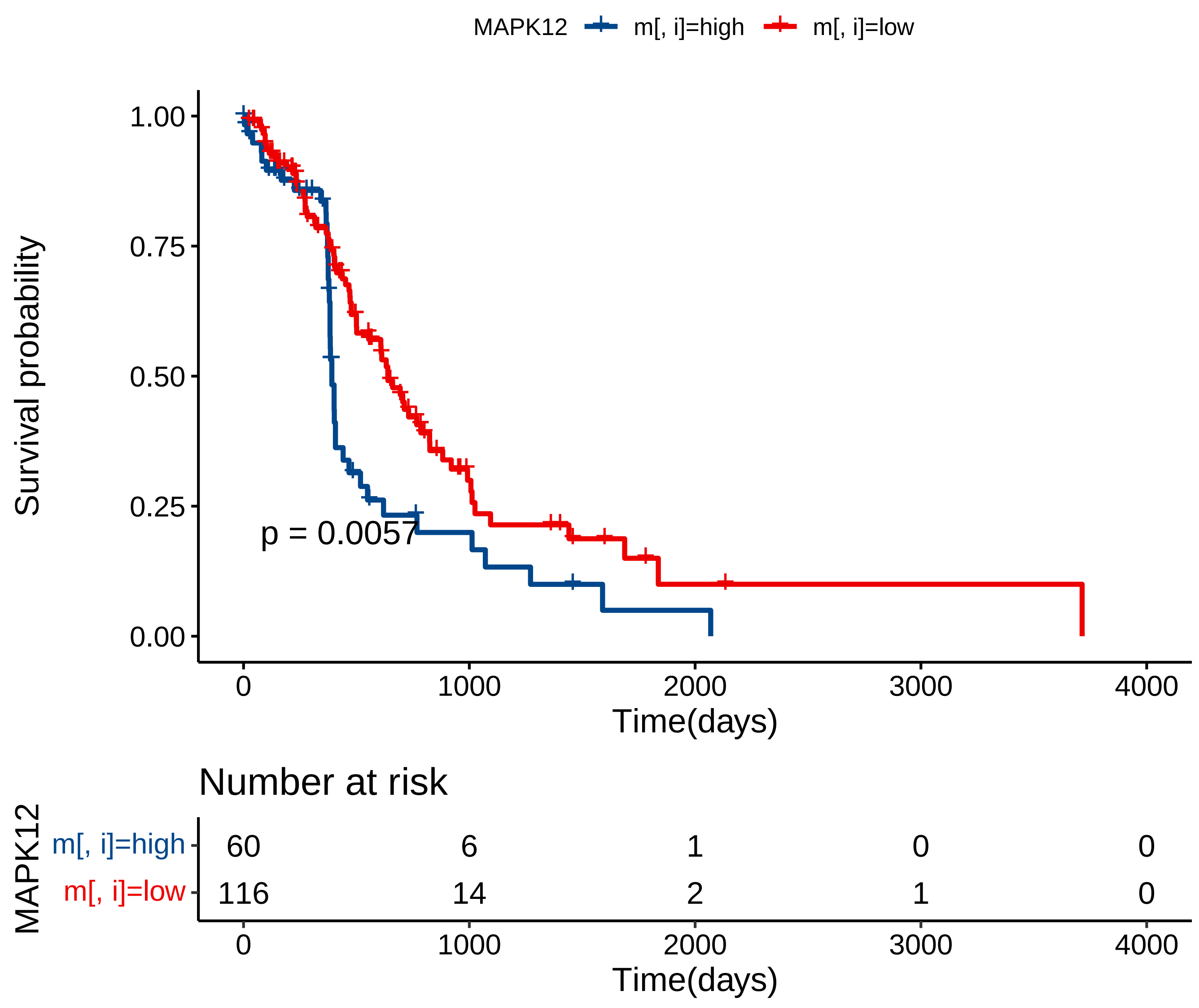
MAPK12
m[, i]=high
m[, i]=low
Survival probability
1.00
0.75
0.50
0.25
0.00
p = 0.0057
0
1000
2000
3000
4000
Time(days)
Number at risk
MAPK12
m[, i]=high 60 6 1 0 0
m[, i]=low 116 14 2 1 0
Time(days)

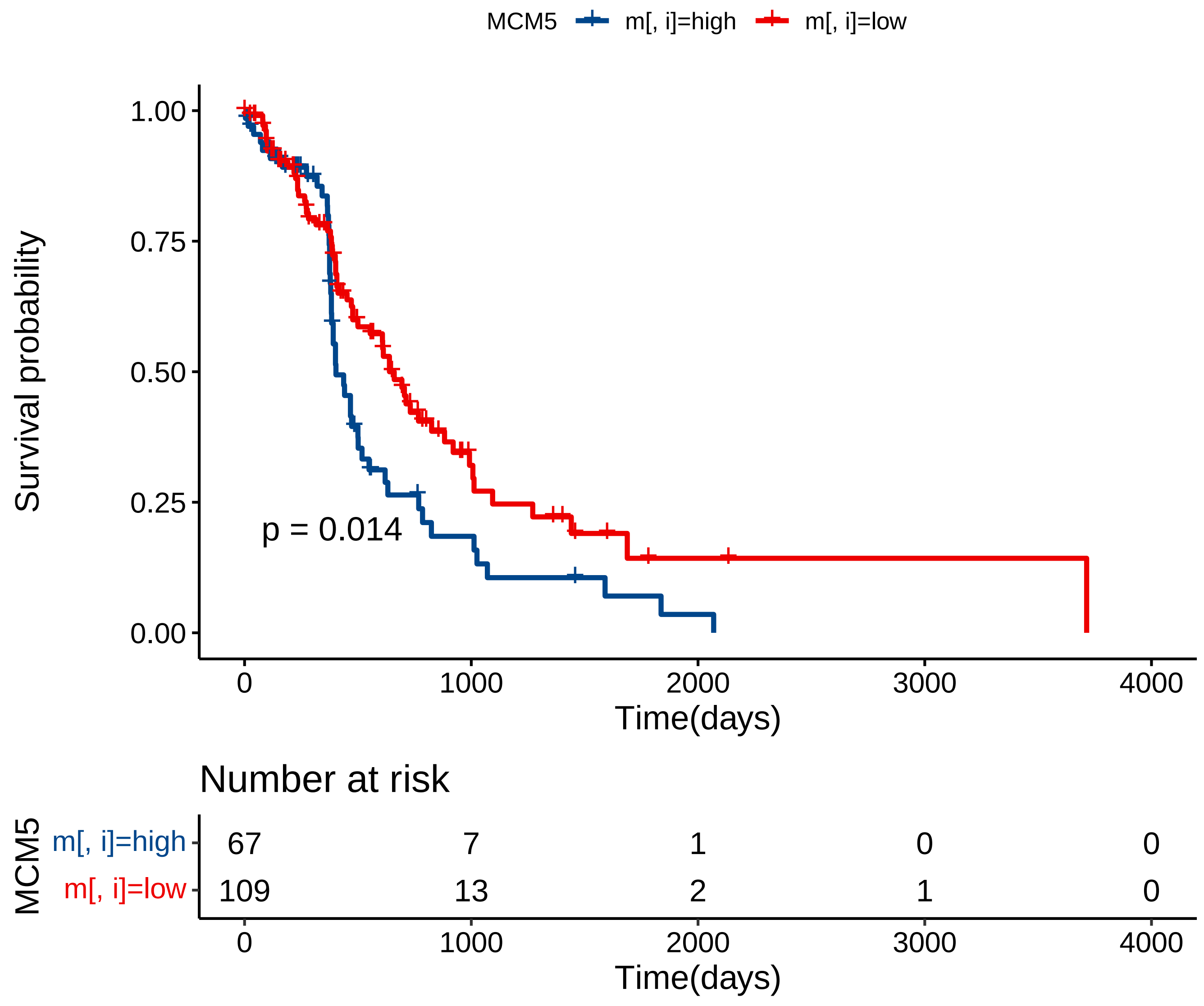

MCM5
m[, i]=high
m[, i]=low
Survival probability
1.00
0.75
0.50
0.25
0.00
p = 0.014
0
1000
2000
3000
4000
Time(days)
Number at risk
MCM5
m[, i]=high 67 7 1 0 0
m[, i]=low 109 13 2 1 0
0
1000
2000
3000
4000
Time(days)

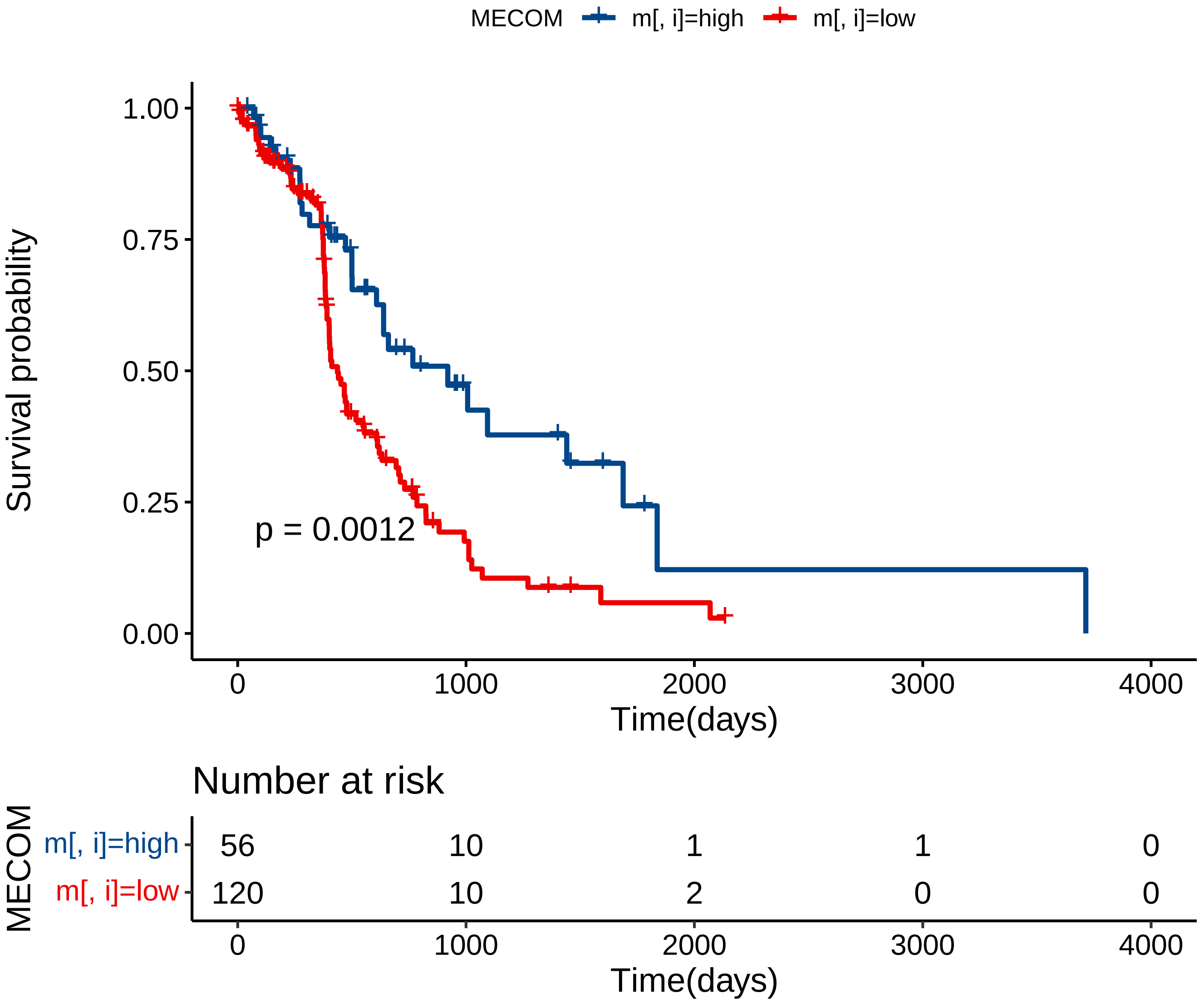

MECOM
m[, i]=high
m[, i]=low
Survival probability
1.00
0.75
0.50
0.25
0.00
p = 0.0012
0
1000
2000
3000
4000
Time(days)
Number at risk
MECOM
m[, i]=high
m[, i]=low
56
10
1
1
0
120
10
2
0
0
0
1000
2000
3000
4000
Time(days)

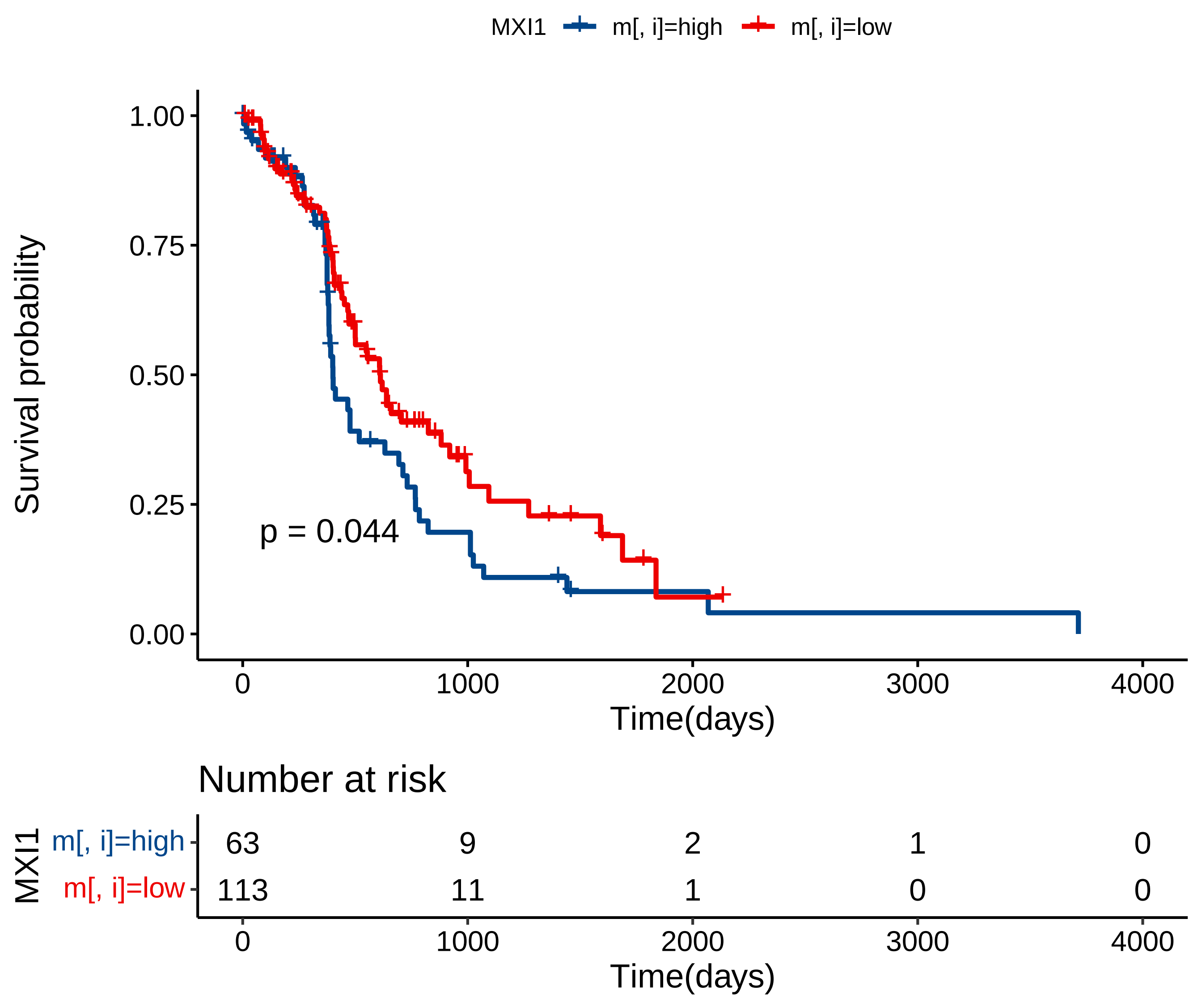
MXI1
m[, i]=high
m[, i]=low
Survival probability
1.00
0.75
0.50
0.25
0.00
p = 0.044
0
1000
2000
3000
4000
Time(days)
Number at risk
MXI1
m[, i]=high
63
9
2
1
0
m[, i]=low
113
11
1
0
0
0
1000
2000
3000
4000
Time(days)

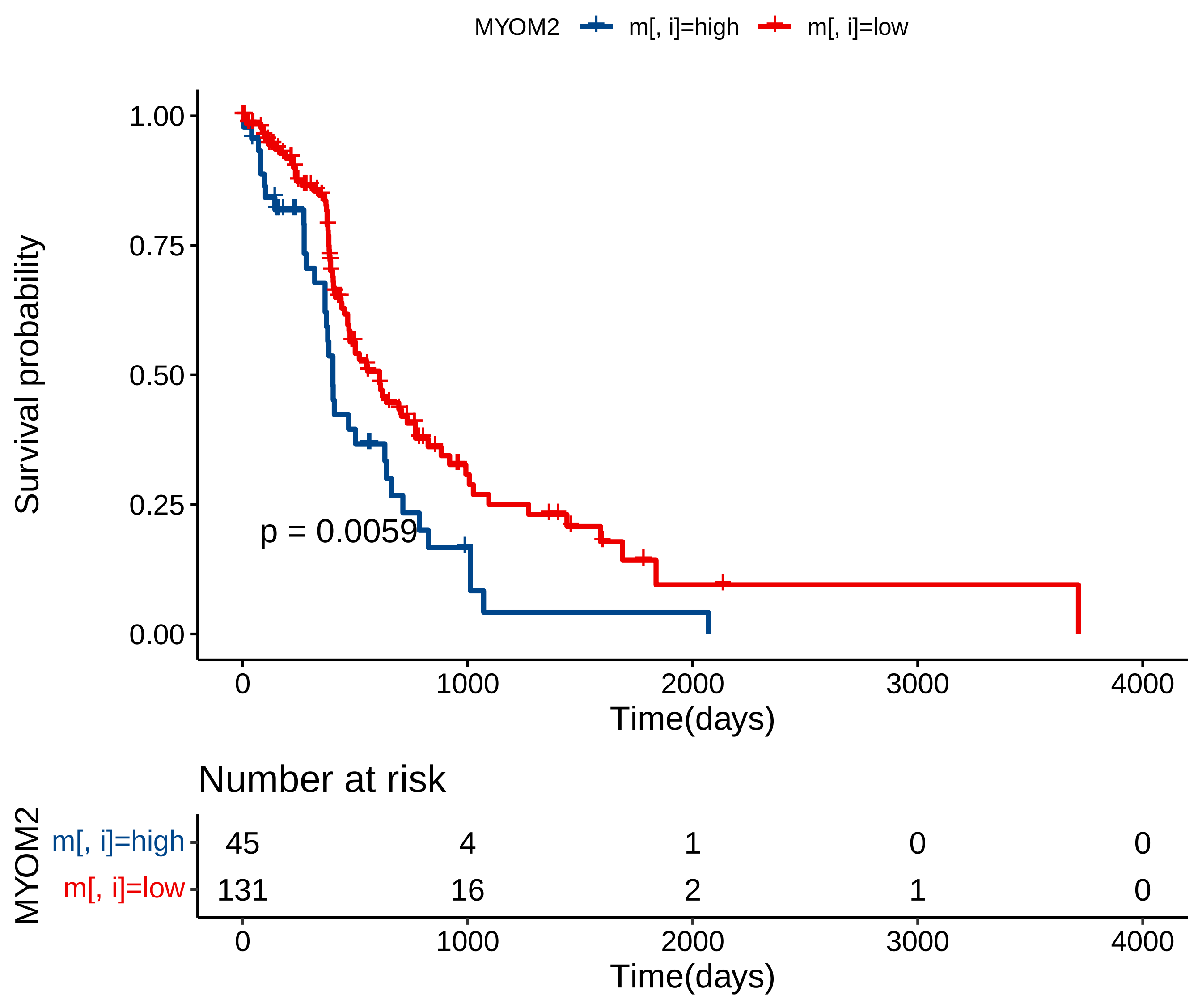
MYOM2
m[, i]=high
m[, i]=low
Survival probability
1.00
0.75
0.50
0.25
0.00
p = 0.0059
0
1000
2000
3000
4000
Time(days)
Number at risk
MYOM2
m[, i]=high
m[, i]=low
45 4 1 0 0
131 16 2 1 0
Time(days)

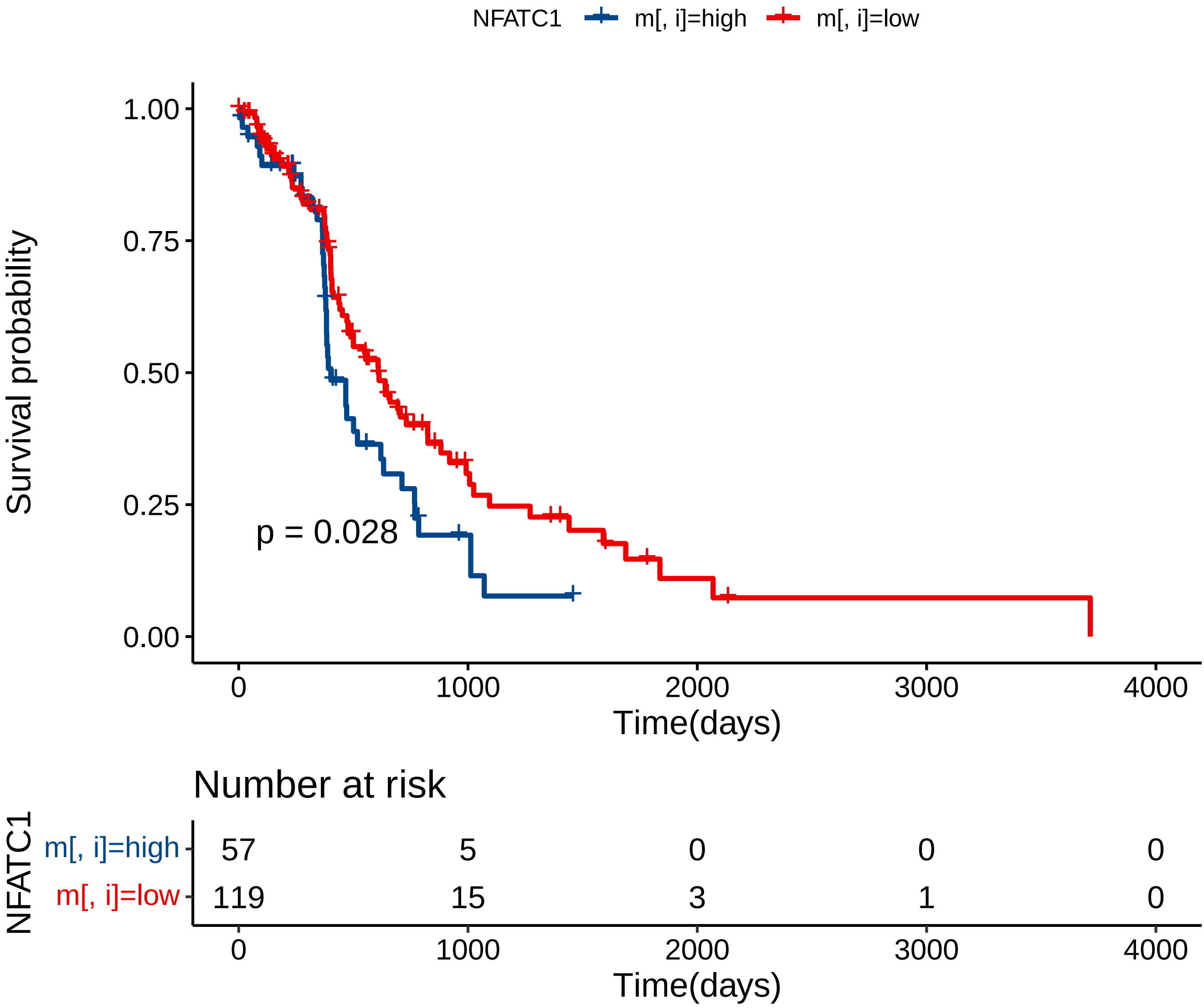
NFATC1
m[, i]=high
m[, i]=low
Survival probability
1.00
0.75
0.50
0.25
0.00
p = 0.028
0
1000
2000
3000
4000
Time(days)
Number at risk
NFATC1
m[, i]=high
m[, i]=low
57
5
0
0
0
119
15
3
1
0
0
1000
2000
3000
4000
Time(days)

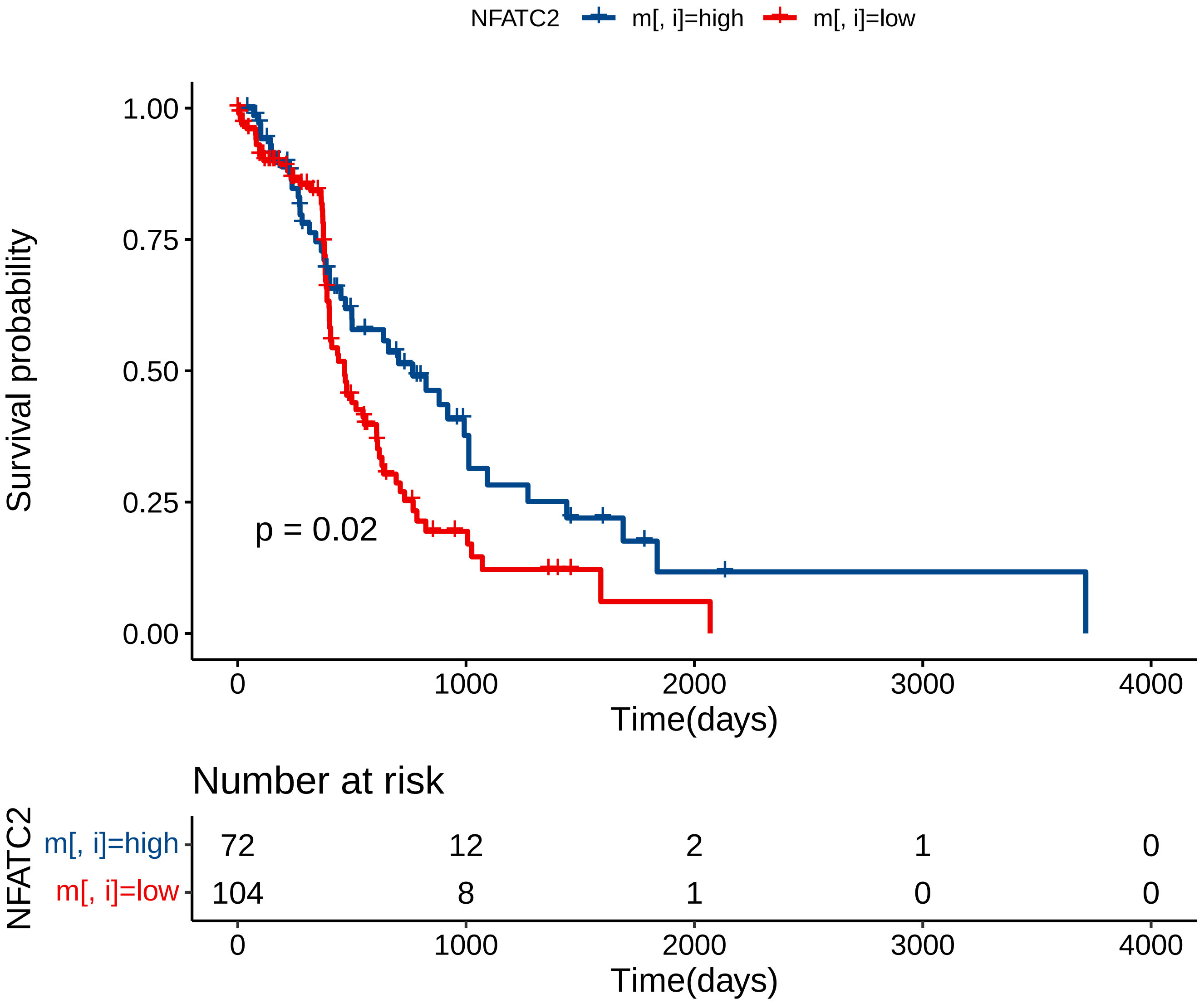
NFATC2
m[, i]=high
m[, i]=low
Survival probability
1.00
0.75
0.50
0.25
0.00
p = 0.02
0
1000
2000
3000
4000
Time(days)
Number at risk
NFATC2
m[, i]=high
m[, i]=low
72
12
2
1
0
104
8
1
0
0
0
1000
2000
3000
4000
Time(days)

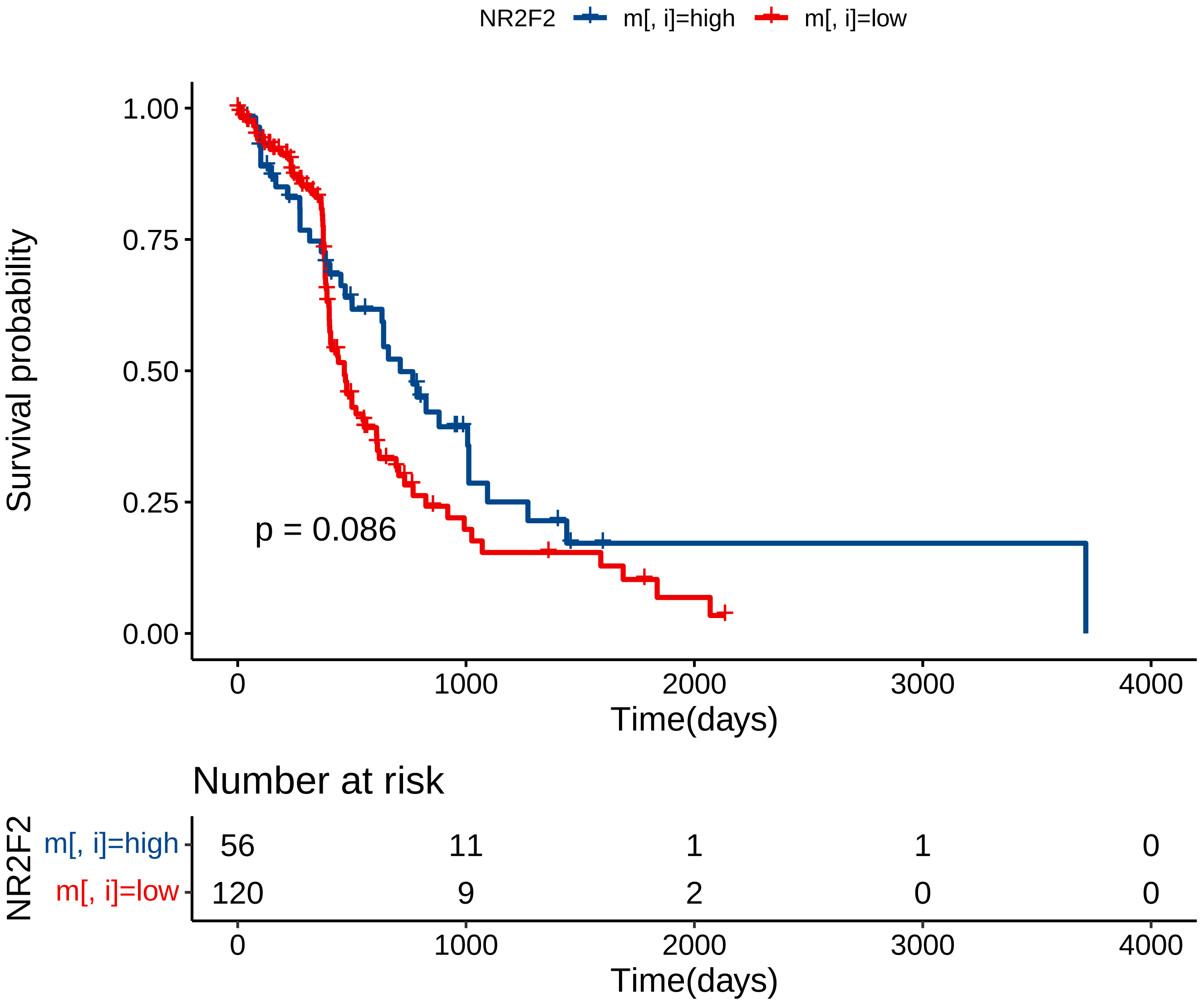
NR2F2
m[, i]=high
m[, i]=low
Survival probability
1.00
0.75
0.50
0.25
0.00
p = 0.086
0
1000
2000
3000
4000
Time(days)
Number at risk
NR2F2
m[, i]=high
m[, i]=low
56
11
1
1
0
120
9
2
0
0
0
1000
2000
3000
4000
Time(days)

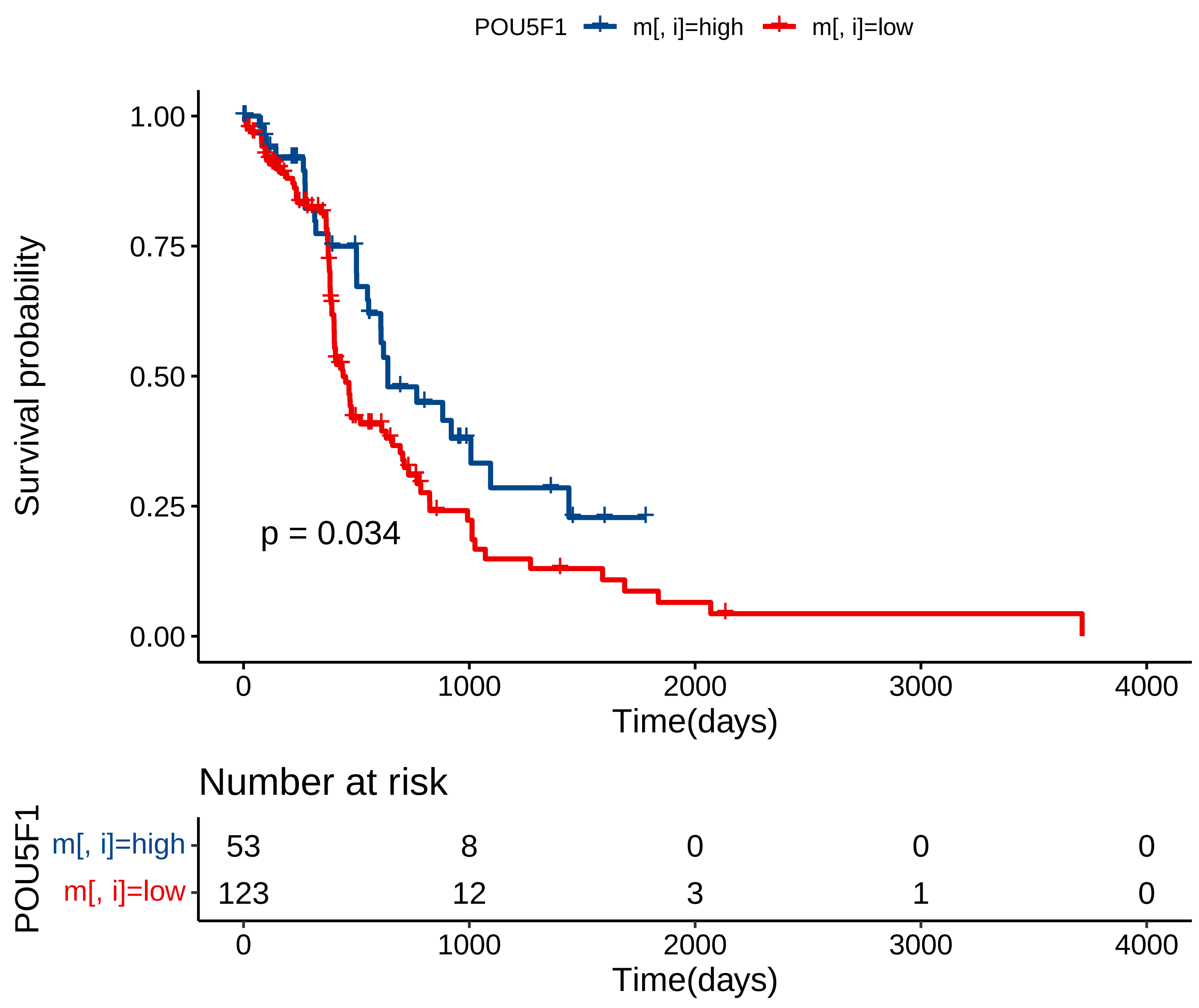
POU5F1
m[, i]=high
m[, i]=low
Survival probability
1.00
0.75
0.50
0.25
0.00
p = 0.034
0
1000
2000
3000
4000
Time(days)
Number at risk
POU5F1
m[, i]=high
m[, i]=low
53
8
0
0
0
123
12
3
1
0
0
1000
2000
3000
4000
Time(days)

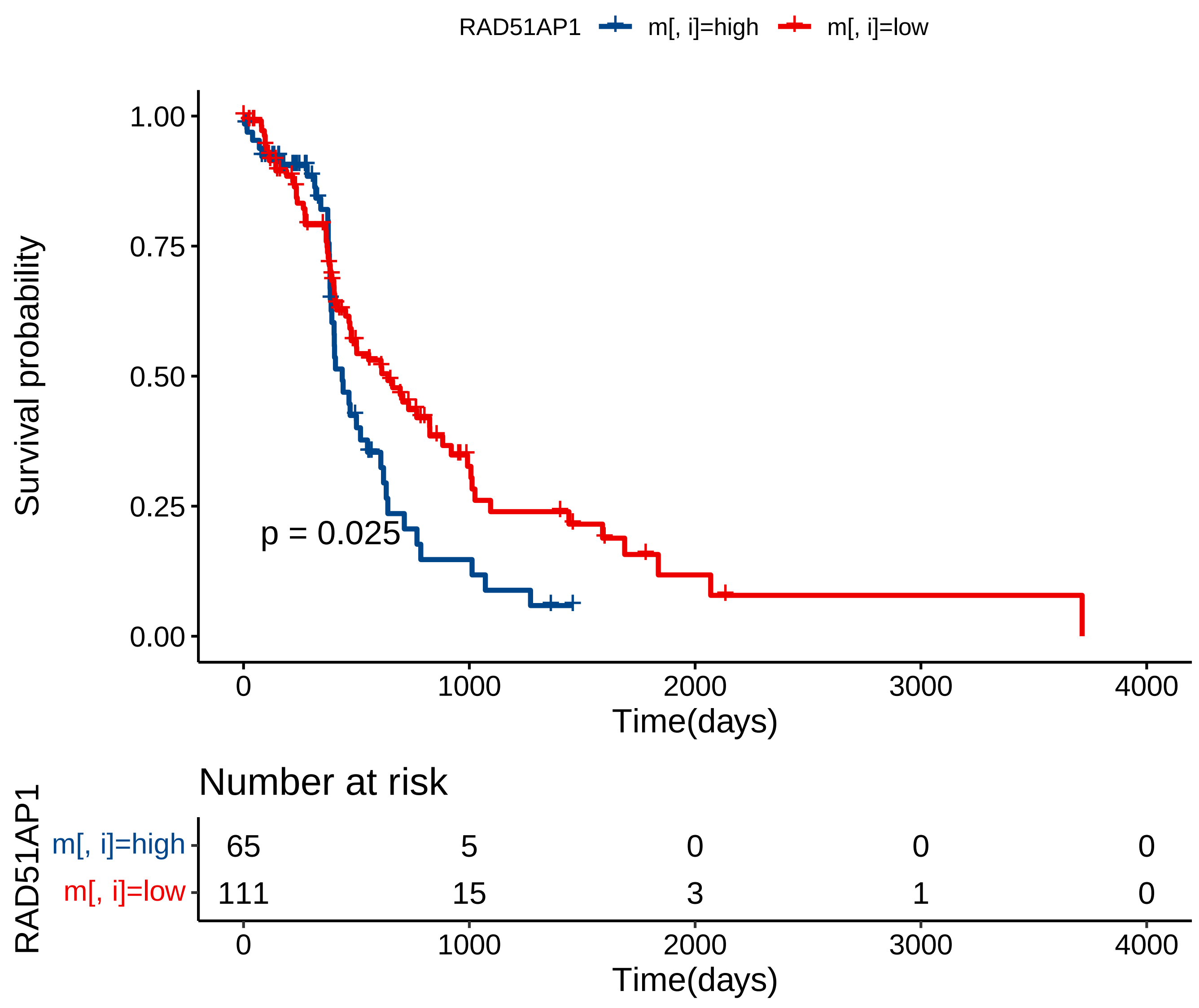
RAD51AP1
m[, i]=high
m[, i]=low
Survival probability
1.00
0.75
0.50
0.25
0.00
p = 0.025
0
1000
2000
3000
4000
Time(days)
Number at risk
RAD51AP1
m[, i]=high
m[, i]=low
65
5
0
0
0
111
15
3
1
0
0
1000
2000
3000
4000
Time(days)

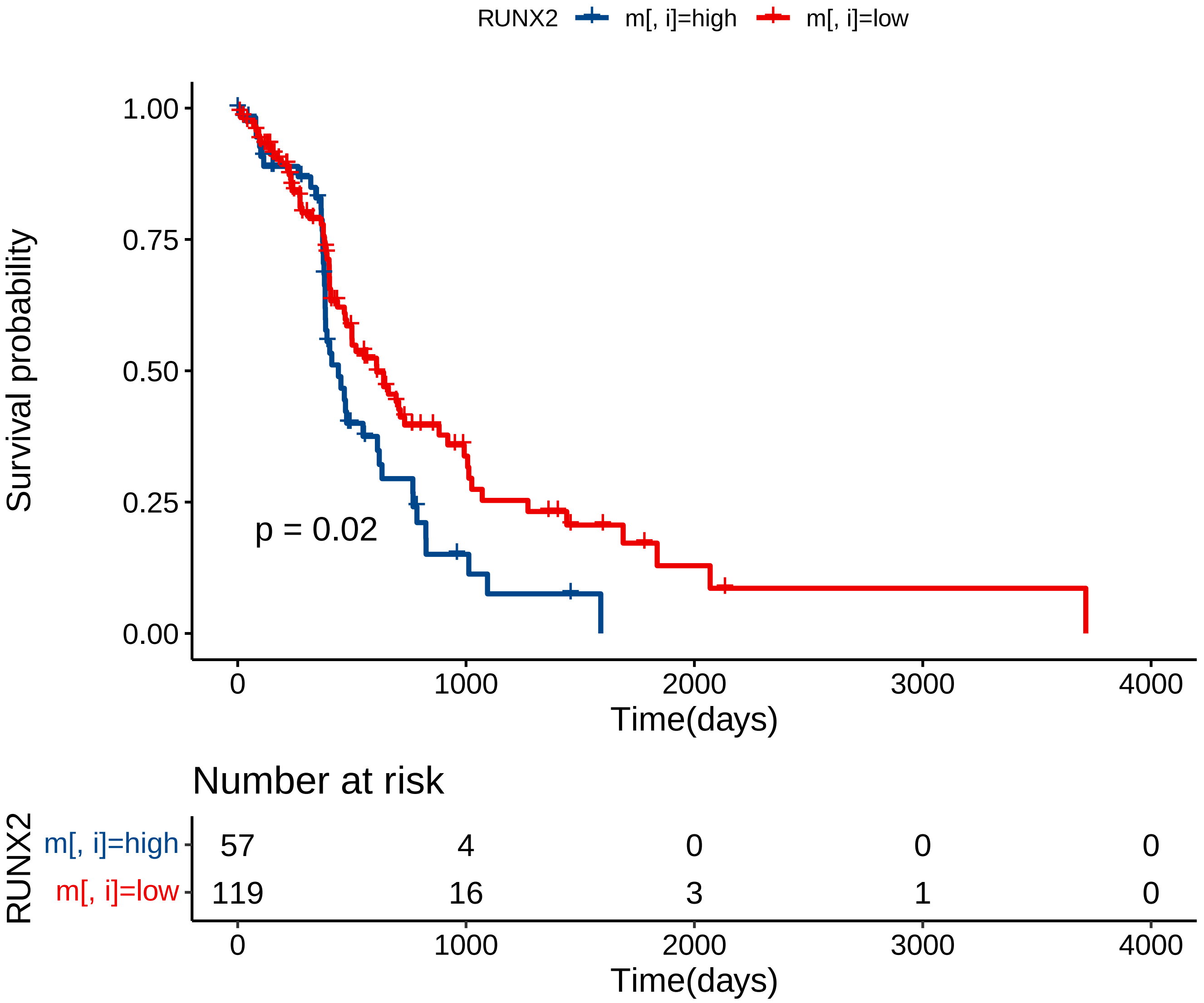

RUNX2
m[, i]=high
m[, i]=low
Survival probability
1.00
0.75
0.50
0.25
0.00
p = 0.02
0
1000
2000
3000
4000
Time(days)
Number at risk
RUNX2
m[, i]=high 57 4 0 0 0
m[, i]=low 119 16 3 1 0
0
1000
2000
3000
4000
Time(days)

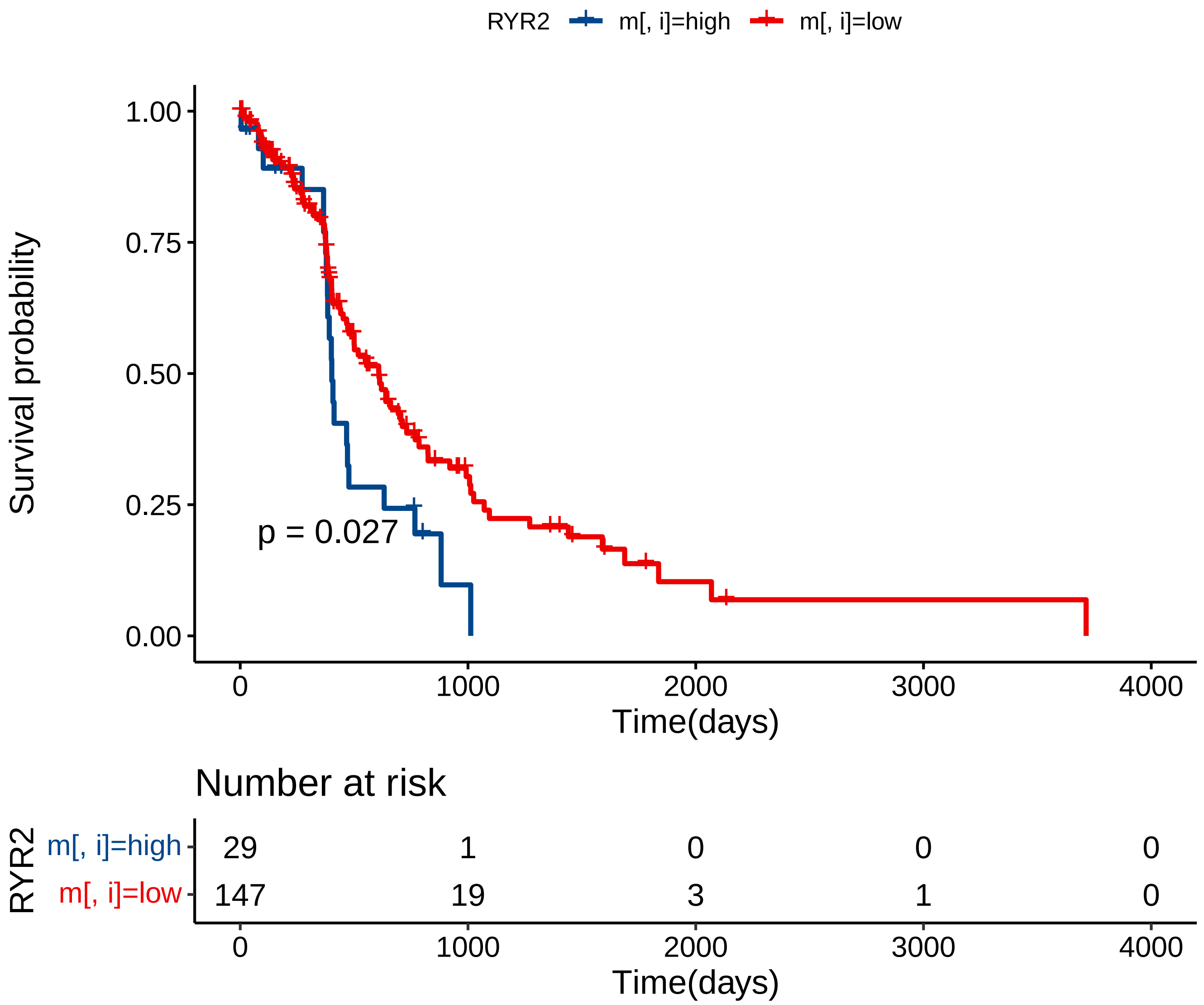
RYR2
m[, i]=high
m[, i]=low
Survival probability
1.00
0.75
0.50
0.25
0.00
p = 0.027
0
1000
2000
3000
4000
Time(days)
Number at risk
RYR2
m[, i]=high
m[, i]=low
29
1
0
0
0
147
19
3
1
0
0
1000
2000
3000
4000
Time(days)

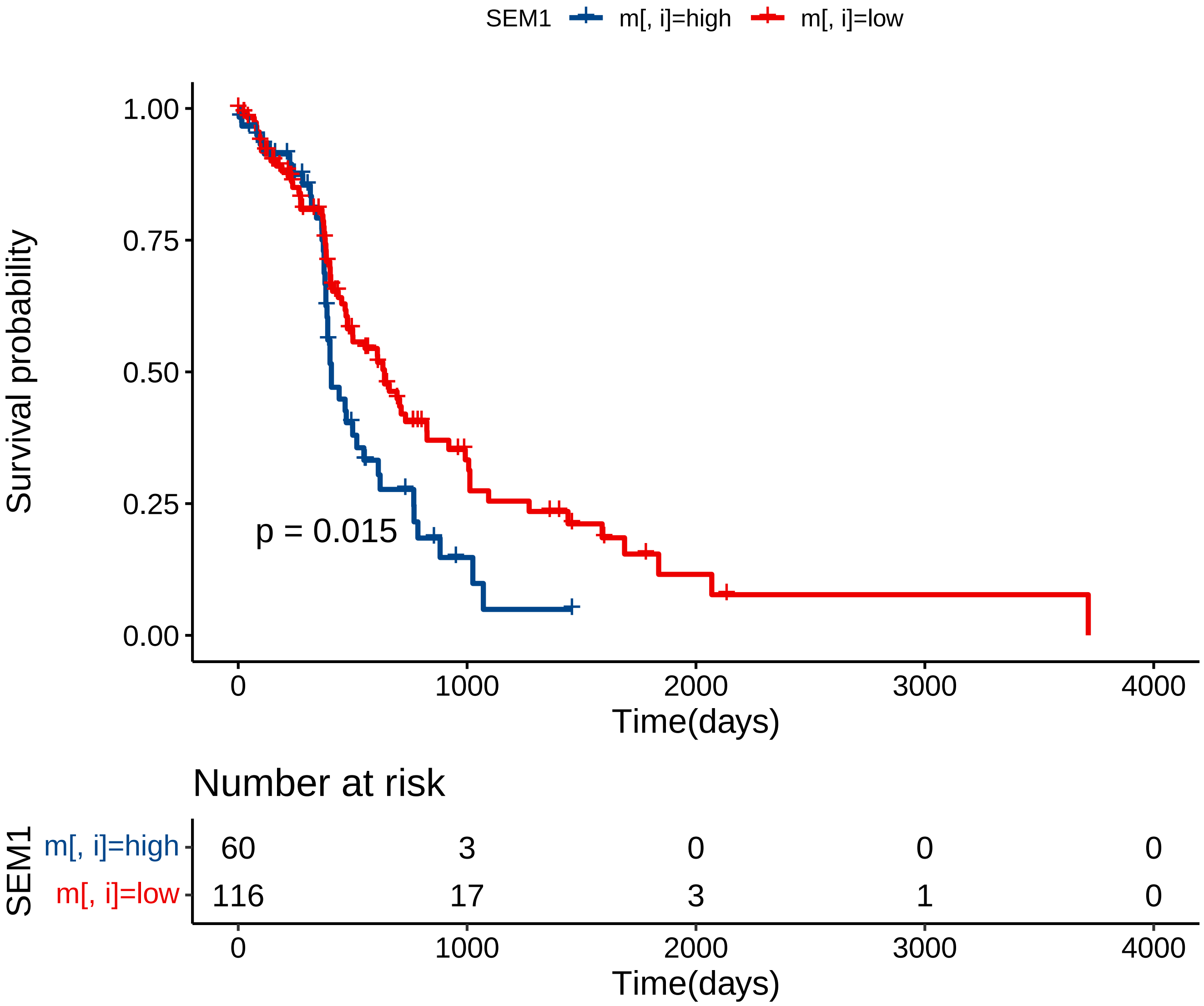
SEM1
m[, i]=high
m[, i]=low
Survival probability
1.00
0.75
0.50
0.25
0.00
p = 0.015
0
1000
2000
3000
4000
Time(days)
Number at risk
SEM1
m[, i]=high 60 3 0 0 0
m[, i]=low 116 17 3 1 0
0
1000
2000
3000
4000
Time(days)

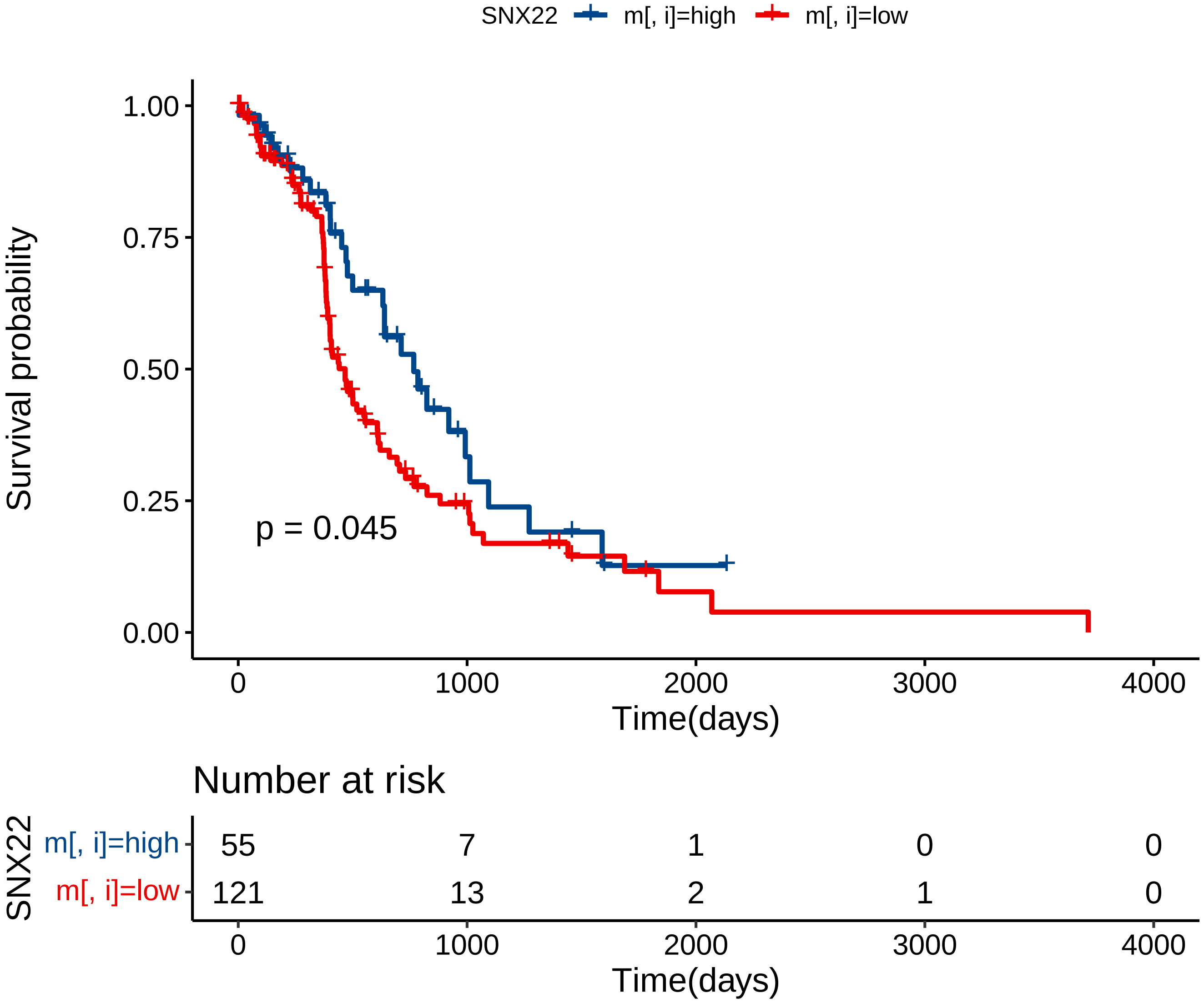

SNX22
m[, i]=high
m[, i]=low
Survival probability
1.00
0.75
0.50
0.25
0.00
p = 0.045
0
1000
2000
3000
4000
Time(days)
Number at risk
SNX22
m[, i]=high 55 7 1 0 0
m[, i]=low 121 13 2 1 0
0
1000
2000
3000
4000
Time(days)

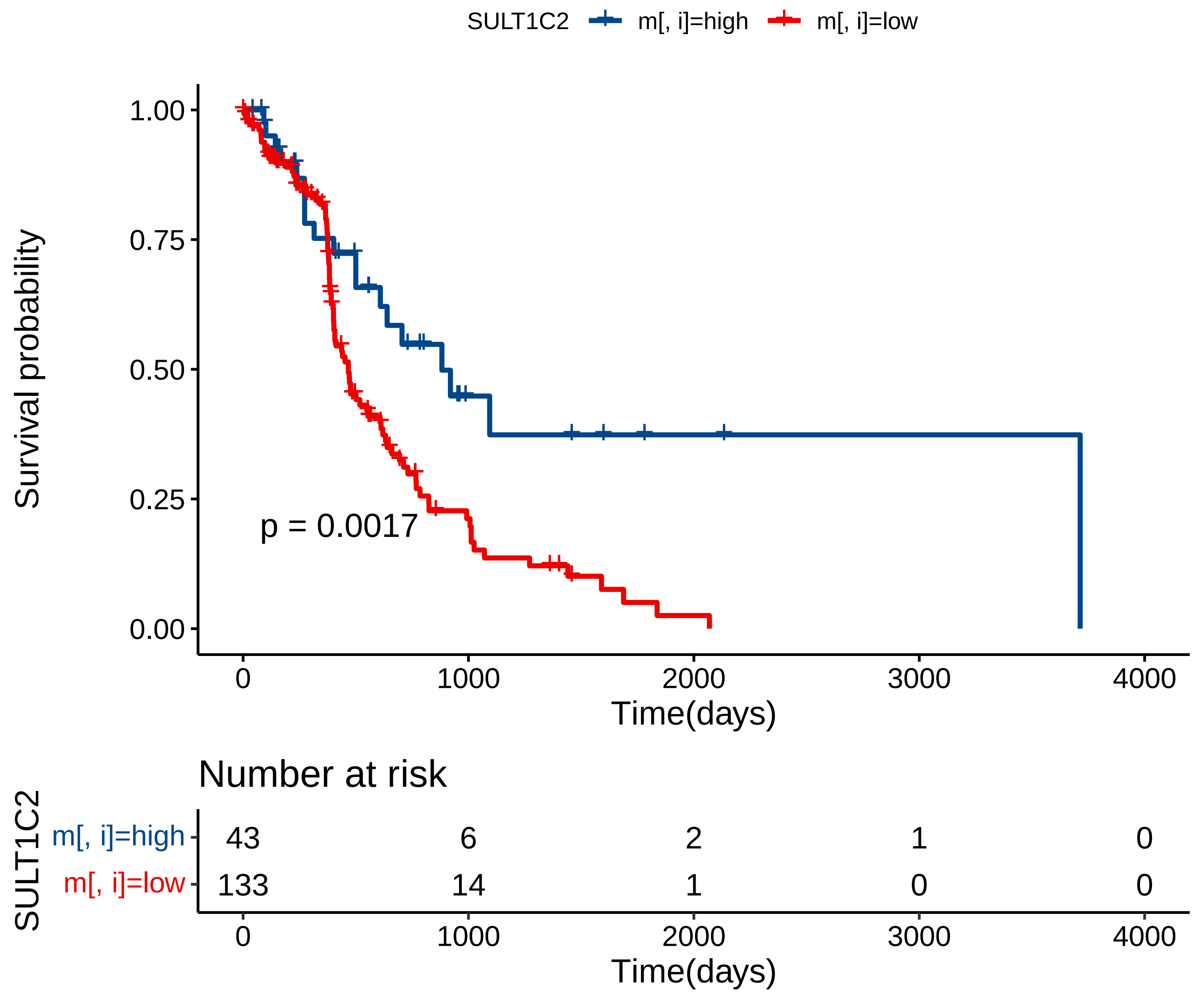

SULT1C2
m[, i]=high
m[, i]=low
Survival probability
1.00
0.75
0.50
0.25
0.00
p = 0.0017
0
1000
2000
3000
4000
Time(days)
Number at risk
SULT1C2
m[, i]=high 43 6 2 1 0
m[, i]=low 133 14 1 0 0
0
1000
2000
3000
4000
Time(days)

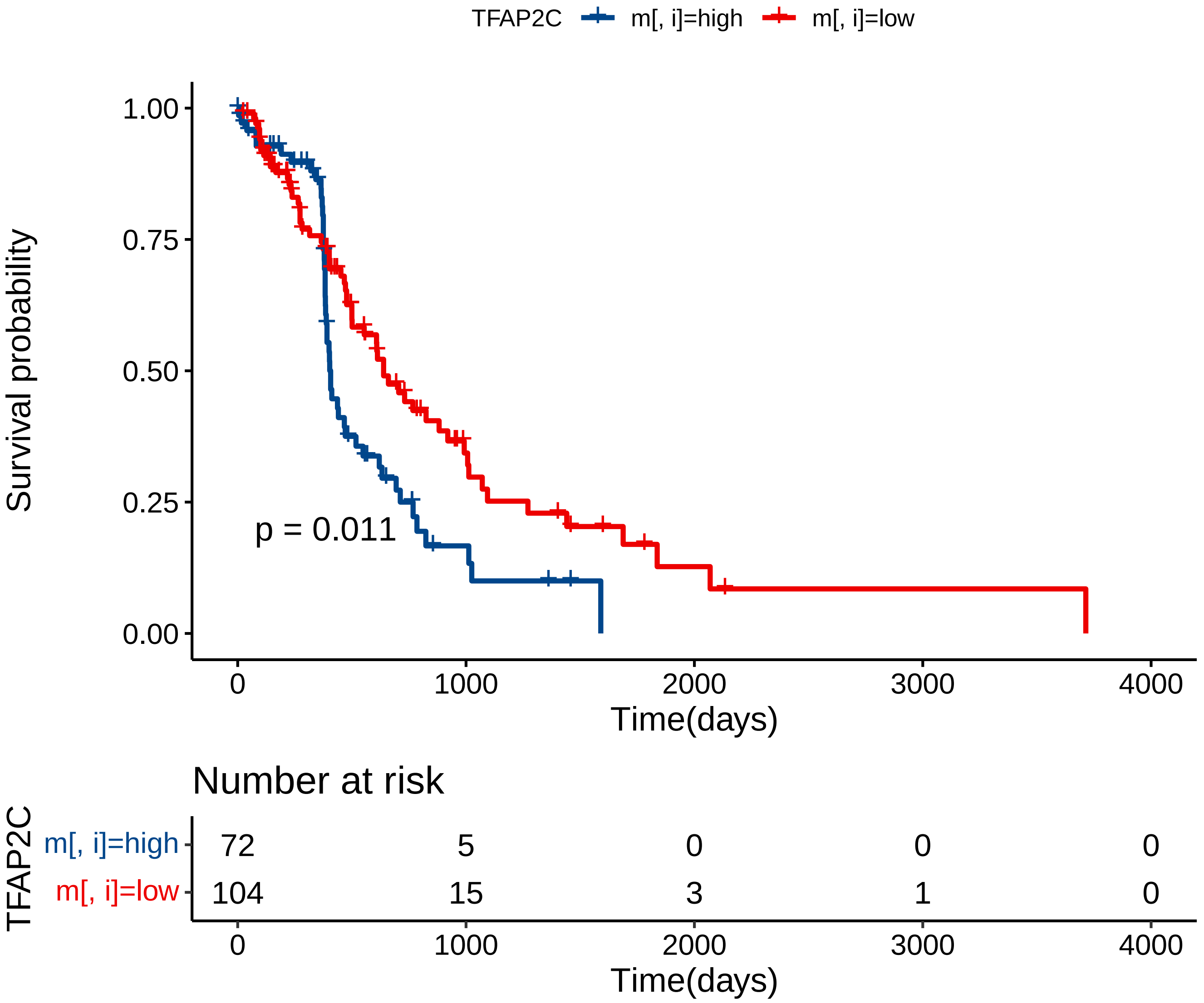
TFAP2C
m[, i]=high
m[, i]=low
1.00
0.75
0.50
0.25
0.00
Survival probability
p = 0.011
0
1000
2000
3000
4000
Time(days)
Number at risk
TFAP2C
m[, i]=high
m[, i]=low
72
5
0
0
0
104
15
3
1
0
0
1000
2000
3000
4000
Time(days)

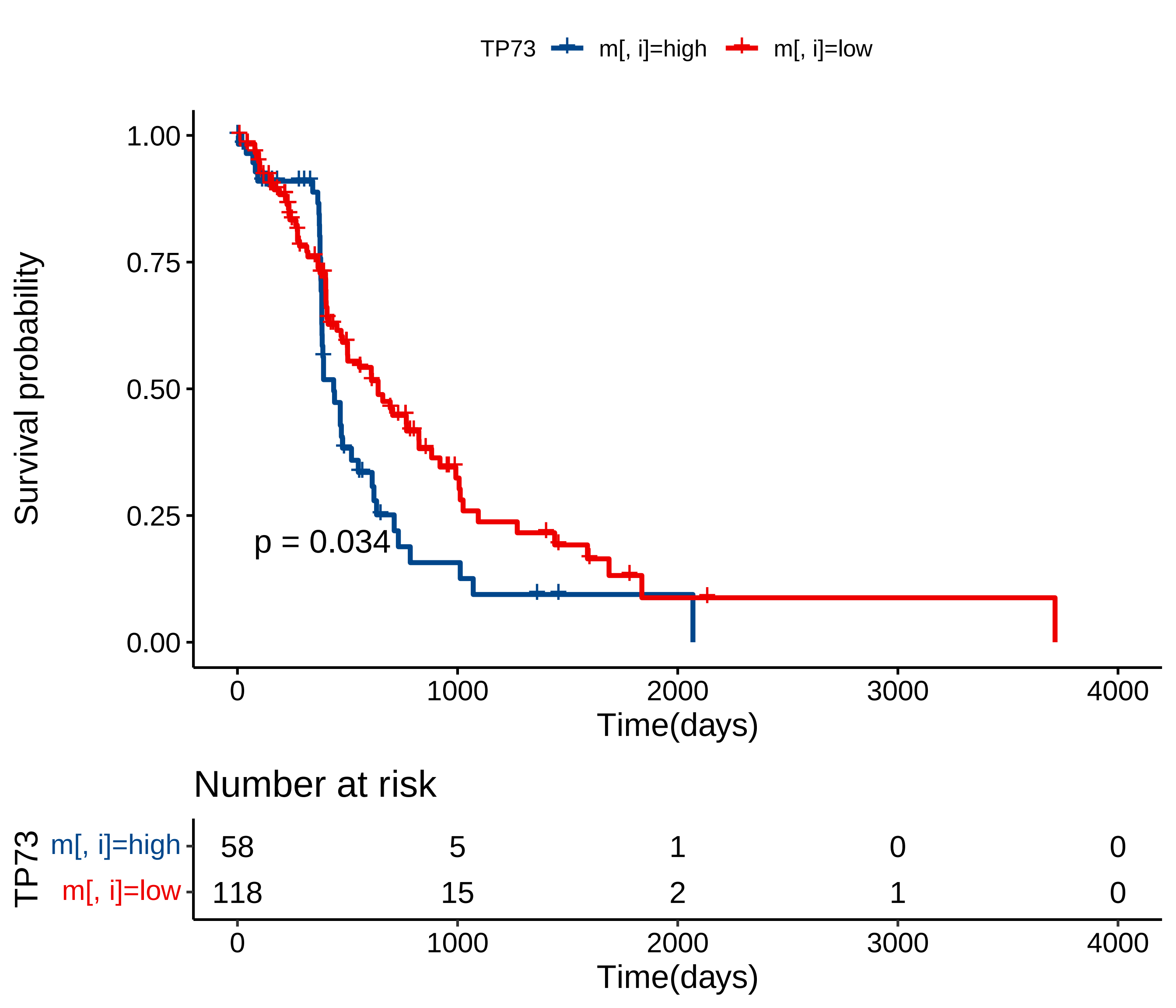
TP73
m[, i]=high
m[, i]=low
1.00
0.75
0.50
0.25
0.00
Survival probability
p = 0.034
0
1000
2000
3000
4000
Time(days)
Number at risk
TP73
m[, i]=high
m[, i]=low
58
5
1
0
0
118
15
2
1
0
0
1000
2000
3000
4000
Time(days)

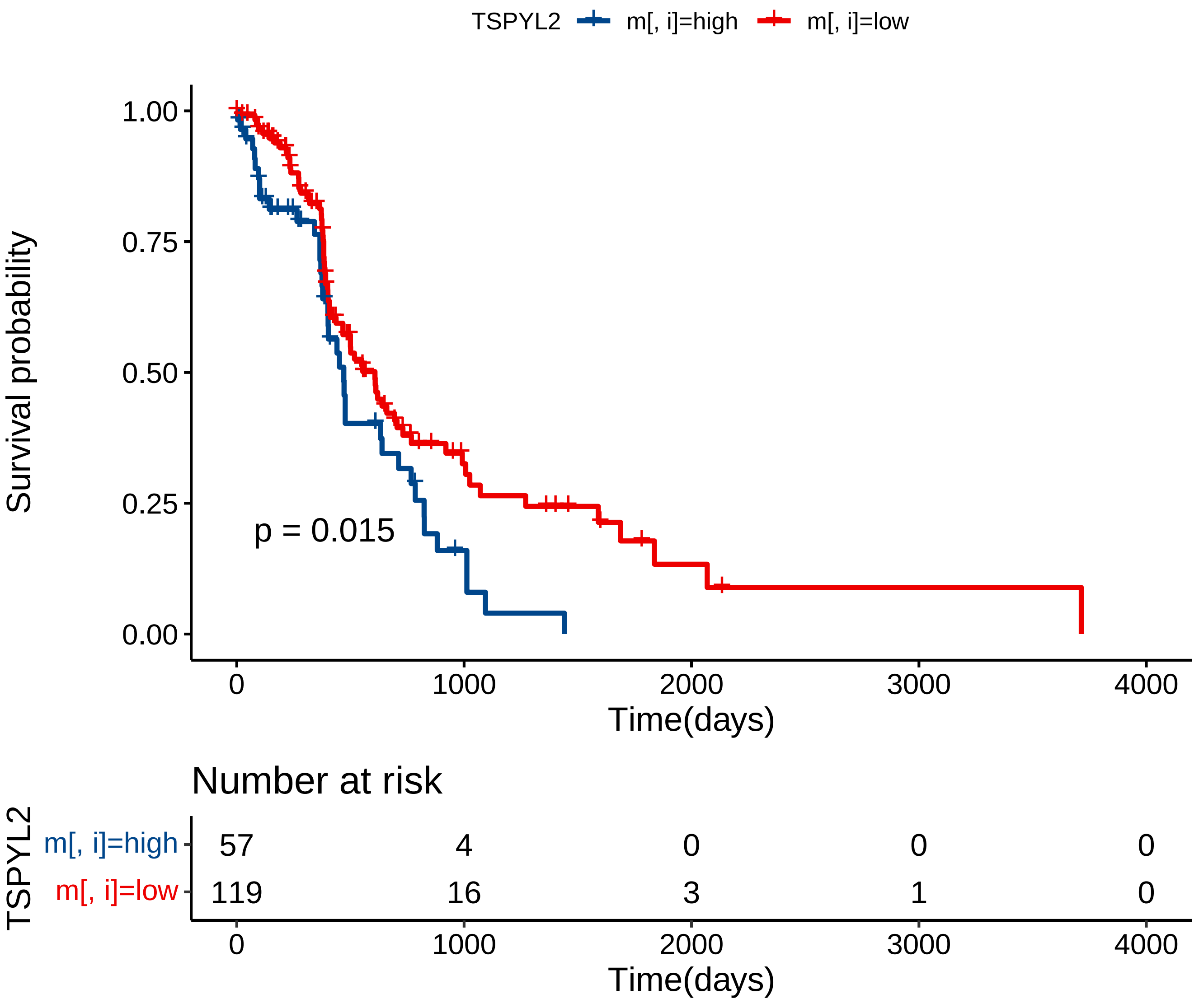
TSPYL2
m[, i]=high
m[, i]=low
Survival probability
1.00
0.75
0.50
0.25
0.00
p = 0.015
0
1000
2000
3000
4000
Time(days)
Number at risk
TSPYL2
m[, i]=high 57 4 0 0 0
m[, i]=low 119 16 3 1 0
0
1000
2000
3000
4000
Time(days)

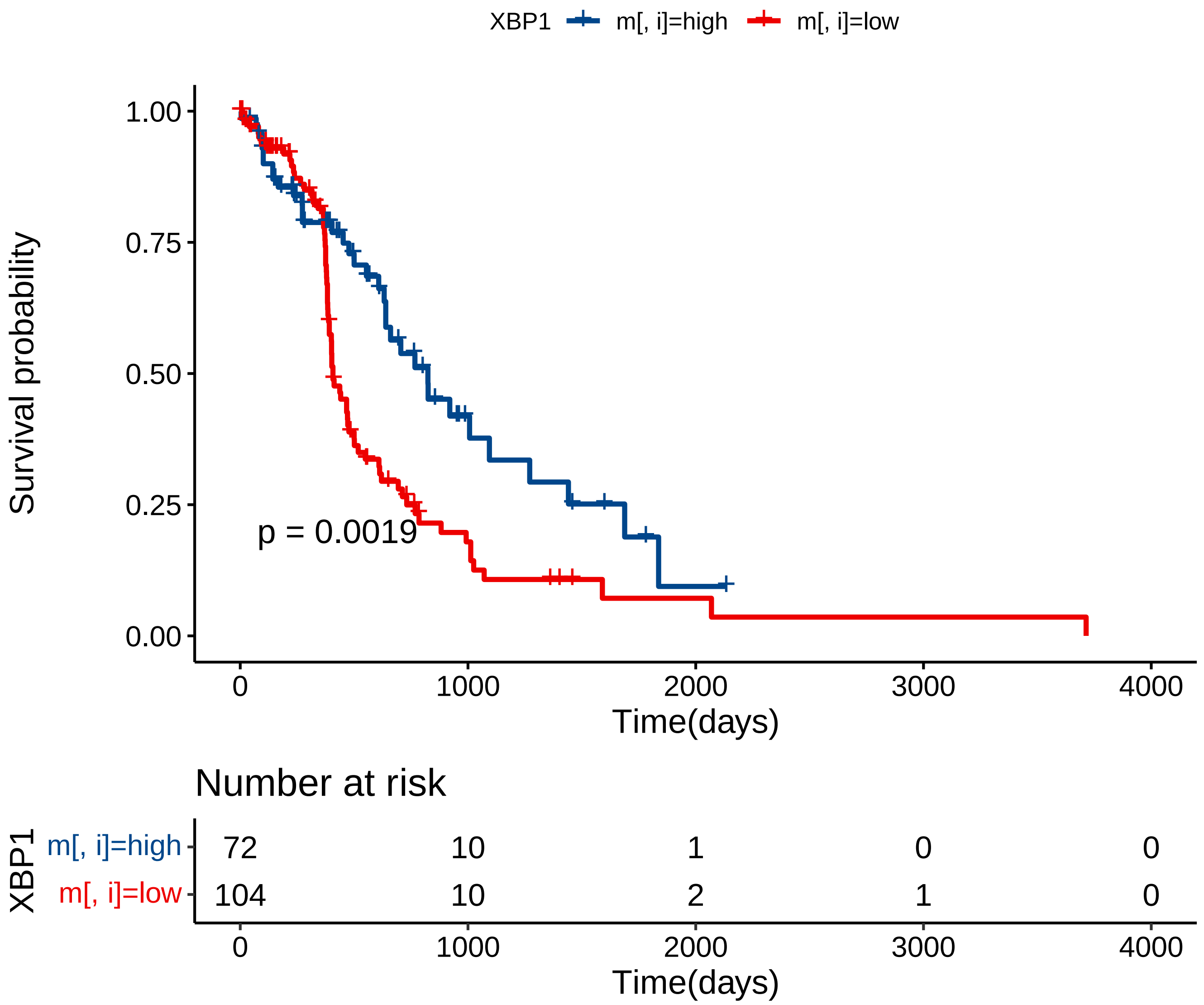

XBP1
m[, i]=high
m[, i]=low
Survival probability
1.00
0.75
0.50
0.25
0.00
p = 0.0019
0
1000
2000
3000
4000
Time(days)
Number at risk
XBP1
m[, i]=high 72 10 1 0 0
m[, i]=low 104 10 2 1 0
0
1000
2000
3000
4000
Time(days)
