# Supplementary figures and images for: Identification of MAPK12 as a Prognostic Biomarker for Esophageal Carcinoma Using Bioinformatics and Machine Learning
Source: Biomed Res Int. 2025 Dec 29;2025:2605071. doi: 10.1155/bmri/2605071 (PMC12746012; doi:10.1155/bmri/2605071)

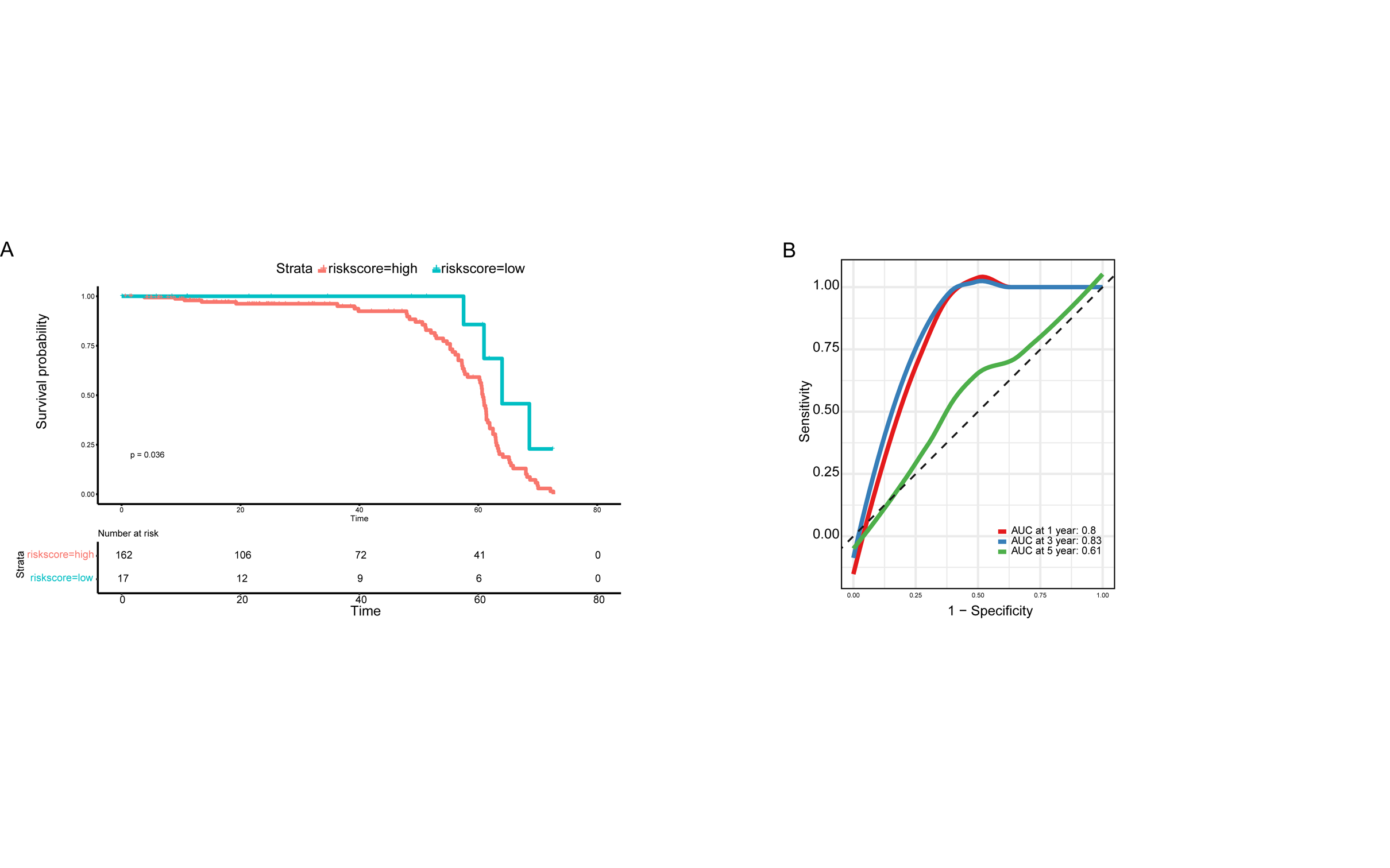

Supplement: Supplementary file 1 — Supporting Information Additional supporting information can be found online in the Supporting Information section.. Supplementary Figure S1: Validation of the prognostic signature model based on the GEO database. Supplementary Figure S2: The linkage between the GO results and each gene and the linkage between the GO results. Supplementary Figure S3: Bar chart (a) and heat map (b) displaying the difference in pathway activities enriched by GSVA between the high‐risk and low‐risk groups. Supplementary Figure S4: The estimate score between the high‐risk and low‐risk groups. Supplementary Figure S5: Full uncropped Gels and Blots images of MAPK12. Supplementary Figure S6: Full uncropped Gels and Blots images of E‐cadrenin. Supplementary Figure S7: Full uncropped Gels and Blots images of N‐cadrenin. Supplementary Table S1: Clinical characteristics of patients with ESCA in the training datasets (TCGA) and the validation datasets (GEO). Supplementary Table S2: List of Tolement‐related genes included in the present study. Supplementary Table S3: List of differentially expressed genes (DEGs) between ESCA and normal lung tissues based on the TCGA database. Supplementary Table S4: List of 265 Tolement‐related DEGs by taking the intersection of DEGs of ESCA and Tolement‐related genes. Supplementary Table S5: 33 candidate genes with prognostic values were screened out by the Kaplan–Meier survival analysis. Supplementary Appendix 1. The detailed protocols for cell culture, transfection, and functional assays. Supplementary Appendix 2: The detailed statistical methods. [file BMRI-2025-2605071-s001.zip › Supplementary Figure 1.tif]

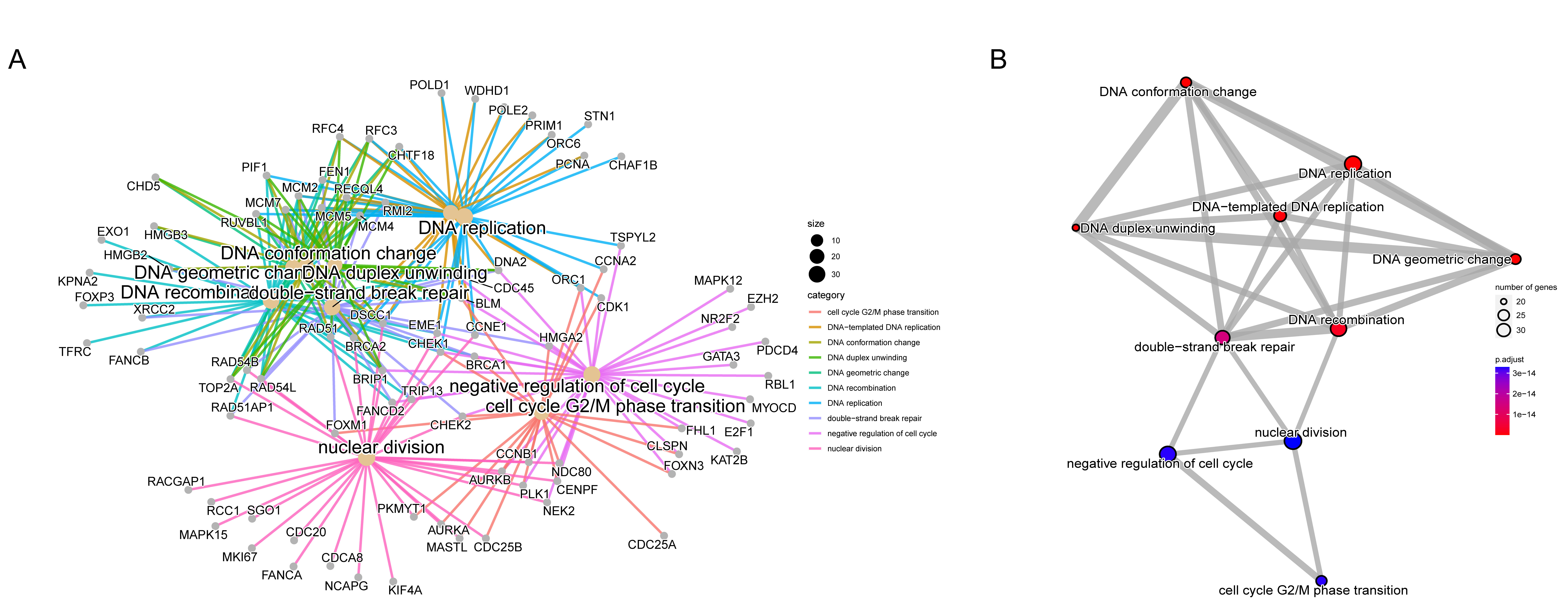

Supplement: Supplementary file 1 — Supporting Information Additional supporting information can be found online in the Supporting Information section.. Supplementary Figure S1: Validation of the prognostic signature model based on the GEO database. Supplementary Figure S2: The linkage between the GO results and each gene and the linkage between the GO results. Supplementary Figure S3: Bar chart (a) and heat map (b) displaying the difference in pathway activities enriched by GSVA between the high‐risk and low‐risk groups. Supplementary Figure S4: The estimate score between the high‐risk and low‐risk groups. Supplementary Figure S5: Full uncropped Gels and Blots images of MAPK12. Supplementary Figure S6: Full uncropped Gels and Blots images of E‐cadrenin. Supplementary Figure S7: Full uncropped Gels and Blots images of N‐cadrenin. Supplementary Table S1: Clinical characteristics of patients with ESCA in the training datasets (TCGA) and the validation datasets (GEO). Supplementary Table S2: List of Tolement‐related genes included in the present study. Supplementary Table S3: List of differentially expressed genes (DEGs) between ESCA and normal lung tissues based on the TCGA database. Supplementary Table S4: List of 265 Tolement‐related DEGs by taking the intersection of DEGs of ESCA and Tolement‐related genes. Supplementary Table S5: 33 candidate genes with prognostic values were screened out by the Kaplan–Meier survival analysis. Supplementary Appendix 1. The detailed protocols for cell culture, transfection, and functional assays. Supplementary Appendix 2: The detailed statistical methods. [file BMRI-2025-2605071-s001.zip › Supplementary Figure 2.tif]

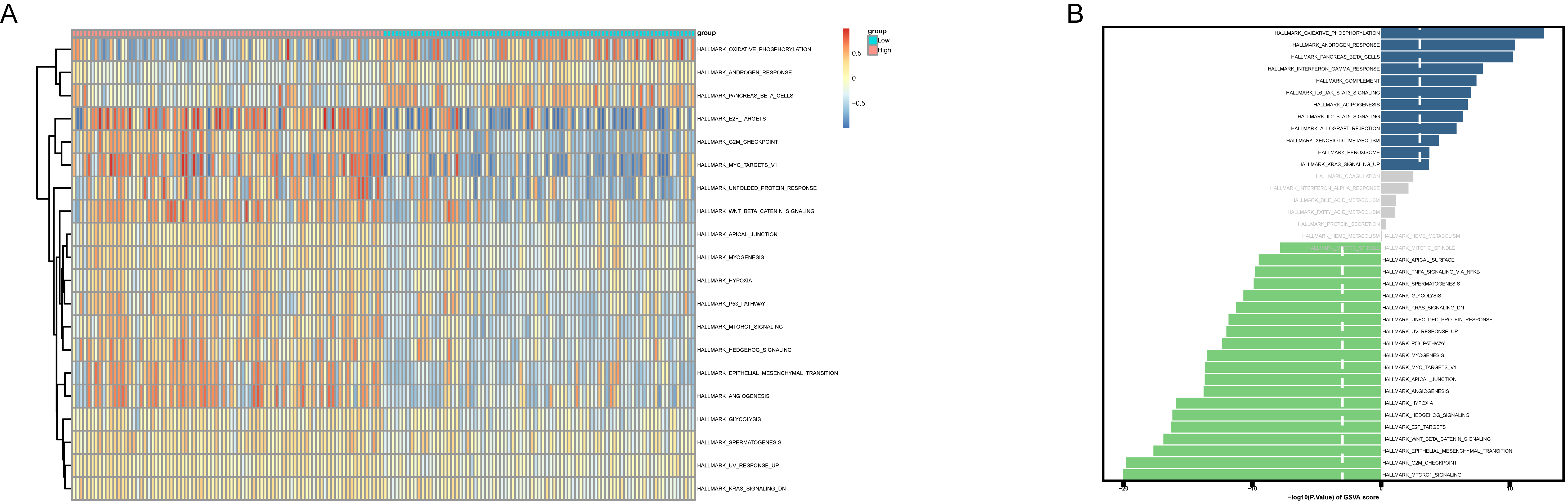

Supplement: Supplementary file 1 — Supporting Information Additional supporting information can be found online in the Supporting Information section.. Supplementary Figure S1: Validation of the prognostic signature model based on the GEO database. Supplementary Figure S2: The linkage between the GO results and each gene and the linkage between the GO results. Supplementary Figure S3: Bar chart (a) and heat map (b) displaying the difference in pathway activities enriched by GSVA between the high‐risk and low‐risk groups. Supplementary Figure S4: The estimate score between the high‐risk and low‐risk groups. Supplementary Figure S5: Full uncropped Gels and Blots images of MAPK12. Supplementary Figure S6: Full uncropped Gels and Blots images of E‐cadrenin. Supplementary Figure S7: Full uncropped Gels and Blots images of N‐cadrenin. Supplementary Table S1: Clinical characteristics of patients with ESCA in the training datasets (TCGA) and the validation datasets (GEO). Supplementary Table S2: List of Tolement‐related genes included in the present study. Supplementary Table S3: List of differentially expressed genes (DEGs) between ESCA and normal lung tissues based on the TCGA database. Supplementary Table S4: List of 265 Tolement‐related DEGs by taking the intersection of DEGs of ESCA and Tolement‐related genes. Supplementary Table S5: 33 candidate genes with prognostic values were screened out by the Kaplan–Meier survival analysis. Supplementary Appendix 1. The detailed protocols for cell culture, transfection, and functional assays. Supplementary Appendix 2: The detailed statistical methods. [file BMRI-2025-2605071-s001.zip › Supplementary Figure 3.tif]

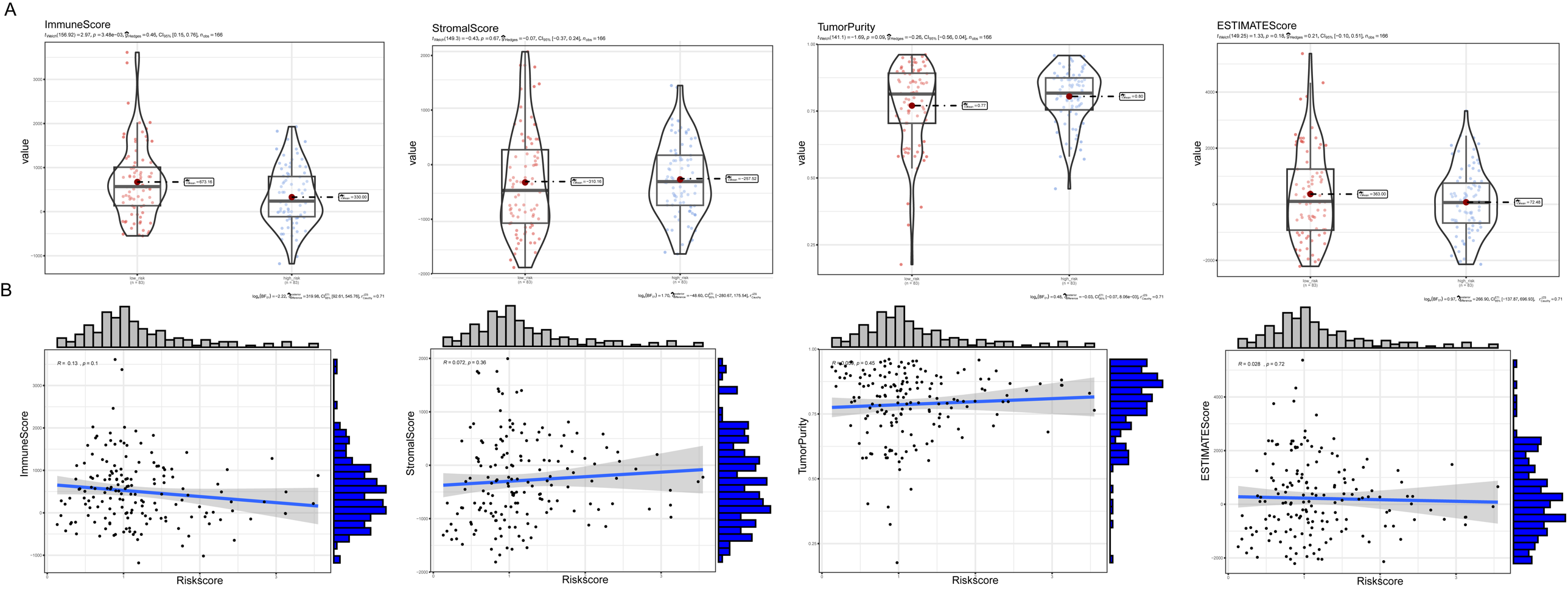

Supplement: Supplementary file 1 — Supporting Information Additional supporting information can be found online in the Supporting Information section.. Supplementary Figure S1: Validation of the prognostic signature model based on the GEO database. Supplementary Figure S2: The linkage between the GO results and each gene and the linkage between the GO results. Supplementary Figure S3: Bar chart (a) and heat map (b) displaying the difference in pathway activities enriched by GSVA between the high‐risk and low‐risk groups. Supplementary Figure S4: The estimate score between the high‐risk and low‐risk groups. Supplementary Figure S5: Full uncropped Gels and Blots images of MAPK12. Supplementary Figure S6: Full uncropped Gels and Blots images of E‐cadrenin. Supplementary Figure S7: Full uncropped Gels and Blots images of N‐cadrenin. Supplementary Table S1: Clinical characteristics of patients with ESCA in the training datasets (TCGA) and the validation datasets (GEO). Supplementary Table S2: List of Tolement‐related genes included in the present study. Supplementary Table S3: List of differentially expressed genes (DEGs) between ESCA and normal lung tissues based on the TCGA database. Supplementary Table S4: List of 265 Tolement‐related DEGs by taking the intersection of DEGs of ESCA and Tolement‐related genes. Supplementary Table S5: 33 candidate genes with prognostic values were screened out by the Kaplan–Meier survival analysis. Supplementary Appendix 1. The detailed protocols for cell culture, transfection, and functional assays. Supplementary Appendix 2: The detailed statistical methods. [file BMRI-2025-2605071-s001.zip › Supplementary Figure 4.tif]

ECA109

MAPK12

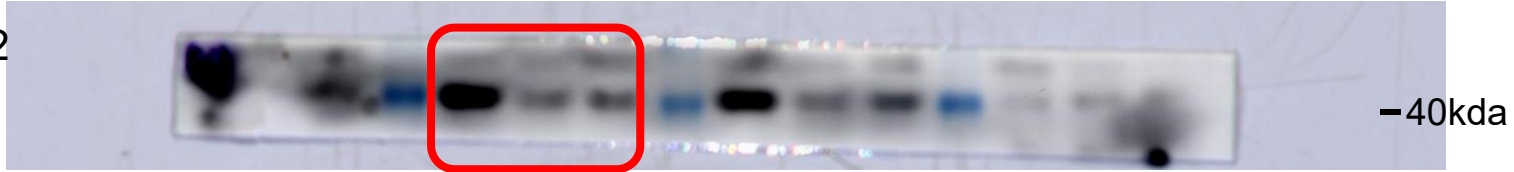

TUBLIN

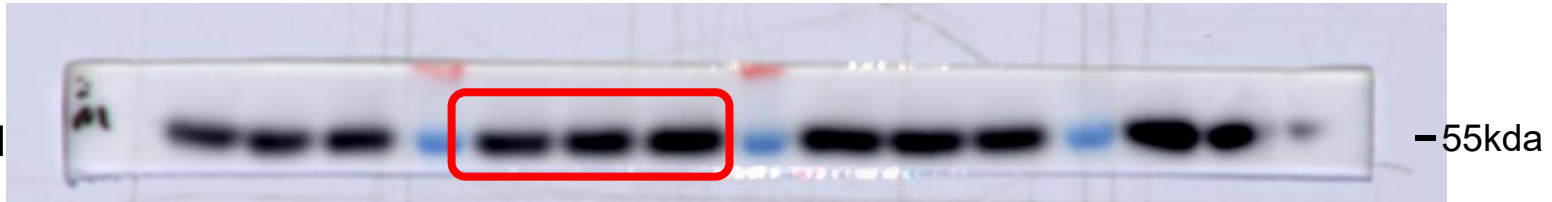

KESE180

MAPK12

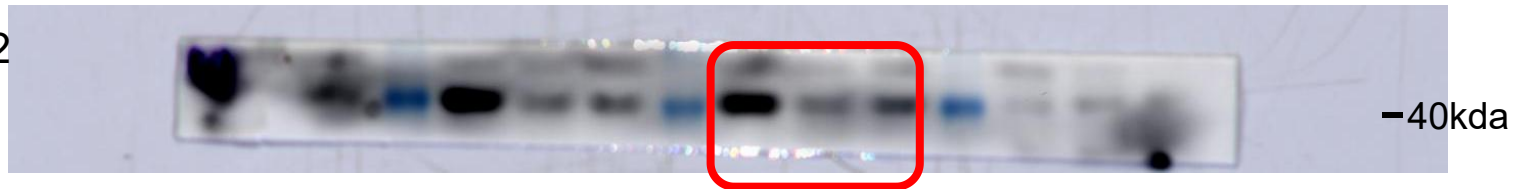

TUBLIN

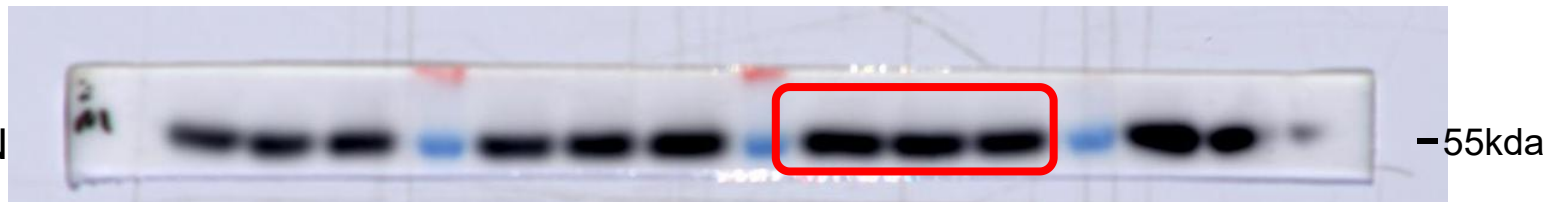

Supplement: Supplementary file 1 — Supporting Information Additional supporting information can be found online in the Supporting Information section.. Supplementary Figure S1: Validation of the prognostic signature model based on the GEO database. Supplementary Figure S2: The linkage between the GO results and each gene and the linkage between the GO results. Supplementary Figure S3: Bar chart (a) and heat map (b) displaying the difference in pathway activities enriched by GSVA between the high‐risk and low‐risk groups. Supplementary Figure S4: The estimate score between the high‐risk and low‐risk groups. Supplementary Figure S5: Full uncropped Gels and Blots images of MAPK12. Supplementary Figure S6: Full uncropped Gels and Blots images of E‐cadrenin. Supplementary Figure S7: Full uncropped Gels and Blots images of N‐cadrenin. Supplementary Table S1: Clinical characteristics of patients with ESCA in the training datasets (TCGA) and the validation datasets (GEO). Supplementary Table S2: List of Tolement‐related genes included in the present study. Supplementary Table S3: List of differentially expressed genes (DEGs) between ESCA and normal lung tissues based on the TCGA database. Supplementary Table S4: List of 265 Tolement‐related DEGs by taking the intersection of DEGs of ESCA and Tolement‐related genes. Supplementary Table S5: 33 candidate genes with prognostic values were screened out by the Kaplan–Meier survival analysis. Supplementary Appendix 1. The detailed protocols for cell culture, transfection, and functional assays. Supplementary Appendix 2: The detailed statistical methods. [file BMRI-2025-2605071-s001.zip › Supplementary Figure 5.pdf]

## KESE180

E-cadrenin

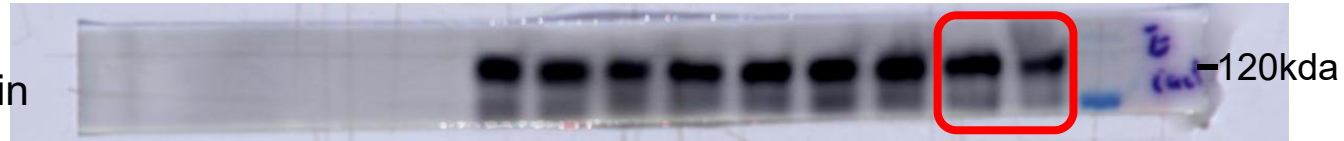

$\beta$ -Actin

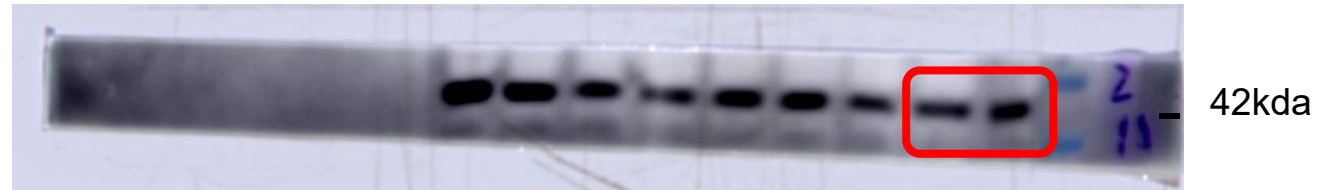

## ECA109

E-cadrenin

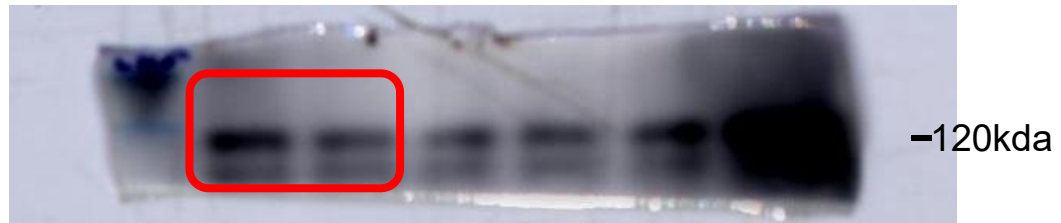

$\beta$ -Actin

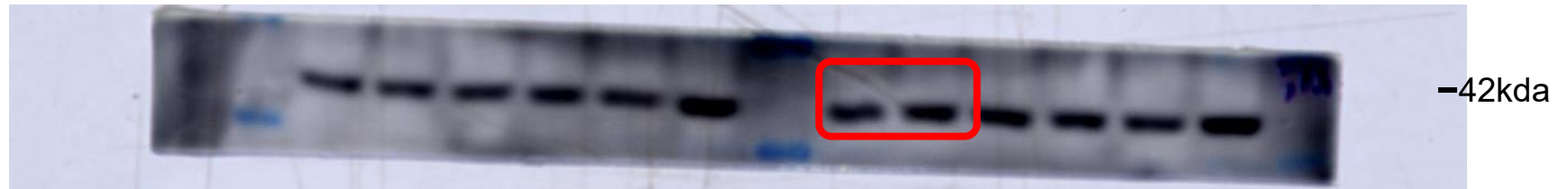

Supplement: Supplementary file 1 — Supporting Information Additional supporting information can be found online in the Supporting Information section.. Supplementary Figure S1: Validation of the prognostic signature model based on the GEO database. Supplementary Figure S2: The linkage between the GO results and each gene and the linkage between the GO results. Supplementary Figure S3: Bar chart (a) and heat map (b) displaying the difference in pathway activities enriched by GSVA between the high‐risk and low‐risk groups. Supplementary Figure S4: The estimate score between the high‐risk and low‐risk groups. Supplementary Figure S5: Full uncropped Gels and Blots images of MAPK12. Supplementary Figure S6: Full uncropped Gels and Blots images of E‐cadrenin. Supplementary Figure S7: Full uncropped Gels and Blots images of N‐cadrenin. Supplementary Table S1: Clinical characteristics of patients with ESCA in the training datasets (TCGA) and the validation datasets (GEO). Supplementary Table S2: List of Tolement‐related genes included in the present study. Supplementary Table S3: List of differentially expressed genes (DEGs) between ESCA and normal lung tissues based on the TCGA database. Supplementary Table S4: List of 265 Tolement‐related DEGs by taking the intersection of DEGs of ESCA and Tolement‐related genes. Supplementary Table S5: 33 candidate genes with prognostic values were screened out by the Kaplan–Meier survival analysis. Supplementary Appendix 1. The detailed protocols for cell culture, transfection, and functional assays. Supplementary Appendix 2: The detailed statistical methods. [file BMRI-2025-2605071-s001.zip › Supplementary Figure 6.pdf]

KESE180

N-cadrenin

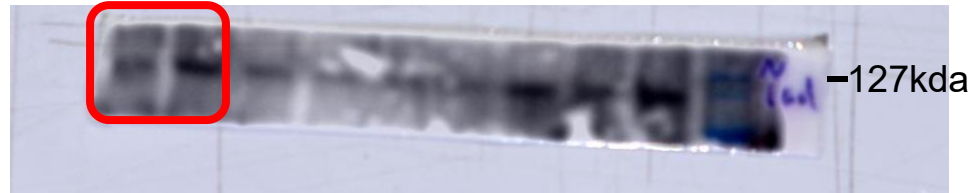

$\beta$ -Actin

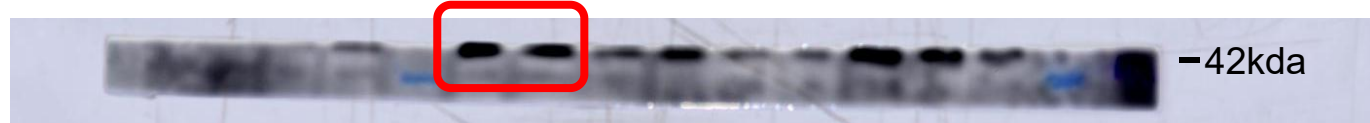

ECA109

N-cadrenin

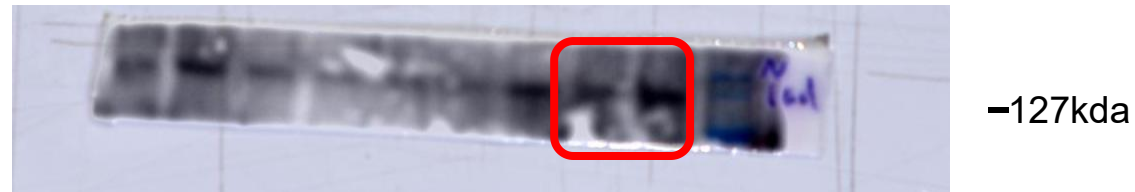

$\beta$ -Actin

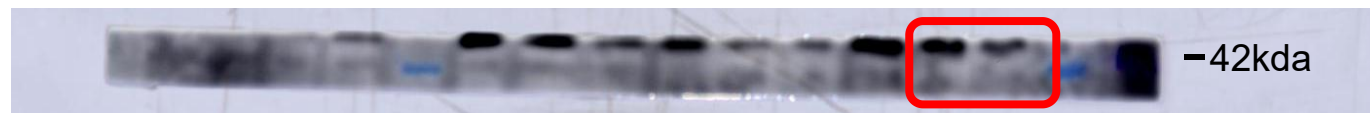

Supplement: Supplementary file 1 — Supporting Information Additional supporting information can be found online in the Supporting Information section.. Supplementary Figure S1: Validation of the prognostic signature model based on the GEO database. Supplementary Figure S2: The linkage between the GO results and each gene and the linkage between the GO results. Supplementary Figure S3: Bar chart (a) and heat map (b) displaying the difference in pathway activities enriched by GSVA between the high‐risk and low‐risk groups. Supplementary Figure S4: The estimate score between the high‐risk and low‐risk groups. Supplementary Figure S5: Full uncropped Gels and Blots images of MAPK12. Supplementary Figure S6: Full uncropped Gels and Blots images of E‐cadrenin. Supplementary Figure S7: Full uncropped Gels and Blots images of N‐cadrenin. Supplementary Table S1: Clinical characteristics of patients with ESCA in the training datasets (TCGA) and the validation datasets (GEO). Supplementary Table S2: List of Tolement‐related genes included in the present study. Supplementary Table S3: List of differentially expressed genes (DEGs) between ESCA and normal lung tissues based on the TCGA database. Supplementary Table S4: List of 265 Tolement‐related DEGs by taking the intersection of DEGs of ESCA and Tolement‐related genes. Supplementary Table S5: 33 candidate genes with prognostic values were screened out by the Kaplan–Meier survival analysis. Supplementary Appendix 1. The detailed protocols for cell culture, transfection, and functional assays. Supplementary Appendix 2: The detailed statistical methods. [file BMRI-2025-2605071-s001.zip › Supplementary Figure 7.pdf]
